# Selection shapes the evolution of genome size in a globally invasive plant

**DOI:** 10.1111/nph.71538

**Published:** 2026-08-28

**Authors:** Byonkesh Nongthongbam, Paul Battlay, Katherine G. Maunder, Alexandre Fournier‐Level, Michael D. Martin, John R. Stinchcombe, Kathryn A. Hodgins

**Affiliations:** ^1^ School of Biological Sciences Monash University 25 Rainforest Walk Clayton 3800 VIC Australia; ^2^ Department of Ecology and Evolutionary Biology University of Toronto 25 Willcocks St Toronto ON M5S3B2 Canada; ^3^ Department of Ecological, Plant and Animal Sciences La Trobe University Bundoora VIC 3086 Australia; ^4^ Department of Natural History, NTNU University Museum Norwegian University of Science and Technology (NTNU) 7012 Trondheim Norway

**Keywords:** *Ambrosia artemisiifolia* L. (common ragweed), biological invasion, climate adaptation, genome size, life‐history traits, population genomics, *Q*_ST_–*F*_ST_, transposable elements (TEs)

## Abstract

- Biological invasions provide powerful natural experiments for understanding how genome architecture responds to novel climatic environments. Transposable elements (TEs) can rapidly restructure genomes, yet their role in adaptive genome size evolution during invasion remains poorly understood.
- Here, we examined genome size and TE abundance in 439 individuals of globally invasive common ragweed (*Ambrosia artemisiifolia* L.) across native (North American) and invasive (European, Australian) ranges. By integrating whole‐genome resequencing, flow cytometry and trait vs genetic differentiation comparison (*Q*_ST_–*F*_ST_), we tested whether genome size evolution is shaped by selection, climate and life‐history traits.
- Genome size was significantly larger in Australian genotypes, driven by increased TE and rRNA (ribosomal RNA) abundance. Crucially, trait vs genetic differentiation comparison provided evidence of divergent selection on genome size in North American and European populations, but not in Australia. Genome size was correlated with mean annual temperature (MAT) across all ranges, linking genomic traits to environmental variables.
- Genome size evolution during invasion can be rapid, adaptive and range‐specific, with TE‐driven genome expansion emerging as a potential genomic response to the demographic and environmental pressures accompanying colonisation of novel environments.

## Graphical abstract

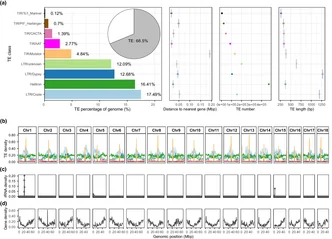

## Introduction

Genome size varies enormously across eukaryotes and is largely decoupled from gene number, reflecting extensive variation in non‐coding and repetitive DNA (Gregory, 2005; Elliott & Gregory, 2015; Slijepcevic, 2018). In angiosperms alone, genome size varies 2400‐fold (Pellicer *et al*., 2010), from the miniature genome of *Genlisea tuberosa* (1C = 63 Mbp; Zedek *et al*., 2024) to *Paris japonica* (1 C = 148.89 Gbp), the largest eukaryotic genome currently known (Fernández *et al*., 2024). Once viewed as a passive consequence of duplications, deletions and transposable element (TE) activity (Gregory, 2004; Adams *et al*., 2023), genome size is increasingly recognised as a form of structural variation with potential consequence for organismal performance and adaptation (Suda *et al*., 2015; Bilinski *et al*., 2018; Blommaert, 2020). In plants, genome size has been linked to cell division rates, development and phenology, suggesting that it may mediate responses to environmental gradients (Robinson *et al*., 2018; Blommaert, 2020). At the same time, genome size can evolve rapidly through non‐adaptive processes, including TE proliferation and genetic drift, particularly under demographic disequilibrium (Smith, 2016; Dazenière *et al*., 2022). Biological invasions, which combine strong demographic perturbations with exposure to novel environments, therefore provide powerful natural experiments for disentangling adaptive and non‐adaptive drivers of genome size evolution. Here, we quantify genome size and repetitive element variation across native and invasive populations of the globally invasive common ragweed (*Ambrosia artemisiifolia* L.), integrating genomic, climatic and phenotypic data to test how genome size evolves during rapid range expansion across broad climatic gradients.

Two non‐mutually exclusive frameworks have been proposed to explain genome size variation within species. In one framework, genome size influences traits like cell cycle duration, growth rates and phenology, such that selection acting on these traits indirectly shapes genome size along environmental gradients (Robinson *et al*., 2018; Blommaert, 2020). In the other, genome size evolves indirectly through the accumulation of TE insertions that are typically neutral or deleterious and persist under demographic regimes characterised by strong genetic drift and inefficient purifying selection, rather than through selection on genome size‐associated traits (Smith, 2016; Arkhipova, 2018; Dazenière *et al*., 2022; Marino *et al*., 2025). Environmental stressors associated with habitat shifts may further promote TE activity by disrupting epigenetic silencing mechanisms, while associated demographic bottlenecks may reduce the efficacy of purifying selection, facilitating the persistence of TE insertions (Lockton *et al*., 2008; Fedoroff, 2012; Lisch, 2013; Makarevitch *et al*., 2015). These frameworks generate contrasting expectations. Genome size evolution shaped by divergent selection imposed by local environments should produce population differentiation exceeding neutral genetic divergence and correlations with the environmental gradient, whereas genome size evolution driven primarily by drift and reduced efficacy of purifying selection is expected to yield region‐specific patterns reflecting invasion history.

Genome size is particularly relevant for invasion biology because it is linked to life‐history characteristics that determine colonisation success, establishment and persistence in novel environments (Suda *et al*., 2015; Cang *et al*., 2024). Global surveys show that invasive plant species are disproportionately drawn from lineages with smaller genomes (Suda *et al*., 2015; Guo *et al*., 2024). Patterns of genome size variation among invasive plant species have been interpreted in the context of the Large Genome Constraint (LGC) hypothesis (Bennett, 1987; Knight *et al*., 2005), which proposes that larger genomes impose a minimum threshold on cell cycle duration and generation time. This produces a triangular relationship between genome size and developmental rate: species with large genomes are excluded from fast‐growing strategies entirely, while species with small genomes can occupy both fast and slow ends of the spectrum (Šímová & Herben, 2012; Bhadra *et al*., 2023; Bureš *et al*., 2024). However, genome size‐invasion relationships are not uniformly directional: larger genomes have also been associated with slow‐growing, resource‐dominant strategies in invasive plants that establish through increased competitiveness rather than rapid colonisation (Grime & Mowforth, 1982; Grime, 1998; Guo *et al*., 2024). These patterns are largely based on interspecific comparisons and therefore provide limited insight into how genome size evolves within species during invasion.

Evidence is growing that intraspecific genome size variation is not stochastic but can have significant ecological relevance, potentially shaped by environmental filtering and natural selection across environmental gradients (Kalendar *et al*., 2000; Šmarda *et al*., 2010; Šmarda, 2010; Bilinski *et al*., 2018). Intraspecific genome size variation may reflect a complex interplay between local selection on life‐history traits and non‐adaptive processes associated with invasion, including founder events and population bottlenecks that can reduce the efficacy of purifying selection and facilitate TE accumulation (Schrader *et al*., 2014; Stapley *et al*., 2015; Mérel *et al*., 2021; Hodgins *et al*., 2025). The contrasting predictions based on cross‐species patterns and within‐species evolutionary dynamics highlight the need for intraspecific tests of genome size change during invasion, including explicit tests for selection on genome size divergence and triangular constraints predicted by the LGC hypothesis.

Common ragweed (Asteraceae) is an annual, wind‐pollinated weed, native to North America that has rapidly expanded across Europe, Australia and other regions since the nineteenth century (Knolmajer *et al*., 2024). Genomic studies indicate that the European invasion proceeded through multiple introductions, primarily from the West and Mideast North American genetic clusters, with little evidence for a reduction in effective population size (Bieker *et al*., 2022; Battlay *et al*., 2025a,b). By contrast, the more recent Australian invasion, first recorded in the early twentieth century, was primarily derived from South and Mideast North American genetic clusters and experienced a substantial reduction in effective population size consistent with a strong founder event, with a secondary restricted introduction from New England (Battlay *et al*., 2025a,b). Ragweed's invasion has exposed populations to broad climatic gradients and substantial demographic perturbations associated with range expansion, while retaining considerable standing genetic variation (Bieker *et al*., 2022; Battlay *et al*., 2025b). Remarkably, latitudinal clines in several important traits such as flowering time and plant size, initially observed in the native range, have evolved in Europe and Australia, indicating rapid and parallel post‐invasion adaptation (van Boheemen *et al*., 2019; McGoey *et al*., 2020). While previous population genomic studies have identified SNPs and structural variants (e.g. inversions, copy number variants) involved in local adaptation in this species (Battlay *et al*., 2023; Wilson *et al*., 2025), the role of genome size and TE content in shaping adaptive responses across its global range remains underexplored. Previous studies have reported genome size variation in common ragweed (Kubešová *et al*., 2010; Bai *et al*., 2012; Battlay *et al*., 2023; Hrabovský *et al*., 2024), but the evolutionary drivers and ecological consequences of this variation remain unresolved, particularly across the species’ native and invasive ranges.

We use common ragweed introductions as a replicated natural experiment to examine how genome size and repetitive DNA evolve during rapid range expansion across broad climatic gradients. By integrating whole‐genome resequencing, flow cytometry‐based genome size estimates, climatic data and life‐history traits across native and invasive ranges, we address the following specific questions: (1) How is repetitive DNA, particularly TEs, distributed across the genome, and which components contribute most to genome size variation? (2) How do genome size and TE content vary across the native and invasive ranges, and are these patterns associated with climate? (3) Does genome size divergence among populations exceed neutral genetic divergence, consistent with selection acting on genome architecture rather than drift alone? and (4) Does genome size impose a lower‐boundary constraint on developmental speed (flowering time), as predicted by the LGC hypothesis? Together, these questions allow us to evaluate whether genome size evolution during invasion is more consistent with adaptive responses to novel environments, non‐adaptive demographic processes or their interaction.

## Materials and Methods

### Samples and alignment

The primary haplotype (haplotype 1) of the diploid chromosome‐level reference genome of common ragweed (*Ambrosia artemisiifolia* L.), originally sequenced and assembled from a sample collected in Novi Sad, Serbia (45.25°N, 19.91°E), was used as reference (Battlay *et al*., 2023). To ensure accurate mapping to the nuclear genome, reads were aligned to a refined version of this reference by removing (1) scaffolds shorter than 100 kbp, (2) scaffolds with more than 20% of annotated genes of mitochondrial or chloroplast origin and (3) scaffolds containing fewer than one annotated gene per megabasepair (Mbp). The mitochondrial and chloroplast genomes were independently assembled (V. Terzer *et al*, in prep.) and added back to the reference. The resulting reference comprised two organellar genomes, 18 nuclear chromosomes and 31 additional nuclear scaffolds with a total nuclear genome size of 1.06 Gbp, compared to the unfiltered assembly of 1.1 Gbp.

We used a subset of the whole‐genome resequencing data described in Bieker *et al*. (2022) and Battlay *et al*. (2025b). The leaf tissues from 443 individuals were sampled between 2007 and 2019 and sequenced on Illumina platforms to an average depth of *c*. 7.1×. Sampling spanned the native range (North America; *n* = 179) and invasive ranges in Europe (*n* = 169) and Australia (*n* = 96). Reads were aligned to the filtered reference using the RepAdapt pipeline (https://github.com/RepAdapt/snp_calling_simple) incorporating fastp v.0.20.1 (Chen *et al*., 2018; https://github.com/OpenGene/fastp) for read trimming, bwa‐mem v.0.7.17‐r1188 (Li, 2013; https://github.com/lh3/bwa) and samtools v.1.16.1 (Li *et al*., 2009; https://github.com/samtools/samtools) for alignment, Picard Tools v.2.26.3 (Picard Toolkit, 2019; https://github.com/broadinstitute/picard) for duplicate removal and GATK v.3.8 (McKenna *et al*., 2010; https://github.com/broadgsa/gatk) for local realignment of reads around indels. Because we were interested in reads mapping to repetitive regions of the genome, we set the samtools parameter from ‐q 10 to ‐q 0 to relax the mapping quality filter and retain more mapped reads.

### Genome size estimation (sequence‐based method)

We used average read depth to estimate an effective haploid genome size (1C, Gbp) for each sample (Desvillechabrol *et al*., 2016; Pflug *et al*., 2020; Pfenninger *et al*., 2022; Natarajan *et al*., 2025). Although sequencing reads originated from diploid individuals, these were mapped to a primary haplotype assembly, allowing us to infer the size corresponding to a single, haploid set of chromosomes. First, the mean genome‐wide coverage depth was calculated from aligned BAM files using the samtools depth ‐aa command in samtools v.1.16.1, with organelle scaffolds excluded. We scaled genome‐wide depth by the mean depth over a set of putatively single‐copy loci from the Benchmarking Universal Single‐Copy Orthologs (BUSCO) eukaryota *odb10* dataset (Simão *et al*., 2015). Nevertheless, our reference genome contained 72 apparent duplications of BUSCO genes (Battlay *et al*., 2023), which we removed, leaving 183 genes for this analysis. The mean gene depth across these genes was calculated using samtools depth on a BED file of their genomic coordinates generated from the genome's gene annotation, documented in Battlay *et al*. (2023). Genome size for each sample was then estimated by dividing the mean genome‐wide depth by the mean BUSCO depth, reflecting coverage over single‐copy regions. We used a coverage‐based adaptation of the Lander‐Waterman equation (Lander & Waterman, 1988). In our case, genome size (G) was calculated as the ratio of the mean whole‐genome depth ($\bar{\mathrm{WGD}}$) to the mean BUSCO depth ($\bar{\mathrm{BD}}$):

$$G=\frac{\bar{\mathrm{WGD}}}{\bar{\mathrm{BD}}}$$

We also assessed several sequencing quality metrics including sequencing coverage depth, insert size, read length, mapping quality thresholds, callable base depth, BUSCO gene depth variance and PCR clonality for their potential effect on genome size estimation Supporting Information (Notes S1; Figs S1–S3). We then removed samples with genome sizes more than three SD from the mean genome size (Fig. S4), resulting in 439 samples retained for downstream analyses (Table S1).

### Flow cytometry

Sequence‐based genome size estimates were independently validated using flow cytometry (*n* = 40) with *Manihot esculenta* as an internal control. Detailed protocols for sample preparation, calibration and fluorescence analysis are provided in the Notes S2.

### TE annotation

TEs were annotated on the primary haplotype of the filtered reference genome (Battlay *et al*., 2023) using the EDTA (Extensive *de‐novo* TE Annotator) pipeline (Ou *et al*., 2019; https://github.com/oushujun/EDTA), with the ‐anno function and default parameters. These elements were then processed to generate a non‐redundant, curated TE library. This step clusters TEs into families while reducing redundancy, based on a modified 80‐80‐80 Wicker rule (Wicker *et al*., 2007), which groups elements that share ≥80% identity over ≥80% of their length and belong to the same superfamily. The curated TE library was then used to mask the genome using RepeatMasker v.4.1.1 (Smit *et al*., 2015; http://repeatmasker.org). Subsequently, the masked genome was re‐annotated with the same TE library using EDTA's ‐anno function to produce a comprehensive TE annotation. For downstream analyses, we used the split annotation BED file, which retains only non‐overlapping TE annotations, ensuring that any particular genomic region is assigned to only a single TE feature.

### Ribosomal RNA annotation

RNAmmer v.1.2 (Lagesen *et al*., 2007) was used to annotate eukaryotic ribosomal RNA (rRNA) on the reference genome, including subunits 28S, 18S and 8S. RNAmmer annotates rRNA using hidden Markov models (Rabiner & Juang, 1986), capturing structural features from multiple alignments of rRNA database sequences.

### Non‐overlapping TE annotation and abundance estimation

A non‐overlapping annotation file was generated by integrating annotations for TEs, rRNA, simple repeats and low‐complexity regions, with the following prioritisation: rRNA > known TEs > simple repeats > low‐complexity regions (adapted from Kreiner *et al*., 2023). Since EDTA did not identify simple and low‐complexity repeats, their corresponding annotation BED files were obtained from the RepeatModeler output generated in our previous study (Battlay *et al*., 2023). Sequences shorter than or equal to 20 bp were excluded to minimise false positives. The resulting curated annotation file was used for downstream analyses.

To estimate TE abundance, we employed a read‐depth‐based approach analogous to that used for genome size estimation. Specifically, the abundance of each TE family was calculated as the mean depth of reads mapping to each TE annotation, normalised by the mean read depth over BUSCO genes. This provides an estimate of the relative copy number for each TE family, expressed as:

$$\mathrm{TE}=\frac{\bar{\mathrm{TED}}}{\bar{\mathrm{BD}}}$$

where (TE) is the abundance/copy number of a TE family, ($\bar{\mathrm{TED}}$) is the mean read depth over TE annotations and ($\bar{\mathrm{BD}}$) is the mean read depth over single‐copy BUSCO genes. We then multiplied this normalised copy number by the length of each TE family (Table S2) to calculate the per‐individual total repeat abundance in base pairs (bp). We also assessed the effect of sequencing coverage depth on TE or rRNA abundance estimates by regressing whole‐genome average depth against the estimated abundance of each TE or rRNA family across all samples (Notes S1; Fig. S5).

The size and quantity of TEs were directly visualised using non‐overlapping annotations based on counts and base pair ranges for each superfamily. To determine the distance to the nearest gene, bedtools closest 2.31.0 (Quinlan & Hall, 2010; https://github.com/arq5x/bedtools2/releases) was employed, identifying the closest gene to each repeat element and reporting the distance using the ‐d option. The fraction of each 1 Mbp genomic window occupied by different types of repeats was computed using the genomicDensity() function from the R package circlize v.0.4.16 (Gu *et al*., 2014; https://jokergoo.github.io/circlize_book/book/). Variance and coefficient of variation of repeat abundance were calculated in R.

### Comparison of trait vs genetic differentiation

To understand which evolutionary forces drive variation in genome size among populations, we performed a *Q*_ST_ *–F*_ST_ comparison as the primary analysis (Prout & Barker, 1993; Spitze, 1993; Whitlock, 2008). This analysis utilised whole‐genome resequencing data from 289 individuals (representing all populations with *n* ≥ 2) based on the variant call format (VCF) provided by Battlay *et al*. (2025b). Two complementary approaches, a *P*_ST_ *–F*_ST_ comparison (Sæther *et al*., 2007; Whitlock, 2008), which assesses phenotypic differentiation relative to neutral expectations, and *Q*_PC_ analysis (Josephs *et al*., 2019), which tests for adaptive divergence along axes of genetic relatedness without requiring predefined populations, are described in Note S3.

To estimate *Q*_ST_, we first constructed a genomic relatedness matrix (GRM) from 50 000 LD‐pruned ($r^{2}$ = 0.5), MAF‐filtered (≥ 0.01) SNPs excluding genic regions and known haploblocks, using the VanRaden (2008) method implemented in R\AGHmatrix (Amadeu *et al*., 2023). We then modelled genome size using a Bayesian animal model implemented in R\MCMCglmm (Hadfield, 2010), with the GRM used to parameterise the structure of the covariance of the genotypic effect. Individual genomic breeding values were extracted from each of the posterior distributions from 1000 MCMC samples and used to calculate a posterior distribution of *Q*_ST_ directly, without collapsing to a single point estimate. Within each geographical range (North America, Europe, Australia), breeding values were partitioned by population and *Q*_ST_ was calculated as (Lande, 1992; Spitze, 1993; Whitlock, 2008):

$$Q_{\mathrm{ST}}=\frac{\sigma_{\mathrm{GB}}^{2}}{\left(\sigma_{\mathrm{GB}}^{2}+2\times \sigma_{\mathrm{GW}}^{2}\right)}$$

where $\sigma_{\mathrm{GB}}^{2}$ is the additive genetic variance among populations and $\sigma_{\mathrm{GW}}^{2}$ the additive genetic variance within‐population. We report the mean of the *Q*_ST_ posterior distribution and 95% credible interval. Full MCMC settings, convergence diagnostics and heritability estimation details are provided in Notes S3a.

For each range, observed *Q*_ST_ was compared to a null distribution of *F*_ST_ values generated from putatively neutral, LD‐pruned sites, randomly sampled from non‐genic and non‐structural variant regions of the VCF mentioned above; these SNPs were explicitly excluded from those used in GRM construction, ensuring independence between the neutral reference distribution and the markers used to estimate quantitative genetic parameters. SNPs were filtered for minor allele frequency ≥ 0.05 and LD‐pruned (50‐kbp window, 5‐SNP step, *r* ^2^ = 0.5) using PLINK v.1.9. Overlapping SNPs across all ranges were then identified using bcftools isec (Danecek *et al*., 2021; http://github.com/samtools/bcftools), and 10 000 SNPs were randomly selected for the calculation of pairwise Weir and Cockerham *F*_ST_ for each range in vcftools (Weir & Cockerham, 1984; https://github.com/vcftools/vcftools). *Q*_ST_ values exceeding the 99^th^ percentile of the neutral *F*_ST_ distribution were interpreted as evidence of trait divergence exceeding neutral expectations, consistent with divergent selection. Values falling between the 1^st^ and 99^th^ percentiles were considered consistent with genetic drift alone. Values in the lower 1^st^ percentile of the neutral *F*_ST_ distribution indicate reduced among‐population divergence relative to neutral expectations and may reflect stabilising or parallel selection, high within‐population variance or limited statistical power, and were therefore interpreted cautiously (Whitlock & Guillaume, 2009).

### Principal component analysis

We performed principal component analysis (PCA) on genome‐wide SNPs (VCF provided in Battlay *et al*., 2025b) to capture neutral population structure and account for its effects in the analyses of genome size and life‐history traits. Separate PCAs were conducted for the overall analysis (439 samples) and the phenotypic analysis (221 samples). The detailed method on PCA analyses is provided in Notes S4.

### Polygenic value estimation of flowering time

Polygenic values (PGV) for flowering time were estimated using flowering time‐associated loci and effect sizes derived from a previous genome‐wide association study (GWAS; Battlay *et al*., 2025b), with detailed methodology provided in Notes (S5).

### Statistical analysis

We conducted all statistical analyses in R (v.4.4.2). Most figures utilised the ggplot2 package (Wickham, 2016) for visualisation.

To explore differences in genome size and TE abundance across the native and two invasive ranges, we fitted linear mixed models (LMMs), implemented in the R\lme4 package v.1.1–36 (Bates *et al*., 2015; https://github.com/lme4/lme4):

$$Y\sim \text{Range}+\text{PC1}+\text{PC2}+\left(1|\text{Population}\right)$$

where Y is the trait (genome size or abundance of different TE families). Range was included as a fixed effect, and Population as a random intercept. Principal components (PC1 and PC2) derived from genome‐wide SNP data were included as fixed effects to account for background population structure in analyses where SNP data were available. As a result, these terms were not included for analyses based solely on flow cytometry‐based genome size measurements. Model assumptions were evaluated by visual inspection of residual plots. The significance of fixed effects was assessed using Type III Wald F‐tests implemented with the car package (Fox & Weisberg, 2018) applied to linear mixed‐effects models fitted with lme4. Given a significant association with geographic range, *post hoc* tests on estimated marginal means (EMMs) were conducted using the emmeans package (v.1.10.7; Lenth, 2023; https://cran.r‐project.org/package=emmeans) to examine pairwise differences. EMMs were calculated with the emmeans() function, and pairwise comparisons were performed using the pairs() function, applying the Tukey Honest Significant Difference (HSD) test.

To distinguish the roles of ancestry (putative source population) from that of post‐invasion changes in genome size, we compared the genome size of invasive range populations against each of the four spatio‐genetic clusters in the native range. We used the same LMM framework, ANOVA and *post hoc* pairwise comparisons described above.

We further investigated the drivers of genome size, specifically the contributions of TE content and environment, and explored its relationship with phenotypic traits. Given the notably higher genome size and TE content in Australian populations, we tested the contribution of specific TE families and rRNA abundance to intraspecific genome size variation. To do so, we fitted linear mixed‐effects models using the following R model formula:

$$\text{Genome size}\sim X+\text{PC1}+\text{PC2}+\left(1|\text{Population}\right)$$

where genome size is the response variable, X is the abundance of different TE families and Population represents a random intercept for each population. PC1 and PC2 were included as fixed effects as previously described.

To understand the relationship between climate and genome size, we focused on WorldClim (Fick & Hijmans, 2017) variable mean annual temperature (MAT) out of all other 18 variables (Table S3) because it has been linked with genome size variation in previous studies (Guo *et al*., 2024; Meyerson *et al*., 2024; Hrabovský *et al*., 2024). Bioclimatic variables at our sampling locations were highly collinear, with the first principal component (PC1) explaining *c*. 48% of the total climatic variance and showing a strong correlation with MAT (*r* = 0.84; Notes S6; Fig. S6). Accordingly, MAT was used as a biologically interpretable proxy for the dominant climatic gradient. We fitted linear mixed‐effects models using the following R model formulas:

Base model,

$$\text{Genome size}\sim \mathrm{MAT}+\text{Range}+\text{PC1}+\text{PC2}+\left(1|\text{Population}\right)$$

Interaction model,

$$\text{Genome size}\sim {\mathrm{MAT}}^{*}\;\text{Range}+\text{PC1}+\text{PC2}+\left(1|\text{Population}\right)$$

where MAT, Range, PC1 and PC2 were treated as fixed effects and Population as a random intercept. The base model included geographic range as a fixed effect to control for range‐specific differences, while the interaction model tested whether the effect of MAT on genome size differed among ranges.

To test the LGC hypothesis, that larger genomes impose a lower boundary on developmental speed rather than shifting the mean, we first fitted a linear mixed model of flowering time on genome size, which revealed no significant effect on the mean. To test boundary constraints at tails of the distribution, we also fitted Linear Quantile Mixed Models (LQMM; lqmm R package) at five quantiles (*τ* = 0.10, 0.25, 0.50, 0.75, 0.90; Notes S7).

## Results

### Transposable element landscape and abundance

**Fig. 1:**
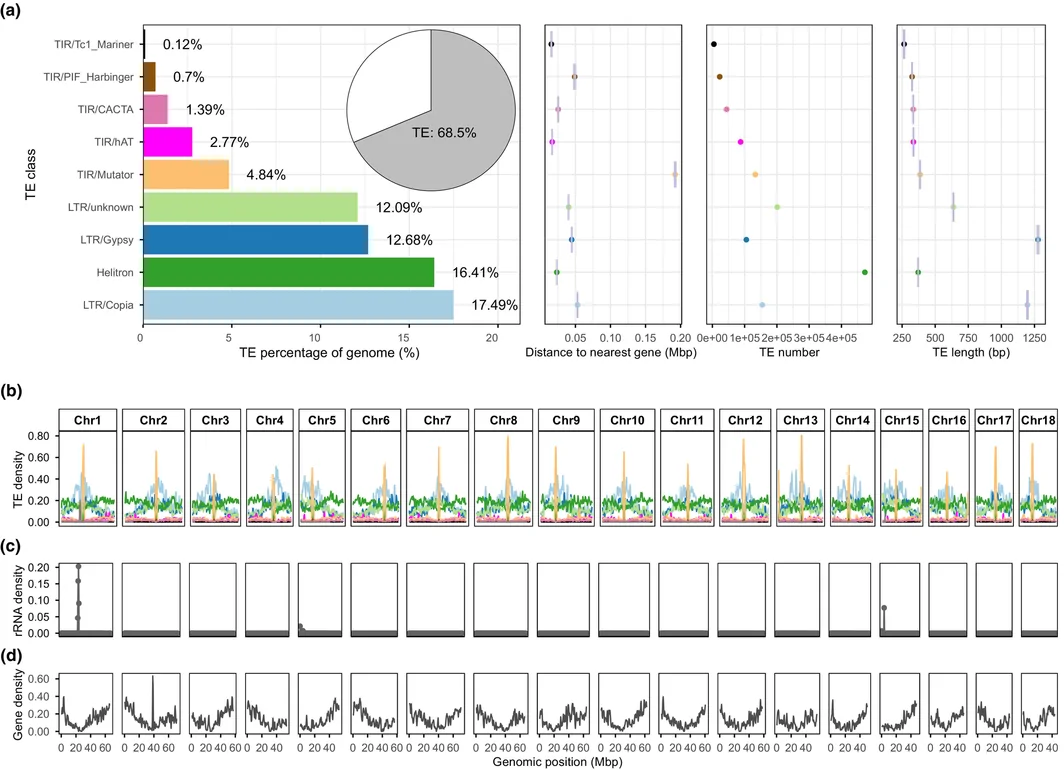
Abundance, genomic distribution and structural characteristics of transposable elements (TEs) and ribosomal RNA (rRNA) genes across the common ragweed (*Ambrosia artemisiifolia* L.) reference genome. (a) Proportion of the genome represented by various TE classes (LTR/Copia, LTR/Gypsy, LTR/unknown, Helitron, TIR/Mutator, TIR/hAT, TIR/CACTA, TIR/PIF_Harbinger, TIR/Tc1_Mariner; colour‐coded as shown). Adjacent scatter plots show, for each TE class, mean distance to nearest genes (Mbp), total copy number and mean element length (bp); error bars represent standard error (SE). (b) TE density (proportion of each window occupied by TEs, coloured by TE class as in a) across all 18 chromosomes, calculated in sliding 1Mbp windows. (c, d) rRNA and gene density across the genome, respectively, both calculated in sliding 1 Mbp windows and plotted alongside the corresponding TE density windows.

TEs constituted 68.5% of the common ragweed genome, consistent with prior estimates (Battlay *et al*., 2023). The TE landscape was dominated by Long Terminal Repeat (LTR) retrotransposons (42.3%; 448 Mbp), primarily comprising LTR/Copia (17.5%), LTR/Gypsy (12.7%) and LTR/Unknown (12.1%) elements (Fig. 1a, panel 1). Other abundant families included Helitrons (16.4%) and TIR transposons such as TIR/Mutator (4.8%), TIR/hAT (2.8%) and TIR/CACTA (1.4%), with less abundant families detailed in Table S2 (Fig. 1a, panel 1). The genomic distribution of TEs revealed distinct family‐specific trends with respect to distance to genes, copy number and element size (Fig. 1a, panels 2–4; Table S2). TIR/Tc1_Mariner elements were located closest to genes (mean distance = 15 620 ± 458 bp) while TIR/Mutator elements were the most distal (mean distance = 192 314 ± 861 bp), suggesting differential impacts on gene regulation (Fig. 1a, panel 2; Table S2). In terms of copy number, Helitrons were the most abundant (473 277 insertions) while TIR/Tc1_Mariner were the least frequent (4859 copies; Fig. 1a, panel 3; Table S2). LTR/Gypsy and LTR/Copia were the longest elements on average (1277 ± 6 bp and 1197 ± 5 bp, respectively), while TIR/Tc1_Mariner were the shortest (265 ± 4 bp; Fig. 1a, panel 4; Table S2). TE density was markedly heterogeneous across chromosomes, higher in pericentromeric and central regions (Fig. 1b). Ribosomal RNA elements showed local enrichment on chromosomes 1, 5 and 15, with a notable expansion on chromosome 5, and slightly depleted from regions with high TE density (Spearman ρ = −0.11, *P* = 0.0002; Fig. 1c). TE‐rich regions were consistently gene poor (Spearman ρ = −0.78, *P* < 2.2 × 10^−16^; Fig. 1d).

### Geographic variation in genome size

**Fig. 2:**
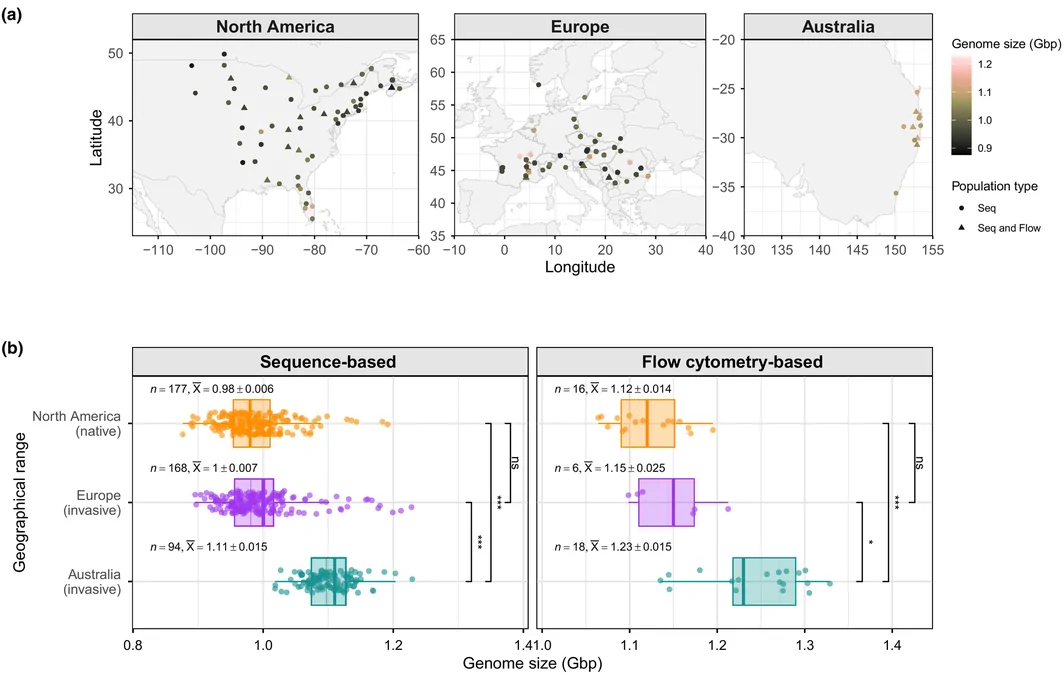
Geographical patterns of genome size variation in common ragweed (*Ambrosia artemisiifolia* L.) across its native North American range and two independent invasive ranges (Europe and Australia). (a) Map showing the geographical locations of sampled populations used in this study and their genome size estimates; circles represent populations with sequence‐based estimates, and triangles indicate populations with both sequence‐ and flow cytometry‐based estimates. (b) Genome size (Gbp) variation among geographical ranges; native (North America) and invasive (Europe and Australia) from two independent analyses: genomic read depth‐based (left panel) and flow cytometry‐based (right panel). Boxes show the interquartile range (IQR, 25^th^–75^th^ percentile) of the raw data, with whiskers extending to 1.5 × IQR; individual values are overlaid as jittered points. The solid horizontal line within each box marks the estimated marginal mean from a linear mixed‐effects model with geographic range, genetic principal components (PC1 and PC2) as fixed effects and population as a random intercept. Below each box, we report sample size (*n*) and the model‐estimated mean ($\bar{X}$) ± SE. Asterisks denote statistical significance from Tukey's honestly significant difference (HSD) *post hoc* pairwise comparisons (***, *P* < 0.001; **, *P* < 0.01; *, *P* < 0.05; ns, not significant).

From our sequence‐based estimates, the haploid genome size of common ragweed ranged from 0.88 to 1.23 Gbp, with a mean ± SE of 1.01 ± 0.0034 Gbp (Fig. 2a). Common ragweed exhibited a pronounced regional difference in genome size across its native and its two introduced ranges (linear mixed‐effects model: *F*_2,138.8_ = 25.39, *P* = 4.018 × 10^−10^, *R* ^2^ = 0.61; Table 1). *Post hoc* tests confirmed that Australian genomes were significantly larger than European and North American genomes (Tukey's HSD; diff_AUSvsEUR = 100 Mbp, *P* < 0.0001; diff_AUSvsNAM = 120 Mbp, *P* < 0.0001; estimated marginal means ± SE: AUS = 1.1 ± 0.015 Gbp, EUR = 1.00 ± 0.007 Gbp, NAM = 0.98 ± 0.006 Gbp; Fig. 2b, left panel). Conversely, genome sizes between Europe and North America did not differ significantly (diff_EURvsNAM = 20 Mbp, *P* = 0.26) (Fig. 2b).

**Table 1:** Linear mixed model testing the effects of geographical range and genetic principal components (PC1 and PC2) on genome size in common ragweed, with population as a random intercept.

Fixed effect	*F*	Df	Df.res	*P*
Range	25.3871	2	138.794	4.018 × 10^−10^
PC1	0.0027	1	210.657	0.9590
PC2	2.5931	1	240.659	0.1086

*F*‐values, numerator degrees of freedom (Df), Kenward–Roger‐approximated denominator degrees of freedom (Df.res) and P‐values are shown for each fixed effect, from type III Wald F‐tests (ANOVA, type = 3, test = ‘F’). PC1 and PC2 are the first and second principal components of genetic population structure (see Supporting Information Notes S4). *P* < 0.05 was considered statistically significant.

### Validation of sequence‐based genome size variation with flow cytometry

Sequencing‐based estimates of haploid genome size were independently validated using flow cytometry, with calibration details provided in Notes S2 (also see Fig. S7). The resulting haploid genome size estimates for common ragweed were approximately normally distributed (Fig. S8), with a mean of 1.18 ± 0.01 Gbp, ranging from 1.06 to 1.33 Gbp (Table S4). At the population level, flow cytometry and sequencing‐based genome size estimates were highly correlated (absolute Pearson's *r* = 0.938, Fig. S9). Flow cytometry revealed significant differences in genome size across geographic ranges (*F*_2,22.627_ = 14.62, *P* = 8.4 × 10^−5^), consistent with the sequencing‐based findings (Fig. 2b, right panel).

### Genome size difference between the invasive ranges and their corresponding native source populations

**Fig. 3:**
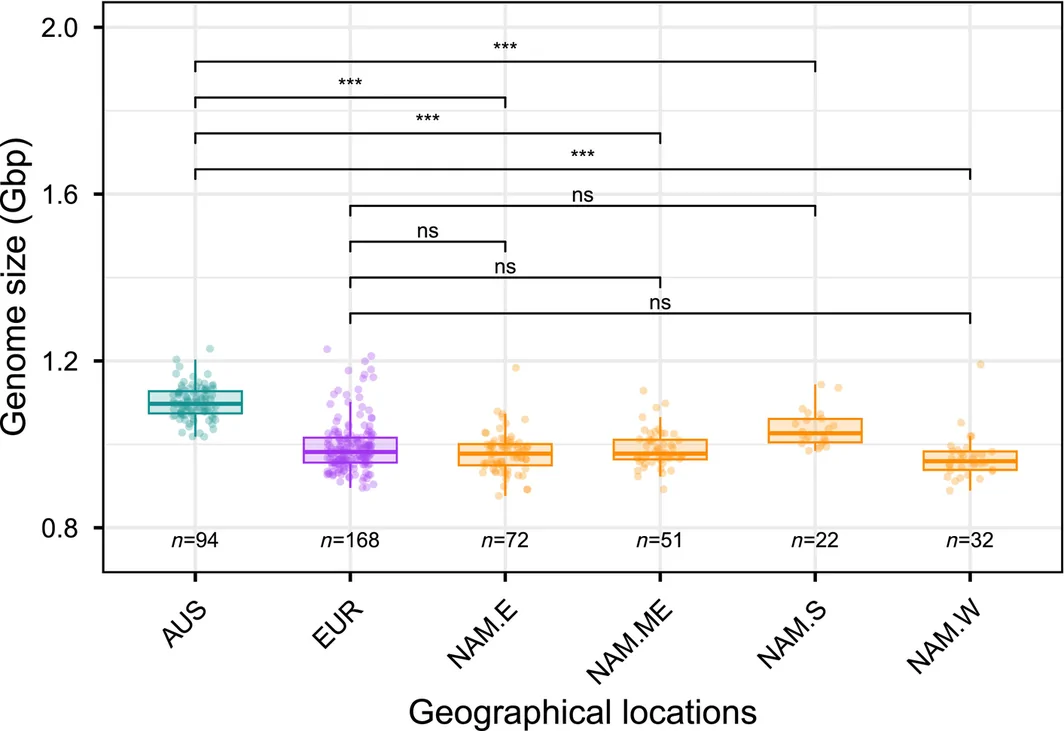
Genome size comparisons between two invasive ranges of common ragweed (*Ambrosia artemisiifolia* L.) and their putative native source populations in North America. Genome size (Gbp) estimates from sequence‐based data are shown for Australia (AUS), Europe (EUR) and four North American spatio‐genetic clusters: mid‐east (NAM.ME), south (NAM.S), west (NAM.W) and east (NAM.E). Boxes show the interquartile range (IQR, 25^th^–75^th^ percentile) of the raw data, with whiskers extending to 1.5 × IQR and individual values are overlaid as jittered points. The solid horizontal line within each box marks the estimated marginal mean from a linear mixed‐effects model with geographic locations (under comparisons), the first two genetic principal components (PC1 and PC2) as fixed effects and population as a random intercept. Sample size (*n*) is reported for each cluster. Asterisks denote statistical significance from Tukey's honestly significant difference (HSD) *post hoc* pairwise comparisons (***, *P* < 0.001; **, *P* < 0.01; *, *P* < 0.05; ns, not significant).

As previously described, genomic analysis suggested that the two invasion ranges were introduced from distinct native source clusters. We therefore compared the genome size of invasive ranges against their likely native source populations. Significantly larger genome sizes were observed in the Australian invasive range compared to its putative native sources (North American South and Mid‐East) and other native North American populations (Fig. 3; Table S5). By contrast, the European invasive range showed overall similar genome sizes to native North American populations and their putative native sources. These patterns in genome size mirrored similar observations for the abundance of most TE and rRNA families (Fig. S10).

### Geographical pattern of class‐specific TE abundance

**Fig. 4:**
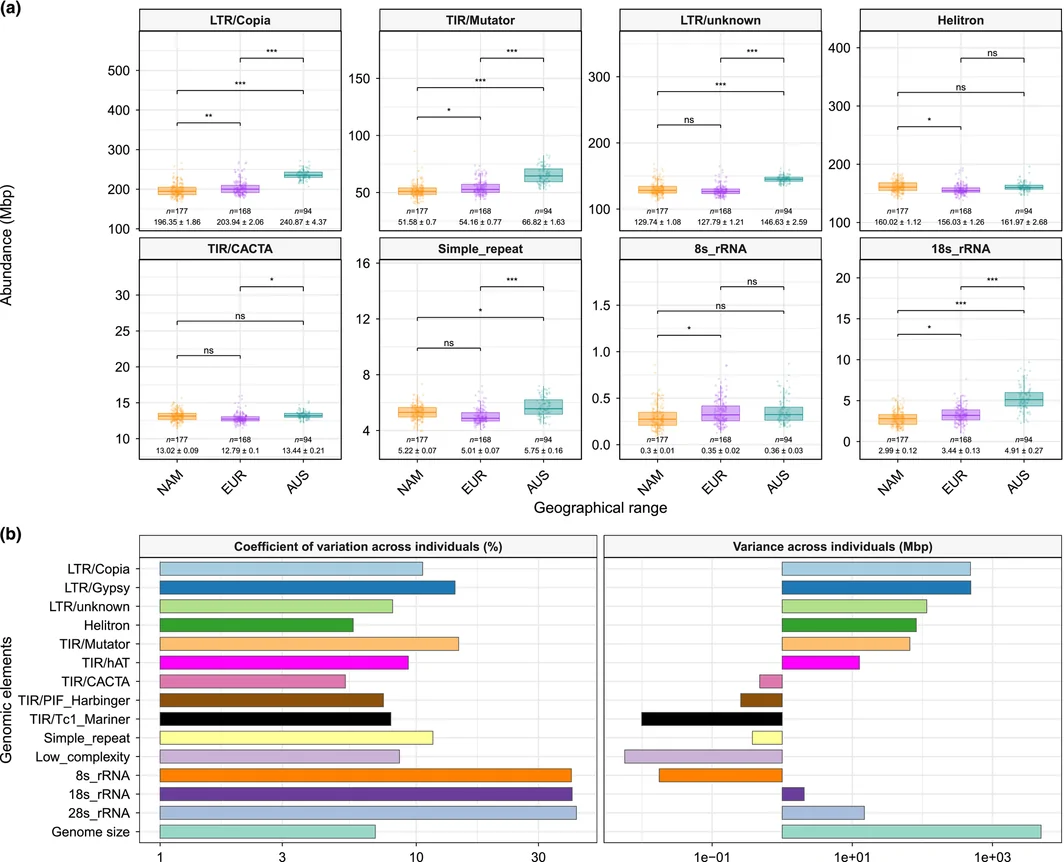
Characterisation of TE and rRNA abundance variation among individual common ragweed (*Ambrosia artemisiifolia* L.) samples. (a) Abundance (Mbp) of TEs and rRNAs families among individuals from different geographical ranges: North America (NAM), Europe (EUR) and Australia (AUS). Boxes show the interquartile range (IQR, 25^th^–75^th^ percentile) of the raw abundance data, with whiskers extending to 1.5 × IQR and individual values are overlaid as jittered points. The solid horizontal line within each box marks the estimated marginal mean from a linear mixed‐effects model with geographic range, genetic principal components (PC1 and PC2) as fixed effects and population as a random intercept, with sample size (*n*) and the model‐estimated mean ($\bar{X}$) ± SE reported alongside each group. Asterisks denote statistical significance from Tukey's honestly significant difference (HSD) *post hoc* pairwise comparisons (***, *P* < 0.001; **, *P* < 0.01; *, *P* < 0.05; ns, not significant). (b) Measures of variability in TE and rRNA abundance across individuals, summarised as the coefficient of variation (CV; left); Variance of log‐transformed abundance (V; right).

To assess the potential for TE and rRNA variation to contribute to intraspecific adaptation in common ragweed, we quantified the abundance of TE and rRNA families across ranges. The mean ± SE length of genome represented by each TE family across individuals mirrored the rank order observed in our reference genome: LTR/Copia (208 ± 1.05 Mbp), Helitron (159 ± 0.43 Mbp), LTR/Gypsy (156 ± 1.06 Mbp), LTR/unknown (133 ± 0.51 Mbp), TIR/Mutator (55.6 ± 0.39 Mbp), TIR/hAT (38.2 ± 0.17 Mbp), TIR/CACTA (13.1 ± 0.03 Mbp), TIR/PIF_Harbinger (6.8 ± 0.02 Mbp), Simple Repeats (5.27 ± 0.03 Mbp), TIR/Tc1_Mariner (1.25 ± 0.01 Mbp) and Low Complexity (0.88 ± 0.01 Mbp) (Fig. S11). Despite this compositional consistency, Australian samples exhibited higher abundances of TEs across most families compared to European and North American samples (Figs 4a, S12). LMMs confirmed a significant effect of geographic range on most TE and rRNA families (excluding TIR/hAT and TIR/PIF_Harbinger; *P* < 0.05; Table S6). Pairwise comparisons revealed that most TE and rRNA families, particularly LTR/Copia, LTR/Gypsy and TIR/Mutator, were significantly more abundant in Australia than in either Europe or North America (Table S6). Among all repeat types, rRNA families (8S, 18S, 28S) exhibited the highest coefficient of variation (CV), likely reflecting their confinement to a few clustered loci where small absolute changes yielded large proportional variance (Fig. 4b). By contrast, highly abundant TEs such as LTR/Copia, LTR/unknown and Helitron showed lower relative variance but substantial absolute copy number variation, suggesting these elements contribute notably to genomic differences among individuals.

### TE and rRNA abundance significantly contribute to genome size variation

The abundance of specific TEs and rRNA predicted genome size well (Table S7). Our LMMs revealed strong and significant associations between genome size and TE abundance, particularly LTR and TIR families (Table S7). Furthermore, consistent with TE abundance, rRNA subunit abundance, except 18S‐rRNA, was likewise significantly correlated with genome size (Table S7).

### Genome size selection analysis

To test whether divergent selection shaped genome‐size variation, we performed *Q*_ST_–*F*_ST_ comparisons. We first confirmed that genome size variation has a strong additive genetic basis with a posterior mean SNP‐based heritability of $h^{2}$ = 0.886 (95% CI: 0.772–0.953).

**Fig. 5:**
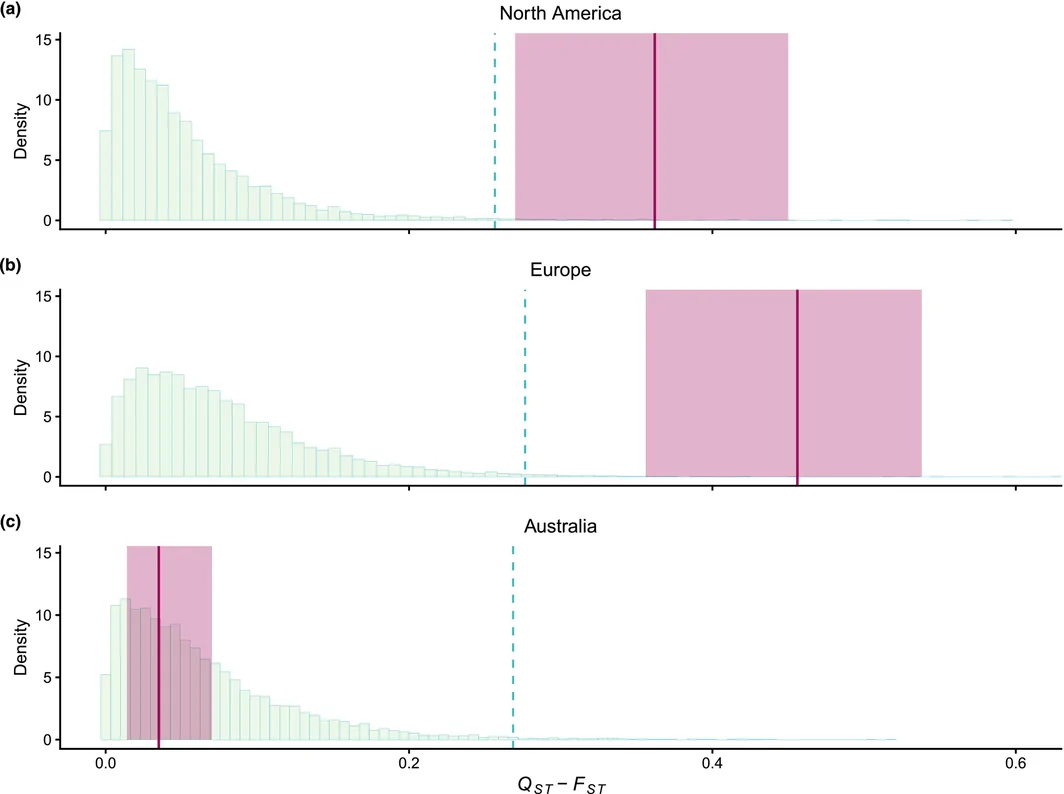
Quantitative genetic differentiation (*Q*_ST_) for genome size compared against the neutral distribution of genetic differentiation (*F*_ST_) across the native and invasive ranges of common ragweed (*Ambrosia artemisiifolia* L.): (a) North America (native), (b) Europe (invasive) and (c) Australia (invasive). *Q*_ST_ was derived from genomic breeding values estimated for genome size. In each panel, the background green histogram shows the null distribution of neutral *F*_ST_ values (a standard population‐genetic measure of genetic differentiation among populations based on genome‐wide SNP markers), with the teal dashed vertical line marking its 99^th^ percentile. The shaded light magenta box represents the 95% credible interval of the *Q*_ST_ posterior distribution, and the solid magenta line represents the posterior *Q*_ST_ mean. A posterior mean *Q*_ST_ value exceeding the 99^th^ percentile of the neutral *F*_ST_ distribution indicates divergent selection on genome size.

In North America and Europe, additive genetic differentiation (*Q*_ST_) significantly exceeded neutral expectations. In North America, the posterior mean *Q*_ST_ (0.362) far exceeded the 99^th^ percentile of the neutral *F*_ST_ distribution (0.26; Fig. 5a). Similarly, in Europe, *Q*_ST_ (0.456) exceeded the 99^th^ percentile of the neutral *F*_ST_ distribution (0.28; Fig. 5b), providing strong evidence that divergent selection is driving genome size differences among populations in both ranges. By contrast, populations in Australia exhibited almost no differentiation; the posterior mean *Q*_ST_ was 0.035, falling well below the upper neutral *F*_ST_ thresholds (0.27; Fig. 5c), providing no evidence that divergent selection has contributed to genome size differentiation within this range. Although *Q*_ST_ was low, *Q*_ST_ also exceeded the 1^st^ percentile *F*_ST_ (0.0009), precluding inference of stabilising selection.

These findings were further corroborated by two complementary approaches: *P*_ST_ *–F*_ST_ comparison and *Q*_PC_ analysis, both of which yielded consistent conclusions across all three ranges (Notes S3). Together, these results provide robust evidence for the divergence of genome size during colonisation of North America and Europe, while variation in Australia remains inconsistent with divergent selection.

### Mean annual temperature predicts genome size

Our models revealed strong among‐range differences in genome size and support for an overall association with temperature. In the main‐effects model, both range and MAT significantly predicted genome size (range: *F*_2,129.73_ = 18.79, *P* = 6.81 × 10^−8^; MAT: *F*_1,144.86_ = 8.2482, *P* = 0.0047; Table S8), with warmer climates associated with larger genomes across ranges. Consistent with this, Australian populations occurring in warm environments had the largest genomes on average, whereas European populations sampled from cooler climates had the smallest. The interaction between MAT and range was not significant (*F*_2,83.09_ = 0.38, *P* = 0.68; Table S8), indicating that the positive association between temperature and genome size did not differ detectably among ranges.

### Genome size does not act as a lower‐boundary constraint on phenology

LQMM analysis across all ranges and within individual ranges revealed no significant associations at lower quantiles (*τ* = 0.10–0.25; all *P* > 0.34), inconsistent with a lower‐boundary constraint on development. While a significant effect was detected at the median (*τ* = 0.50, *P* = 0.049) across all ranges combined, range‐specific models showed no significance in North America or Europe, and signals were restricted to the upper tail (*τ* = 0.90, *P* = 0.027) in Australia (Tables S9 and S10). Given that only two of twenty quantile tests were nominally significant, these results provide little evidence for a consistent effect of genome size on flowering time and do not support a lower‐boundary constraint on flowering time.

## Discussion

The remarkable success of invasive species often involves rapid evolutionary adaptations (Prentis *et al*., 2008; Suda *et al*., 2015; Wu *et al*., 2019; Hodgins *et al*., 2025) and our study of common ragweed genome size evolution reveals a particularly intriguing case. We found that Australian invasive populations possess significantly larger genomes compared to both their native North American and invasive European counterparts, a difference largely attributable to the increased abundance of TEs. *Q*_ST_ *–F*_ST_ comparisons with the support from *P*_ST_ *–F*_ST_ and *Q*_PC_ analyses in North America and Europe further revealed patterns consistent with divergent selection on genome size, suggesting that genome size divergence is unlikely to be explained by neutral processes alone. Genome size showed evidence of climate structuring, consistent with a role in climate adaptation. Below, we discuss these results in light of the genetic mechanisms underlying TE landscapes, evaluate our findings in the context of hypotheses linking genome size to invasion success, and examine the relative roles of local selection and demographic history in shaping genome size variation across native and invasive ranges.

### The common ragweed TE landscape: a dynamic interplay of composition, context and organisation

We provide a comprehensive analysis of the TE landscape in common ragweed, revealing the pattern of composition, genomic context and population‐level abundance variation. The common ragweed genome is replete with TEs, dominated by LTR retrotransposons (particularly *Copia* and *Gypsy*), consistent with patterns observed in other plant genomes (e.g. Arabidopsis (Lian *et al*., 2024), maize (Chen *et al*., 2023), sunflower (Badouin *et al*., 2017) and *Amaranthus* species (Kreiner *et al*., 2023)). The non‐random distribution of TEs, particularly the enrichment of LTR elements in chromosome centres that are relatively gene poor, is likely a consequence of these regions serving as ‘safe havens’ where selection against TE insertions is less effective due to inherently low recombination rates (Kent *et al*., 2017; Huang *et al*., 2025). Furthermore, our analysis reveals a clear non‐random genomic distance of TE insertions to genes: elements like TIR/Tc1_Mariner are found nearest to genes, while TIR/Mutator elements are typically located farthest from them (Fig. 1a). This differential localisation suggests varying selective pressures depending on their impact on nearby genes and their functional importance (Hirsch & Springer, 2017; Schrader & Schmitz, 2019). Together, these TE‐specific patterns of accumulation and retention provide a mechanistic basis for the substantial genome size differences we observe among ranges and reinforce the idea that TE dynamics can contribute to rapid genome evolution during invasion (Schrader *et al*., 2014; Stapley *et al*., 2015; Dennenmoser *et al*., 2017; Goubert *et al*., 2017; Niu *et al*., 2019). Future work combining population‐level TE polymorphism data and environmental associations will be needed to test whether changes in specific TE families are directly linked to range expansion or climate adaptation.

### Does the LGC hypothesis extend to intraspecific genome size evolution during invasion?

Although intraspecific genome size variation is well documented in flowering plants (Greilhuber, 2005), including in Asteraceae (Suda *et al*., 2007; Slovák *et al*., 2009; Dirkse *et al*., 2014), the ecological and evolutionary drivers of this variation, particularly in invasive species, remain poorly understood. Drivers of genome size include polyploidy, demography and mating system shifts, which can change effective population size (*N*_e_) and thus the efficacy of selection, and selection on cell size or growth rate, which can alter genome size and influence life‐history traits (Glémin, 2007; Lefébure *et al*., 2017; Jiang *et al*., 2024). Invasive species tend to have smaller genomes (Suda *et al*., 2015; Pyšek *et al*., 2023; Guo *et al*., 2024), a pattern often interpreted as advantageous through faster cell cycles, earlier germination and higher growth rates which in turn enable their rapid spread during invasions (Knight *et al*., 2005; Kubešová *et al*., 2010; Suda *et al*., 2015). This negative correlation between genome size and invasiveness has been interpreted in the context of the LGC hypothesis (Knight *et al*., 2005; Kubešová *et al*., 2010; Pyšek *et al*., 2023), which proposes that larger genomes impose physiological and developmental constraints through their effects on cell size, cell cycle duration and generation time (Bennett, 1972; Cavaller‐Smith, 1985), indirectly limiting the rapid growth and reproduction that favour establishment and spread in novel habitats.

By substantially expanding population‐level estimates of genome size across native and invasive ranges, our study provides a nearly global perspective on common ragweed genome size. We estimated a haploid genome size of 1.01 ± 0.0034 Gbp (range: 0.88–1.23 Gbp), a mean value consistent with previous reports (Kubešová *et al*., 2010; Bai *et al*., 2012; Battlay *et al*., 2023; Hrabovský *et al*., 2024), but with an expanded upper range exceeding prior sequencing (1.06 Gbp) and flow cytometry (1.13*–*1.16 Gbp) limits due to the inclusion of larger Australian genomes. In contrast to the relatively subtle differences between the European and North American ranges, Australian populations exhibit uniformly larger genomes relative to both regions, including their putative source genetic clusters (Fig. 3), pointing to a pronounced region‐specific expansion in genome size following introduction. These findings suggest that the relationship between genome size and invasion success is more context‐dependent than implied by broad interspecific patterns linking small genomes with invasiveness (Suda *et al*., 2015; Pyšek *et al*., 2018, 2023); our results align with a growing body of evidence indicating that genome size alone is not a consistent predictor of invasiveness. Similar findings have been reported for other successful invaders, such as certain *Acacia* species (Gallagher *et al*., 2011) and various Cactaceae species (Lopes *et al*., 2021), where no consistent change in genome size was observed in invasive populations. Instead, the adaptive significance of genome size is highly context‐specific and contingent upon the particular selective pressures of the introduced environment or the genomic architecture of adaptive traits, including the potential contribution of TE dynamics to standing genetic variation (Richardson & Pyšek, 2006; Potapenko *et al*., 2025). Notably, our quantile analysis provided little support for the lower‐boundary developmental constraint predicted by recent formulations of the LGC hypothesis (Bhadra *et al*., 2023; Bureš *et al*., 2024). Genome size showed no association with flowering time at lower quantiles, where developmental constraints should be the most apparent, and the only marginal signal was detected at the upper flowering time quantile in Australian populations. Nevertheless, Australian populations possess both the largest genomes and the latest flowering times observed across the species' range (van Boheemen *et al*., 2019), suggesting that genome size variation may still be associated with life‐history divergence. More broadly, while nucleotypic theory predicts developmental constraints associated with increased DNA content, it also predicts effects on cellular dimensions and physiological function (Šímová & Herben, 2012; Roddy *et al*., 2020), raising the possibility that genome size variation contributes to ecological divergence through multiple mechanisms.

### Evidence of adaptive genome size variation in common ragweed

**Fig. 6:**
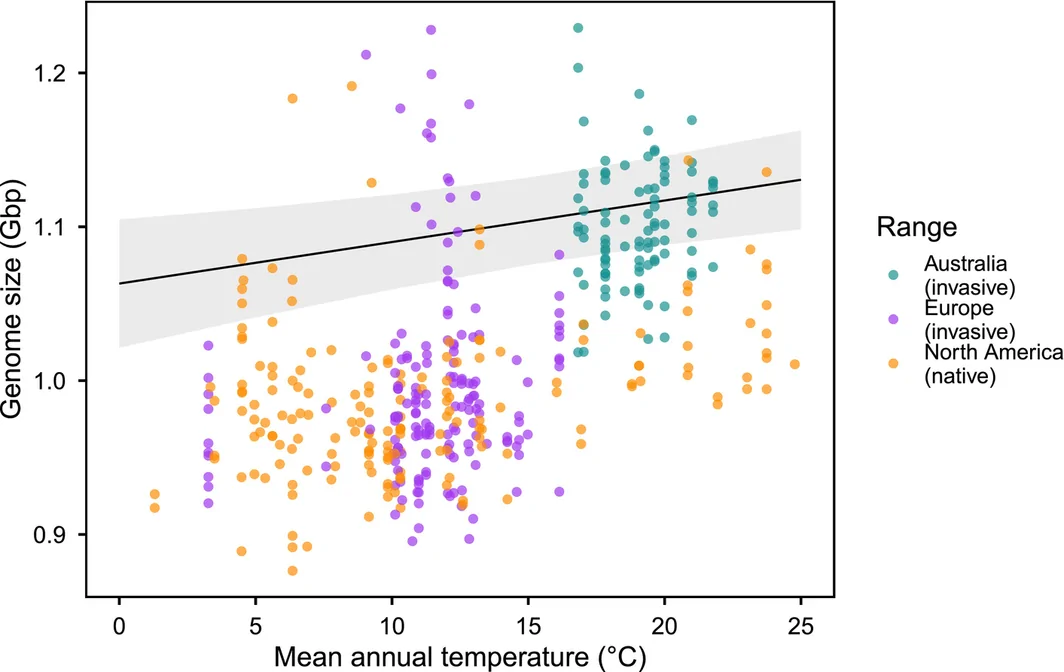
Relationship between genome size and mean annual temperature (MAT) across the native and invasive ranges of common ragweed (*Ambrosia artemisiifolia* L.). Each point represents the estimated genome size (Gbp) of an individual sample plotted against MAT (°C) at its sampling site. The black line represents the estimated linear relationship from a mixed‐effects model (model *R* ^2^ = 0.61, *P* = 0.0047), which included MAT, genetic principal components (PC1 and PC2) and geographical range as fixed effects, and population as a random effect. The grey shaded area indicates the 95% confidence interval around the regression line.

Across the native North American range, genome size shows greater among‐population differentiation than expected under neutrality (Fig. 5), consistent with divergent selection rather than neutral processes. Moreover, genome size correlates with MAT (Fig. 6) across ranges, consistent with climate‐mediated selection contributing to genome size differentiation. Environmentally structured genome‐size patterns occur in other systems including maize, where genome size correlates with altitude and temperature and tracks cell size and developmental rate (Díez *et al*., 2013; Tenaillon *et al*., 2016). In common ragweed, genome size shows climatic structuring with MAT across all ranges yet no significant association with flowering time in our common garden, indicating that genome size is unlikely to be a major determinant of this life‐history trait, in contrast to findings in some other species (e.g. Kreiner *et al*., 2023). Instead, the elevated *Q*_ST_ and *P*_ST_ values, together with significant *Q*_PC_ results (Notes S3), collectively indicate that selection has shaped genome size variation potentially through pathways beyond flowering time, such as other cellular, physiological or developmental pathways not captured in our common garden. Together, these results suggest that genome size could be responding to spatially variable selection while remaining largely decoupled from the key adaptive life‐history trait of flowering time. We note, however, that the lack of correlation between genome size and flowering time in our common garden experiment may partly reflect the benign, resource‐rich conditions under which plants were grown. Phenotypic correlations with genome size may disappear in benign experimental gardens but emerge under physiological stress or intense intraspecific competition typical of natural stands (Šmarda *et al*., 2010; Xiao *et al*., 2025; Li *et al*., 2026), suggesting that the adaptive significance of genome size variation may be underestimated in common garden settings.

Similar to North American populations, European common ragweed exhibits elevated *Q*_ST_ and *P*_ST_ relative to *F*_ST_, supporting range‐wide divergent selection on genome size. While *Q*_PC_ analysis provided weaker support for selection in Europe than in North America (Notes S13b), likely reflecting lower power or the distribution of adaptive divergence across multiple axes of genetic structure, both *Q*_ST_ *–F*_ST_ and *P*_ST_ *–F*_ST_ analyses consistently indicated that genome size differentiation exceeds neutral expectations. To our knowledge, neither *Q*_ST_ nor *P*_ST_ has been estimated for genome size in plants, so it remains unclear how often genome‐wide structural traits diverge from neutral expectations. *Q*_ST_ *–F*_ST_ comparisons for gene/genomic copy number variation in this species similarly revealed signatures of divergent selection on genomic variation in both Europe and North America (Wilson *et al*., 2025). Together, these results suggest that genome size differentiation has repeatedly evolved beyond neutral expectations across continents, although the relative importance of the underlying evolutionary drivers may vary among regions.

The Australian invasion some 100 yr ago relied on two distinct introductions: a main genetic cluster that has experienced a bottleneck (Putra *et al*., 2024; Battlay *et al*., 2025b), originating from high‐MAT sources in the southern and mideastern North American range, and a rarer genetic cluster with ancestry from eastern North America (Battlay *et al*., 2025b). Despite these different origins, both Australian clusters consistently exhibit larger genome sizes than their respective native source populations (Fig. S13). Within‐Australia *P*_ST_ fell below neutral expectations, while *Q*_ST_ was only marginally lower than expected under neutrality (Fig. 5; Notes S3b), consistent with a relatively homogeneous genome size increase across populations. The absence of a significant *Q*_PC_ signal in Australia (Notes S3c) is consistent with this pattern, suggesting no divergent selection within the range. Introduction bottlenecks likely reduced effective population size, facilitating an increase in TE copy number through relaxed purifying selection, as observed in other invasive species (Mérel *et al*., 2021), with TE remobilisation in novel environments potentially contributing further (Schrader *et al*., 2014). Although reduced efficacy of purifying selection following parallel bottlenecks could facilitate directional increases in TE load and genome size, such a mechanism alone would be expected to generate more heterogeneous outcomes among independently founded populations. The repeated increase in genome size across two independent introductions is therefore difficult to reconcile by drift alone, and is more consistent with colonisation filtering of large‐genome genotypes and/or parallel responses to shared environmental conditions following introduction (Prentis *et al*., 2008; Stapley *et al*., 2015; Suda *et al*., 2015; Cang *et al*., 2024). Pangenomic analyses of TE insertion age spectra, combined with expression profiling and reciprocal‐transplant assays, will help discriminate between demography‐driven relaxation of constraint and environment‐mediated selection.

### Conclusion

Genome size variation in common ragweed likely arises from the combined effects of demographic history, repetitive‐element dynamics and environmental context. Across North America and Europe, genome size differentiation is consistent with spatially varying selection, with climate‐associated differentiation evident across all ranges. By contrast, the uniformly larger genomes of Australian plants are potentially explained by demographic processes associated with invasion, likely shaped or reinforced by shared environmental conditions. Together, these findings reframe genome size from a static genomic property into a dynamic, evolutionarily labile trait that responds to demographic and ecological pressures accompanying range expansion. A key frontier now lies in resolving the mechanistic links between specific transposable element families, climate adaptation and fitness; particularly whether TE‐driven genome expansion represents a recurrent evolutionary response to novel environments, or a largely neutral outcome of founder dynamics that persists until purifying selection erodes it. Addressing these questions across multiple invasive taxa will be essential for understanding how genome architecture *–* shapes or is shaped by *–* the colonisation of new ranges.

## Competing interests

None declared.

## Author contributions

KAH, PB and BN conceptualised the study. BN and PB developed the methodology and software. Resources were provided by KAH, MDM, AF‐L and KGM, and data curation was performed by PB. BN conducted the formal analyses, with support from KAH, PB and JRS, and created the visualisations. BN drafted the manuscript, with review and editing by all authors. Supervision and project administration were provided by KAH, PB and JRS. Funding was acquired by KAH, MDM, JRS and AF‐L. All authors contributed and approved the final version of the manuscript.

## Disclaimer

The New Phytologist Foundation remains neutral with regard to jurisdictional claims in maps and in any institutional affiliations.

## Supporting information


**Fig. S1** Assessment of sequencing coverage bias on genome size estimates.
**Fig. S2** Relationships between estimated genome size and insert size, read length, callable depth, BUSCO‐filtered depth, and clonality.
**Fig. S3** Relationship between genome size estimates across read mapping quality thresholds.
**Fig. S4** Distribution of genome size estimated by the sequence‐based method.
**Fig. S5** Assessment of sequencing coverage bias on TE abundance estimates.
**Fig. S6** Identification of primary climatic predictors associated with common ragweed populations.
**Fig. S7** Estimation and comparison of genome size by flow cytometry using internal standards.
**Fig. S8** Distribution of haploid genome size estimated by flow cytometry.
**Fig. S9** Comparison between sequence‐ and flow cytometry‐based genome size estimates.
**Fig. S10** Comparison of TE and rRNA abundances between invasive ranges and native populations.
**Fig. S11** Proportion of TE and rRNA families across individuals from native and invasive ranges.
**Fig. S12** Abundance of different TE families across geographic ranges.
**Fig. S13** Comparison of genome size between Australian clusters and native populations.
**Notes S1** Quality controls addressing biases in genome size estimation from sequencing data.
**Notes S2** Genome size estimation by flow‐cytometry.
**Notes S3** Detail approaches for selection analysis of genome size (QST–FST, PST–FST, and QPC frameworks).
**Notes S4** Principal component analysis used to control for population structure.
**Notes S5** Polygenic value estimation for flowering time.
**Notes S6** Identification of the primary climatic predictor of genome size.
**Notes S7** Testing the Large Genome Constraint hypothesis.
**Table S1** Sampling information, sequencing coverage, and sequence‐based genome size estimates for 443 individuals.
**Table S2** Summary statistics of rRNA and TE annotations.
**Table S3** Descriptions of bioclimatic variables (BIO1–BIO19) from WorldClim.
**Table S4** Flow cytometry‐based genome size measurements for 40 common ragweed samples.
**Table S5** Genome size comparison between invasive ranges and native North American populations.
**Table S6** ANOVA and post hoc results for TE and rRNA abundance across ranges.
**Table S7** Linear mixed‐model effects of TE/rRNA family abundance on genome size.
**Table S8** Linear mixed‐model results for genome size with mean annual temperature, PC1, and PC2.
**Table S9** Linear quantile mixed model results for genome size across all ranges.
**Table S10** Linear quantile mixed model results for genome size within each range.
Please note: Wiley is not responsible for the content or functionality of any Supporting Information supplied by the authors. Any queries (other than missing material) should be directed to the *New Phytologist* Central Office.

## Data Availability

No new sequencing data were generated in this study. The whole‐genome resequencing data used were obtained from Bieker *et al*. (2022; doi: 10.1126/sciadv.abo5115) and Battlay *et al*. (2025b; doi: 10.1093/molbev/msae270). All accession nos. and source publications for the sequencing data used in this study are provided in Table S1. Flow cytometry genome size estimates generated in this study are provided in Table S4. All code used in this study is available at the following GitHub repository: https://github.com/Byonkesh/Ragweed_Genomesize_Evolution.
