# Supplementary material for: Selection shapes the evolution of genome size in a globally invasive plant
Source: New Phytol. 2026 Aug 28;252(2):875–91. doi: 10.1111/nph.71538 (PMC13583378; doi:10.1111/nph.71538)
Supplement: Supplementary file 1 [file NPH-252-875-s001.pdf]

New Phytologist Supporting Information

Article title: Selection shapes the evolution of genome size in a globally invasive plant

Authors: Byonkesh Nongthongbam[1]*, Paul Battlay[1], Katherine G. Maunder[2], Alexandre Fournier-Level[3], Michael D. Martin[4], John R. Stinchcombe[2], Kathryn A. Hodgins[1]

Article acceptance date: 29th July 2026

A) Supplementary notes:

Notes S1: *Quality controls addressing biases in genome size estimation from sequencing data*

We systematically evaluated potential technical sources of error that could influence our results because of the known biases associated with sequencing-based genome size estimation (Poptsova et al, 2014; van Heesch et al, 2013). Given the relatively low average sequencing depth (~7.1×) across our samples, we first assessed whether coverage depth introduced systematic bias into our genome size and TE abundance estimates. We regressed whole-genome average depth against estimated genome size and against the abundance of each TE family. Genome size showed no meaningful association with coverage (R^2 = 0.00028; Figs S1), confirming that low-coverage samples did not systematically under- or overestimate genome size. Similarly, the vast majority of TE families showed negligible correlations with coverage (R^2 < 0.05; Figs S5), with the exception of rRNA repeat families (18S, 28S, 8S), which showed moderate associations (R^2 = 0.10-0.23; Figs S5). These rRNA patterns likely reflect genuine biological variation in rRNA copy number rather than technical bias, as rRNA genes are known to vary considerably in copy number across individuals (Parks et al, 2018; Sims et al, 2021; Sultanov et al, 2022). Together, these results indicate that sequencing depth did not systematically bias our genome size or TE abundance estimates

Having established that coverage depth was not a confounding factor, we next assessed a broader set of quality metrics related to read integrity, coverage uniformity, and sequence-dependent fragmentation, all of which are known to introduce bias in depth-based genome size inference. We first examined read insert size (representing the distance between paired-end reads), length and mapping quality as potential indicators of DNA fragmentation and alignment confidence, respectively (Li et al, 2008). Average insert size was calculated using the `IS` option of `samtools stats` in samtools v.1.16.1 (Li et al, 2009). Our analysis revealed no significant linear correlation with average insert size and genome size (R^2 = 0.011; Figs S2a). Average read length was calculated using the `RL` option of `samtools stats` and showed no clear linear correlation with genome size (R^2 = 0; Figs S2b), indicating that sequencing-induced DNA fragmentation did not bias our estimates. Furthermore, applying various mapping quality thresholds (MAPQ ≥ 2, 5, 7, 10, and 15) and recalculating genome size estimates using `samtools depth -Q` demonstrated consistent and strong relationship with genome size estimated

without filters across all thresholds (Figs S3). Thus, alignment confidence did not significantly affect our genome size estimations.

We next assessed the uniformity of genome-wide coverage and the impact of PCR duplication. Coverage depth was evaluated at callable loci, defined as genomic positions with ≥10× read coverage and sufficient quality for reliable consensus sequence generation (Ajay et al, 2011; Marshall et al, 2020), as established by Bieker et al. (2022). By calculating depth at these loci (`samtools depth -b callable_depth.bed`) and comparing it to whole-genome sequencing depth, we observed a strong linear relationship ($R^2 = 0.99$; Figs S2c), suggesting even coverage across the genome, and validating the use of whole-genome depth for genome size estimation. Additionally, we examined BUSCO-based scaling, which was used to normalize genome-wide sequencing depth by the average depth across conserved, single-copy BUSCO genes. To minimize potential biases from structural variation or inconsistent coverage, we computed depth variance at each BUSCO gene site across all samples using `samtools depth`. Only single-copy BUSCO genes with depth variance within the first standard deviation were retained, effectively excluding regions prone to structural variation such as copy number variations (adapted from Gilbert, 2024). Genome size for each sample was then estimated based on these filtered BUSCO site depths. Comparing these BUSCO-filtered genome size estimates to unfiltered estimates revealed a significant relationship, confirming that our scaling approach did not introduce systematic bias ($R^2 = 0.97$; Figs S2d). Finally, we evaluated the effect of clonality (PCR duplicates) on our results. Since our standard pipeline removes duplicates, we used unfiltered aligned reads to calculate clonality (identified via `samtools flagstat`) and re-estimate genome size. We found only a weak correlation between clonality and genome size estimates including duplicates ($R^2 = 0.069$; Figs S2e) suggesting PCR artifacts did not bias the data. Additionally, the geographic patterns of genome size remained consistent when using estimates that included duplicates (Australia > Europe > North America; Figs S2f), also indicating the lack of clonality effect on our estimates. Together, these quality assessments demonstrate that our genome size estimates are robust against known technical and biological sources of sequencing bias. These controls provide confidence that observed geographic patterns in genome size do not reflect methodological artifacts.

Although short-read-based genome size estimates can be sensitive to coverage and reference choice, multiple independent validation approaches; including flow cytometry confirming that Australian genomes are larger than those from North America and Europe, and sensitivity analyses spanning insert size, read length, mapping quality, callable base depth, clonality, and BUSCO gene depth variance, strongly indicate that the observed range differences and genome size–climate associations reflect genuine biological signal rather than methodological artefacts.

Notes S2: *Genome size estimation by flow-cytometry*

To independently validate genome size estimates obtained from the resequencing approach, we performed flow cytometry. Although different individuals were used due to limited tissue availability, all

samples originated from the same populations as the sequenced individuals. Sample sizes for flow cytometry per range were determined using power analyses based on effect sizes estimated from sequencing-based genome size differences among ranges, ensuring sufficient power to detect the observed genome size pattern.

Genome size estimation followed established protocols (Sliwinska et al, 2022; Hrabovský et al, 2024). Plants were grown in a common garden at Koffler Science Reserve in Ontario, Canada. Leaf samples were collected in late September 2024, dried with silica gel powder and stored at room temperature until analysis. In total, 40 samples were used (exceeding the minimum sample size per ranges required based on power analyses): 16 from North America, 6 from Europe, and 18 from Australia (Tables S4). Nuclear suspensions were prepared by co-chopping 60–70 mg of dried common ragweed leaf tissue with 60–70 mg of *Manihot esculenta* (cassava-red stem variety) leaves as an internal control. Cassava was used as internal reference standard for common ragweed because its genome size (~600–772 Mbp/C based on assembly estimates; Bredeson et al, 2016; Awoleye et al, 1994) is smaller yet sufficiently close to the common ragweed genome (~1.02–1.11 Gbp/C; Kubešová et al, 2010; Bai et al, 2012; Battlay et al, 2023) to ensure non-overlapping but adjacent fluorescence peaks during flow cytometry, making it a favourable control. We first estimated the genome size of the cassava individual used, by calibrating it against a standard control, *Solanum lycopersicum* L. (variety: 'Stupické polní rané' (2C = 1.96 pg; Doležel et al. 1992). The calibrated cassava value (1.45 ± 0.01 Gbp/2C) was then used to normalise common ragweed genome size calculation. The homogenate was filtered through 2–3 layers of Miracloth to remove debris. Then, 5 µL of 1 mg/mL RNase was added into the suspensions and incubated at 37℃ for 30 minutes. DNA was then stained with 50 µL of propidium iodide (PI) (1 mg/mL), and fluorescence signals (standard 10,000 nuclei populations) were measured within an hour. For each sample, measurements were also taken from unstained aliquots to assess and correct for background fluorescence. Flow cytometry was performed on a FACSymphony A3 analyzer (BD Biosciences, USA) at FlowCore, Monash University and the data were analysed using FlowJo software (FlowJo LLC, USA). The genome size (2C) was calculated based on the mean fluorescence intensity of PI-stained nuclei, normalized to the cassava reference genome. For comparison with sequence-based estimates, the 2C value (in picograms) was converted to haploid genome size (1C) by dividing by two and then converted to megabase pairs (Mbp) using 1 pg = 978 Mbp. Due to the large number of samples, analyses were performed in two batches. To assess instrument variability (run-to-run consistency), multiple technical replicates were measured for each sample. To further evaluate variability introduced by sample preparation (extraction variability) and potential batch effects, five samples from the first batch were re-extracted from their original dried tissue and re-analyzed on the second day. Adhering to standard thresholds (Greilhuber et al, 2007), G1 peak CVs were <5% and batch-to-batch estimate CVs for genome size estimates ranged from 0.11-1.98%, confirming high technical reproducibility without systematic extraction or batch effects.

Notes S3: *Detail approaches for selection analysis of genome size*

S3a: Genomic relatedness matrix construction, heritability and Q_{ST} estimation

To estimate Q_{ST}, we constructed a genomic relatedness matrix (GRM) from the aforementioned VCF and implemented an animal model to estimate genetic variances in genome size. To ensure the GRM reflected genome-wide additive relatedness without bias from linked regions or rare variants, we applied the following filters: we excluded SNPs located within genes and known haploblocks (regions of high LD), filtered for a Minor Allele Frequency (MAF) ≥ 0.01, and performed LD-pruning to remove SNPs with an r^2 = 0.5 within a 50-kb window, using PLINK v1.9 (Purcell et al, 2007; https://github.com/chrchang/plink-ng). Following these quality and linkage filters, we randomly subsampled the remaining SNPs to a final dataset of 50,000 SNPs for GRM construction. The GRM was constructed using the VanRaden (2008) method in the R package `AGHmatrix` (Amadeu et al, 2023). To ensure mathematical stability for the animal model, the matrix was made positive-definite using the heuristic bending method in the R package `mbend` (Nilforooshan, 2020), adding a small positive value (1×10^{-6}) to any eigenvalue smaller than 1×10^{-6}. We then modelled genome size using a Bayesian animal model in `MCMCglmm` (Hadfield, 2010) with the GRM precision matrix supplied via the `ginverse` argument, fitting genome size as a function of a grand mean intercept and an individual-level random effect whose covariance structure was informed by genome-wide SNP relatedness. A parameter-expanded prior was used for variance components, and the chain was run for 7,010,000 iterations with a burn-in of 10,000 and a thinning interval of 7,000, yielding 1,000 posterior samples. Autocorrelations between MCMC samples were below the recommended level of 0.1, yielding high effective sample sizes (mostly ≥ 1,000, minimum = 549) for all estimates. We inspected plots of traces and posterior distributions to ensure that models converged. Other priors were explored and gave similar results to those presented here. Then, heritability was estimated for each of the 1,000 MCMC iterations as:

$$h^2 = \frac{V_A}{V_A + V_R}$$

, where V_A is the posterior additive genetic variance (animal component) and V_R is the residual variance (units' component) extracted from the VCV matrix of the fitted MCMCglmm object (Falconer & Mackay, 1996; Lynch & Walsh, 1998; de Villemereuil, 2018; Perrier C et al, 2018). Individual genomic breeding values were extracted directly from the posterior distribution of random effects stored in the Sol matrix of the fitted MCMCglmm object (Hadfield, 2010; Wilson et al, 2010). For each of the 1,000 retained posterior samples, the full vector of individual breeding values was extracted and used to calculate Q_{ST} directly, without collapsing to a single point estimate. Within each geographical range (North America, Europe, Australia), breeding values were partitioned by population and Q_{ST} was calculated as (Spitze, 1993; Lande, 1992; Whitlock, 2008; 2009):

$$Q_{ST} = \frac{\sigma^2_{GB}}{\sigma^2_{GB} + 2 \times \sigma^2_{GW})}$$

, where σ^2_{GB} is the variance of population mean breeding values, used as an estimate of among-population additive genetic variance and σ^2_{GW} the mean within-population variance of breeding values, estimating the within-population additive genetic variance across populations in that range. Applying this calculation across all 1,000 MCMC iterations produced a posterior distribution of Q_{ST} for each range,

from which we report the posterior mean and 95% credible interval (2.5th and 97.5th percentiles). Our approach propagates the uncertainty of breeding value estimates sampled from the posterior distribution through to the final Q_{ST} estimate and is more rigorous than approaches based on a single point estimate of heritability, as recommended by Leinonen et al. (2013) and Whitlock (2008).

S3b: P_{ST}-F_{ST} comparisons for genome size differentiation

To complement the Q_{ST}-F_{ST} analysis, we implemented a P_{ST}-F_{ST} comparison as an additional line of evidence for divergent selection on genome size (Spitze, 1993; Prout et al, 1993) and calculated P_{ST} for genome size separately for populations within Australia, Europe, and North America. Following the approach by Sæther et al. (2007) and Whitlock (2008), we estimated P_{ST} as

$$P_{ST} = \frac{(VP, among)}{(VP, among)\ +\ 2(VP, within)}$$

where *VP, among* is the among-population variance and *VP, within* is the within-population variance for the trait. Variance components were estimated using linear-mixed models implemented in R\ lme4 (Bates et al, 2015), by fitting the trait (genome size) as the response and population as a random intercept. Genome size was measured from individuals grown in a common-garden experiment, thereby minimising environmental sources of variation. Heritability of genome size was estimated at 0.886 (95% CI: 0.772–0.953) from the Bayesian animal model described above, confirming that the vast majority of phenotypic variance reflects additive genetic differences among individuals. Consequently, P_{ST} is expected to closely approximate Q_{ST} (Whitlock, 2008; Brommer, 2011), and any residual non-additive transmission processes would bias P_{ST} downward, making our estimates conservative with respect to detecting genetic differentiation.

To assess whether lower within-population sample sizes could inflate P_{ST} estimates, we generated 2,000 permuted datasets by randomly assigning individuals to populations while preserving overall sample sizes. P_{ST} values from observed data were then compared against this null distribution. A P_{ST} value exceeding the 99th percentile of the neutral F_{ST} distribution was interpreted as evidence of trait divergence exceeding neutral expectations, consistent with divergent selection acting on the trait. Following Sun et al. (2020) and Brommer (2011), we also performed P_{ST} sensitivity across a range of c values (0.1–1.5), where c represents the proportion of total additive genetic variance attributable to among-population differences and heritability values (h^2 = 0.25, 0.50, 0.75).

Result:

In concordance with Q_{ST}-F_{ST} analysis, P_{ST}-F_{ST} analysis revealed that genome size differentiation significantly exceeded neutral expectations in North America (P_{ST} = 0.363 > F_{ST} threshold = 0.257) and Europe, P_{ST} = 0.454 > F_{ST} threshold = 0.277) (Notes S3b Fig. 1), providing strong evidence that divergent selection is driving genome size differences among populations in these ranges. Sensitivity analysis further confirmed the robustness of the selection signatures in North America and Europe, as P_{ST} remained significantly higher than the neutral F_{ST} threshold across all tested h^2 values at biologically

realistic c values (Notes S3b Fig. 2). In contrast, populations in Australia exhibited almost no differentiation in genome size. The observed P_{ST} was 0, which fell well below the neutral F_{ST} thresholds, consistent with an absence of divergent selection or the influence of stabilizing selection within this range.

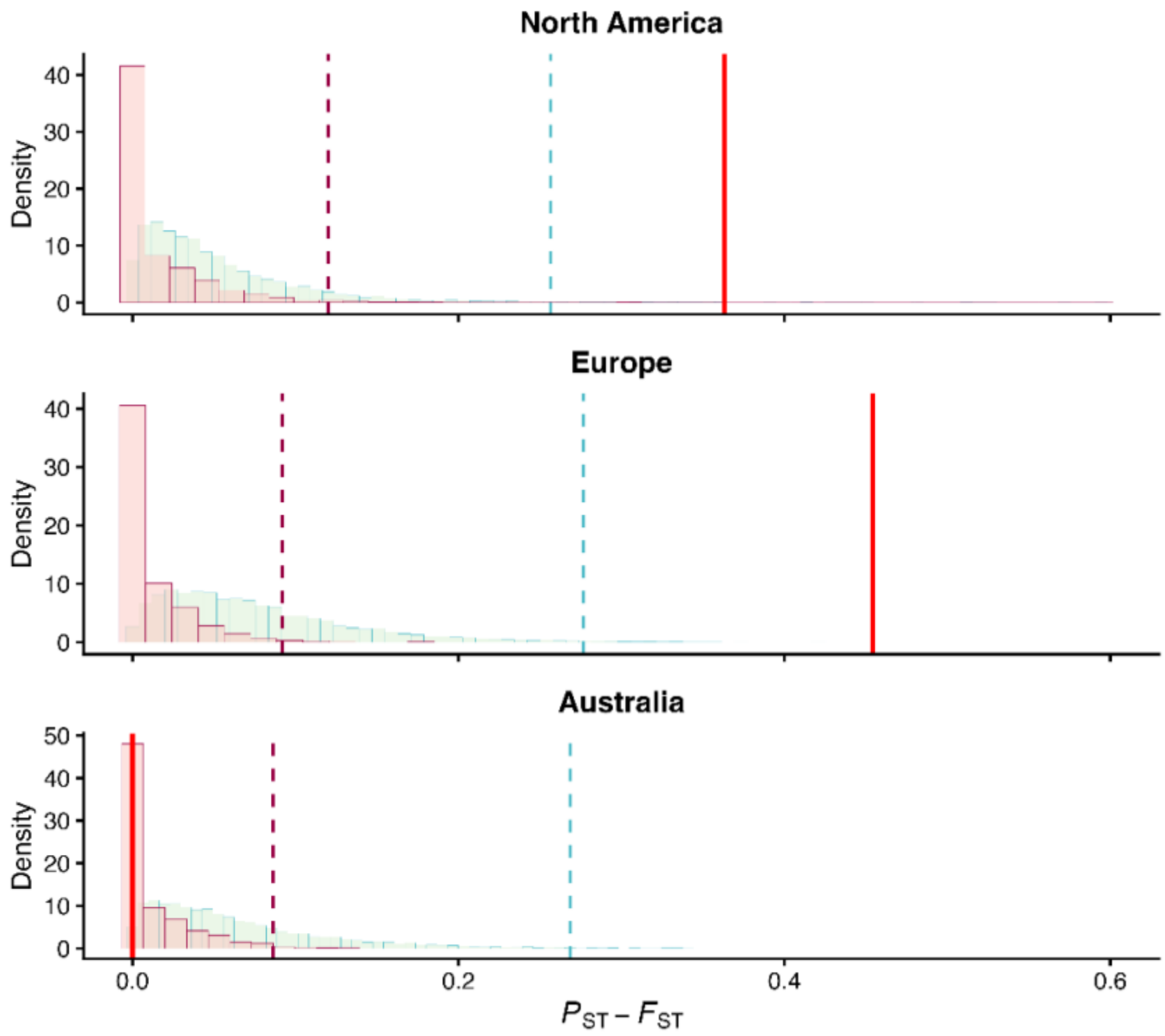

Notes S3b Fig. 1: Phenotypic differentiation (P_{ST}) compared to neutral genetic differentiation (F_{ST}) for North America, Europe, and Australia. The light green background histograms represent the null distribution of 10,000 putatively neutral F_{ST} markers, with the teal dashed line indicating the 99th percentile of this distribution. Pink histograms represent the null distribution generated from 2,000 permutations. The vertical dashed pink line indicates the 99th percentile of the permutation null, while the solid red line indicates the observed P_{ST} value.

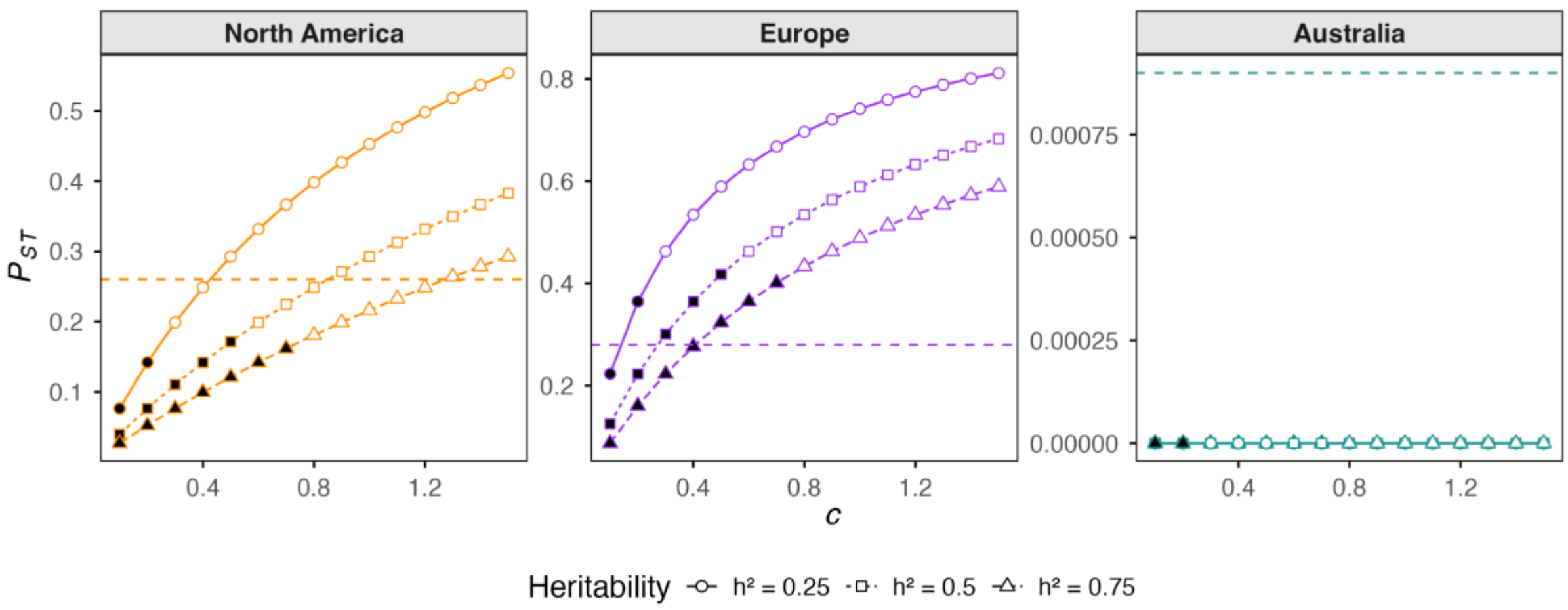

Notes S3b Fig. 2: P_{ST}–F_{ST} sensitivity analysis across ranges. P_{ST} values are plotted across a range of c values for three heritability assumptions (h^2 = 0.25, 0.5, 0.75). The horizontal dashed line indicates the F_{ST} threshold (99th percentile for North America and Europe; 1st percentile for Australia). Filled symbols indicate biologically informative parameter combinations where c ≤ h^2; open symbols indicate uninformative combinations where c > h^2. Filled symbols exceeding the dashed line indicate $P_{ST} > F_{ST}$ under conservative assumptions, consistent with divergent selection; filled symbols below the threshold indicate differentiation below neutral expectations.

S3c: Q_{PC} framework for testing divergent selection of genome size

To complement the P_{ST}–F_{ST} and Q_{ST}–F_{ST} comparisons, we additionally applied the Q_{PC} framework (Josephs et al, 2019) to test for divergent selection on genome size while explicitly accounting for genome-wide population structure. Unlike P_{ST}–F_{ST} and Q_{ST}–F_{ST} comparisons, which treat populations as discrete units, Q_{PC} projects the phenotype onto the eigenvectors of the kinship matrix to identify axes of population structure along which trait divergence exceeds neutral expectations. Following Josephs et al. (2019), a kinship matrix (K) was constructed from the same 50,000 randomly sampled SNPs used for the Q_{ST} analysis, after excluding sites with missing data, using the VanRaden (2008) method implemented in the R/AGHmatrix applied to 289 individuals. Unlike the GRM used in the Bayesian animal model, the kinship matrix was not subjected to positive-definiteness correction, preserving the full eigenspectrum required for Q_{PC}. The K matrix was eigendecomposed to obtain principal components (PCs) and their associated eigenvalues. The PC cutoff (pcmax = 52) was defined as the first PC at which cumulative variance explained exceeded 30% of total genetic variance, following Josephs et al. (2019) who used the first PCs cumulatively explaining 30% of variation in K.

For each invasion range independently (North America, Europe, and Australia), genome size was mean-centred and projected onto the eigenvectors of K. For each of the top 52 PCs, the Q_m statistic was computed as:

$$Q_m = \frac{(Z.u_m)^2/\lambda_m}{\frac{1}{144}\sum_{i=146}^{289}(Z.u_i)^2/\lambda_i}$$

Where Z is the mean-centred vector of genome size values for individuals within a given range, u_m is the mth eigenvector of K, and λ_m is the corresponding eigenvalue. The numerator captures among-population divergence in genome size along PC_m (σ^b), and the denominator estimates neutral within-population variance (σ^w) from the bottom 144 PCs ($\frac{n}{2}$, where n = 289, following Josephs et al, 2019). Under neutrality, Q_m follows an F distribution with 1 and 144 degrees of freedom, from which p-values were derived. A significant positive Q_m indicates that trait divergence along a given PC axis exceeds neutral expectations. For display purposes, 95% confidence intervals were plotted as lines with slope

$\pm 1.96\sqrt{(V_a \times \lambda_m)}$ per PC_m, where V_a is the neutral additive variance estimated from the bottom 144 PCs, following Josephs et al. (2019). The significance threshold applied was $p < 0.05$.

Result:

Q_{PC} analysis provided convergent evidence with the P_{ST}–F_{ST} and Q_{ST}–F_{ST} results, confirming strong directional selection on genome size in North America, weak signal in Europe, and no signal in Australia (Notes S3c Fig. 1).

In North America, 15 out of 52 tested PCs showed significant associations between genome size and PC score ($P < 0.05$), with the strongest signal at PC12 ($Q_m = 20.28$, $P = 1.37\times10^{-5}$; Notes S3c Fig. 2). Across all significant PCs, the observed regression of genome size on PC score consistently and substantially exceeded the neutral 95% confidence interval (Notes S3c Fig. 2), indicating that genome size divergence among North American populations is far greater than expected under genetic drift alone. In Europe, three PCs reached the significant threshold (PC17, PC22, PC45; $P < 0.05$), with observed projections marginally exceeding the neutral confidence interval. In Australia, no PCs showed significance (all $P > 0.12$), with observed projections falling entirely within the neutral confidence interval (Notes S3c Fig. 2), indicating no detectable divergent selection on genome size.

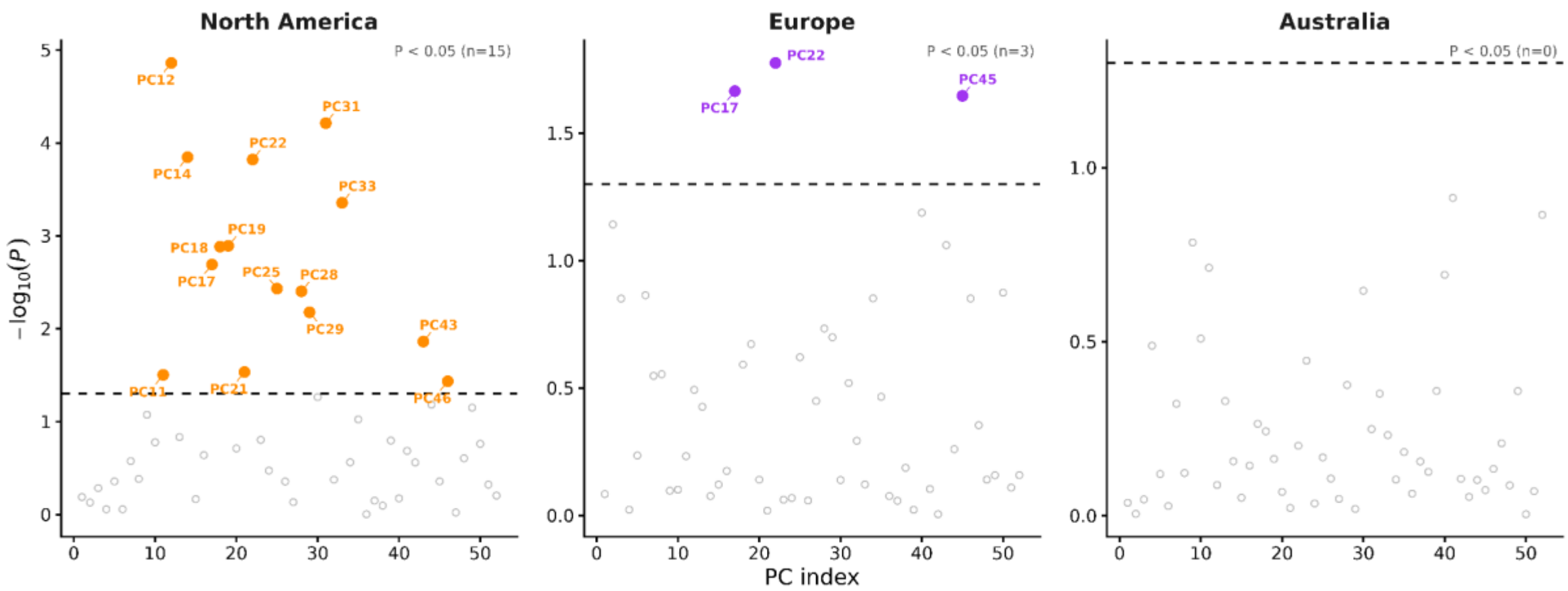

Notes S3c Fig. 1: Q_{PC} projections of genome size onto principal component axes of the kinship matrix across geographical ranges. The y-axis shows $-\log_{10}$(P-value); the dashed horizontal line marks the significance threshold ($P = 0.05$). Points above the threshold ($P < 0.05$) are shown in range-specific colour; non-significant points are shown in grey, with PC labels shown for significant points. North America: 15 significant PCs. Europe: 3 significant PCs. Australia: no significant PCs.

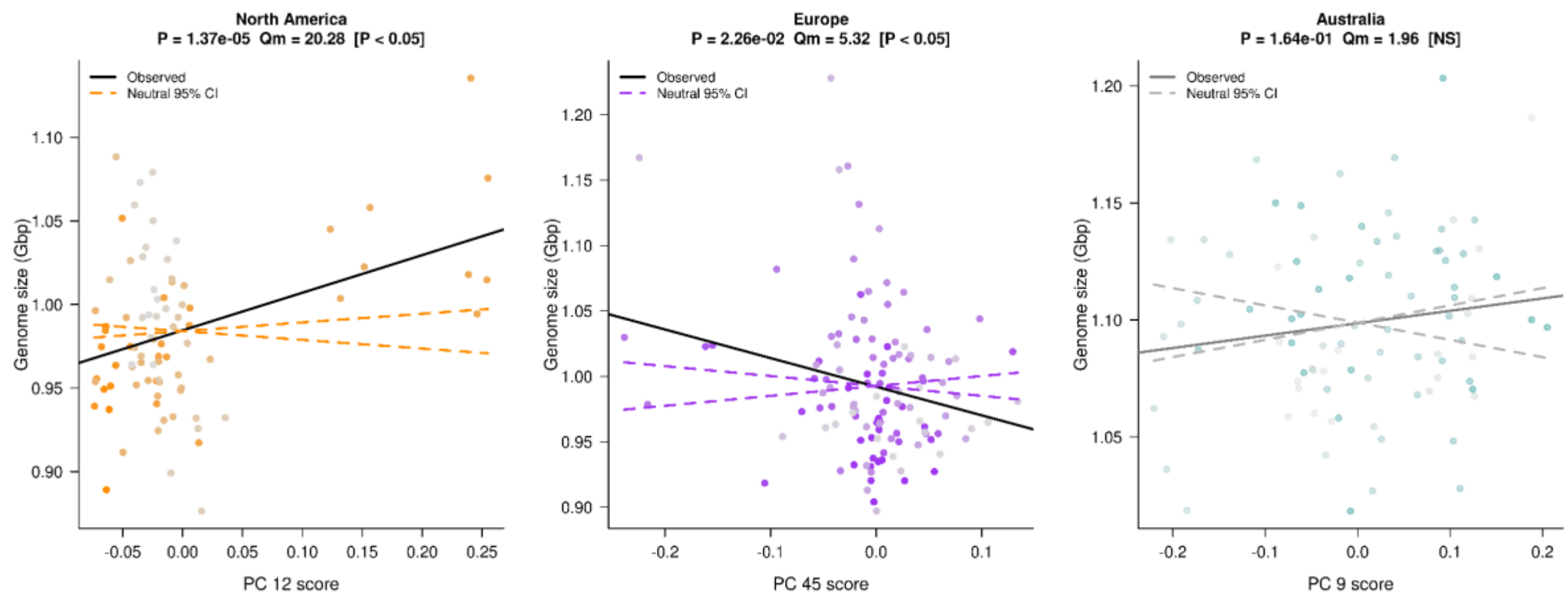

Notes S3c Fig. 2: Q_{PC} projections of genome size onto one PC axis per range. Genome size (Gbp) is plotted against individual scores on the PC axis within each range: North America (PC_{12}, P = 1.37×10^{-5}), Europe (PC_{45}, P = 0.023), and Australia (PC_{9}, P = 0.164). The solid line shows the observed regression of genome size on PC score. Dashed lines indicate the expected regression slope under neutrality, derived from drift-based variance estimates ($\pm 1.96\sqrt{(Va \times \lambda m)}$); slopes falling outside this envelope indicate divergence beyond neutral expectations. In North America and Europe, the observed regression exceeds the neutral envelope, consistent with divergent selection on genome size. In Australia, the observed regression falls within the neutral envelope, indicating genome size variation consistent with neutral genetic drift.

Notes S4: *Principal Component Analysis*

To control for the effect of population structure on the analyses of genome size and life history traits, we performed principal component analysis (PCA) on samples. Analyses were based on genome-wide SNPs previously identified and validated in Battlay et al. (2025). Separate PCAs were conducted for the overall analysis (439 samples) and the phenotypic analysis (221 samples). Genotype likelihoods were calculated from BAM files using ANGSD v0.939 (Korneliussen et al, 2014; https://github.com/ANGSD/angsd). Quality filters included a minimum mapping quality of Q30, minimum base quality of Q20, and a minimum minor allele frequency of 0.05, similar to parameters detailed in Battlay et al. (2025). We modified the site inclusion criterion to require a minimum of 330 individuals with data (`-minInd 330;` 75 % of total 440 samples). Variant sites were then pruned for independence using PLINK v1.9 (Purcell et al, 2007; https://github.com/chrchang/plink-ng/blob/master/1.9/README.md), using a window size of 50 kbp, a step size of 5 SNPs, and an r^2 threshold of 0.5 (`-indep-pairwise 50 5 0.5`). Sites were further filtered to include only those that were both independent and outside annotated genes or haploblocks, before being randomly downsampled to 100,000 for computational efficiency. A covariance matrix was then generated from

these filtered sites with PCangsd v1.2 (Meisner and Albrechtsen, 2018). The resulting PC scores were included as fixed effects in linear mixed models to account for the effect of population structure.

Notes S5: *Polygenic value estimation for flowering time*

Using flowering onset GWAS results from Battlay et al. (2025a), LD clumping was performed in PLINK to identify a single SNP per GWAS peak in each LD block. We considered only SNPs with significant association (Bonferroni-corrected P < 0.05), and 'clumped' candidate SNPs in plink v. 1.9 to remove SNPs in LD (r^2 > 0.3) with the top candidate in each 1 Mbp window. Following clumping, 380 of 513 associated SNPs were retained. We then inferred genotypes for each of the 380 sites across our samples using ANGSD. We estimated the genotype likelihoods using the GATK model (`-GL 2`), with major and minor allele defined from the candidate SNPs list (`-doMajorMinor 4`) for each site. Reads with mapping quality below Q_{30} were removed (`-minMapQ 30`), only bases with quality scores above Q_{20} were retained (`-minQ 20`), and a minor allele frequency filter of 0.05 was applied (`-minMaf 0.05`). Allele frequencies were estimated assuming a known major allele, with the minor allele frequency inferred from the data (`-doMaf 2`) and only sites with a probability of being variable less than 1×10^{-6} were retained (`-SNP_pval 1e-6`). We then used genotype data along with their effect sizes from the GWAS, to estimate the polygenic value for flowering onset (PGV_{FT}) of each genotyped individual as described in Kreiner et al. (2023):

$$PGV_{FT} = 2\sum_{i=1}^{L} \alpha p$$

where L represents the set of 380 LD-pruned loci with bonferroni-corrected P < 0.05, α is the effect size of the minor allele at locus i derived from the GWAS, and p is the individual's genotype frequency at that locus (coded as 0 for homozygous reference, 0.5 for heterozygotes, and 1 for homozygous alternates).

Notes S6: *Identification of primary climatic predictor*

To identify informative environmental predictors of genome size, we first extracted 19 bioclimatic variables (BIO1–BIO19, Tables S3) from WorldClim (Fick & Hijmans, 2017) for each sampling location using geographic coordinates (latitude and longitude) and year of sample collection. We assessed the potential for redundancy by calculating pairwise Pearson correlation coefficients across all 19 variables (Figs S6,a). Highly correlated variables (absolute Pearson's r > 0.7) were identified and manually reduced by retaining a single representative variable from each correlated cluster. From the resulting uncorrelated clusters, five variables were retained: BIO1 (Mean Annual Temperature, MAT) representing the temperature-related cluster (BIO3–BIO7, BIO9–BIO11); BIO2 (Mean Diurnal Range); BIO8 (Mean Temperature of Wettest Quarter); BIO12 (Annual Precipitation) representing the precipitation-related cluster (BIO13, BIO14, BIO16–BIO19); and BIO15 (Precipitation Seasonality). Then, PCA was subsequently performed on these five variables to quantify the environmental variance explained by each component (Figs S6b). The first principal component explained 48% of the total variance and was strongly correlated with MAT (r = 0.84) (Figs S6,b,c), which has established biological

importance for common ragweed. Accordingly, MAT was selected as the primary climatic predictor for subsequent analyses.

Notes S7: *Testing the Large Genome Constraint hypothesis*

To test whether genome size constrains developmental speed consistent with the LGC hypothesis, we first fitted a linear mixed model of flowering time on genome size using the `lme4` R package (Bates et al, 2015), controlling for range, polygenic value for flowering time (PGV_{FT}), PC1, PC2, and population as a random intercept. This revealed no significant effect of genome size on mean flowering time. Because the LGC hypothesis specifically predicts a lower boundary constraint; larger genomes restrict the minimum time required for development, rather than shifting the mean. Thus, standard mean-based regression is not well suited to detect this signal. We therefore fitted Linear Quantile Mixed Models (LQMM) using the `lqmm` R package (Geraci, 2014) at five quantiles (τ = 0.10, 0.25, 0.50, 0.75, and 0.90), using data from 221 individuals (van Boheemen et al, 2019). The lower quantiles (τ = 0.10, 0.25) were of primary interest as they captured the minimum developmental boundary predicted by the LGC hypothesis, while higher quantiles served as comparators to assess whether any effect was specific to the lower tail of the distribution. The fitted R models are:

Across all ranges,

Phenotypic trait (flowering time) ~ Genome size + Range + PGV_{FT} + PC1 + PC2 + (1 | Population)

Within each range,

Phenotypic trait (flowering time) ~ Genome size + PGV_{FT} + PC1 + PC2 + (1 | Population)

where Genome size, PGV_{FT}, PC1 and PC2 were treated as fixed effects as previously described, and Population as a random intercept. This framework allowed us to isolate the effect of genome size on developmental thresholds while controlling for the additive genetic component of flowering time and population structure.

B) Supplementary Figures:

Figures S1: Assessment of sequencing coverage bias on genome size estimates in common ragweed (*Ambrosia artemisiifolia* L.). Scatter plot showing the relationship between sequencing coverage (mean depth, ×) and sequence-based genome size estimates (Gbp) across all sampled individuals, testing whether coverage systematically biases genome size estimation. Each point represents one individual.

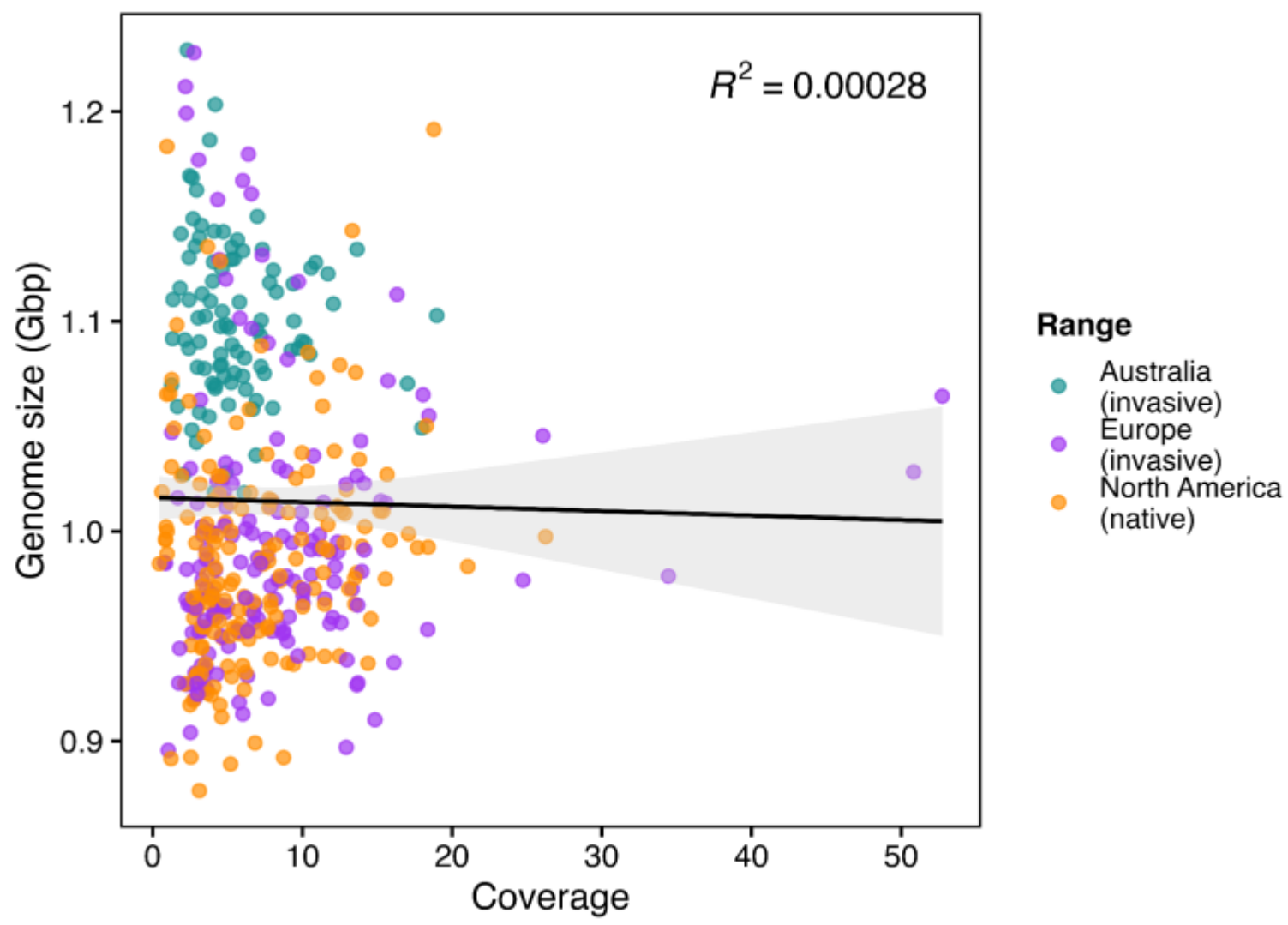

Figures S2: Relationships between estimated genome size and various sequencing metrics. Scatter plots depict the relationship between: (a) estimated genome size (Gbp) versus sequencing insert size (bp); (b) estimated genome size versus sequencing read length (bp); (c) callable read depth versus average whole-genome read depth; (d) estimated genome size versus genome size calculated using the depth of variance-filtered BUSCO (Benchmarking Universal Single-Copy Orthologs) sites; (e) genome size estimated with duplicates versus clonality. In (a-e), each point represents one individual; the dark blue line shows the linear regression fit, with the light blue shaded area indicating the 95% confidence interval. (f) Genome size box plot estimated from sequence data including duplicate reads, to check the consistency of the pattern with estimates without duplicates (Fig. 2B). Boxes show the interquartile range (IQR, 25th-75th percentile) of the raw data, with whiskers extending to 1.5 × IQR and individual values overlaid as points; the horizontal line within each box marks the estimated marginal mean from a linear mixed-effects model with geographic range, genetic principal components (PC1 and PC2) as a fixed effect and population as a random intercept. Above the boxes, we provide sample size (n), and statistical values (model's estimated marginal mean ± standard error). Asterisks denote statistical significance from pairwise comparisons (***P < 0.001, **P < 0.01, *P < 0.05, ns = not significant).

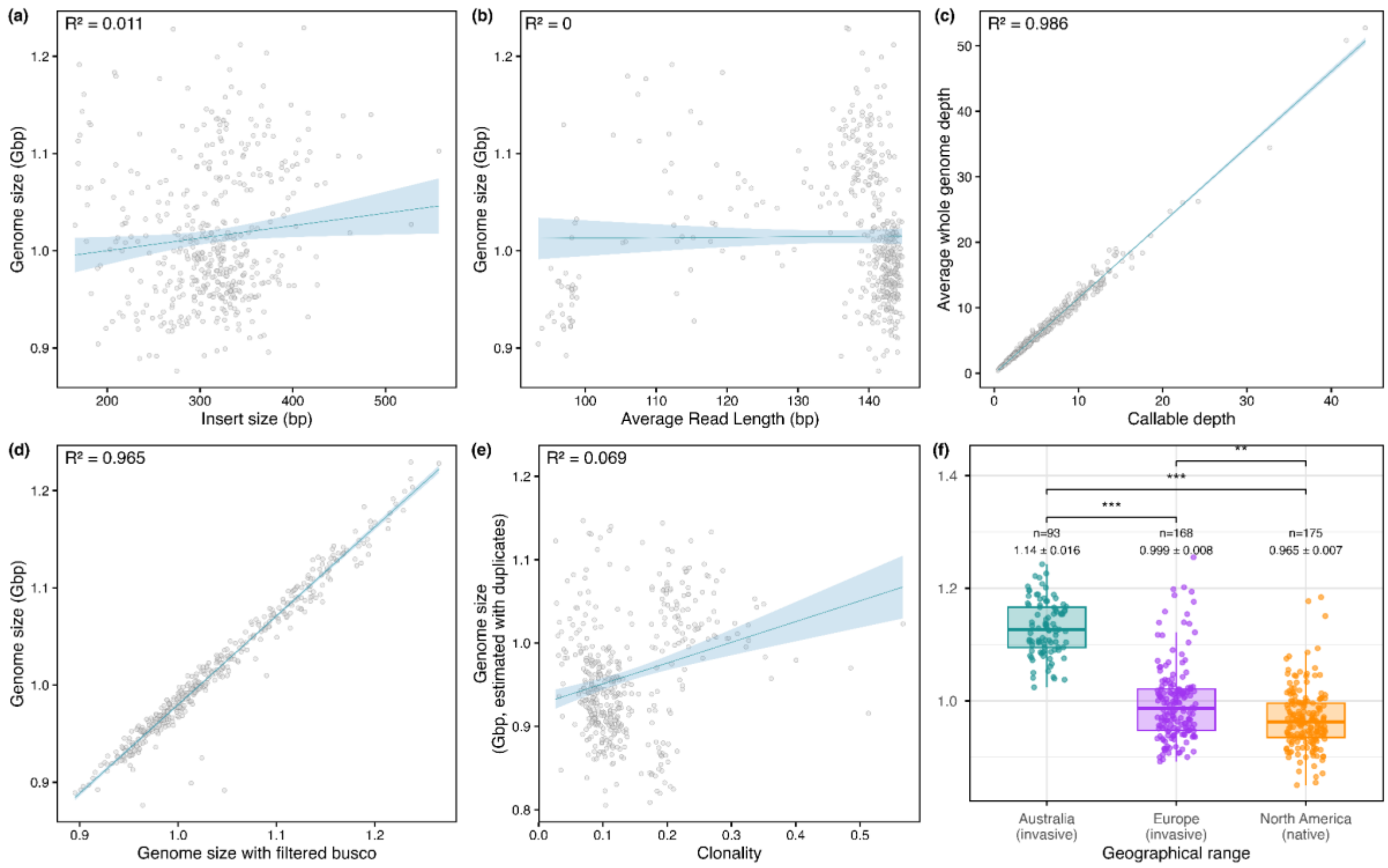

Figures S3: Effect of read-mapping quality filtering on genome size estimates in common ragweed (*Ambrosia artemisiifolia* L.). Scatter plot showing the relationship between genome size (Gbp) estimated using reads filtered at increasing mapping-quality thresholds (Q2, Q5, Q7, Q10, Q15) and genome size estimated without quality filtering, across all sampled individuals.

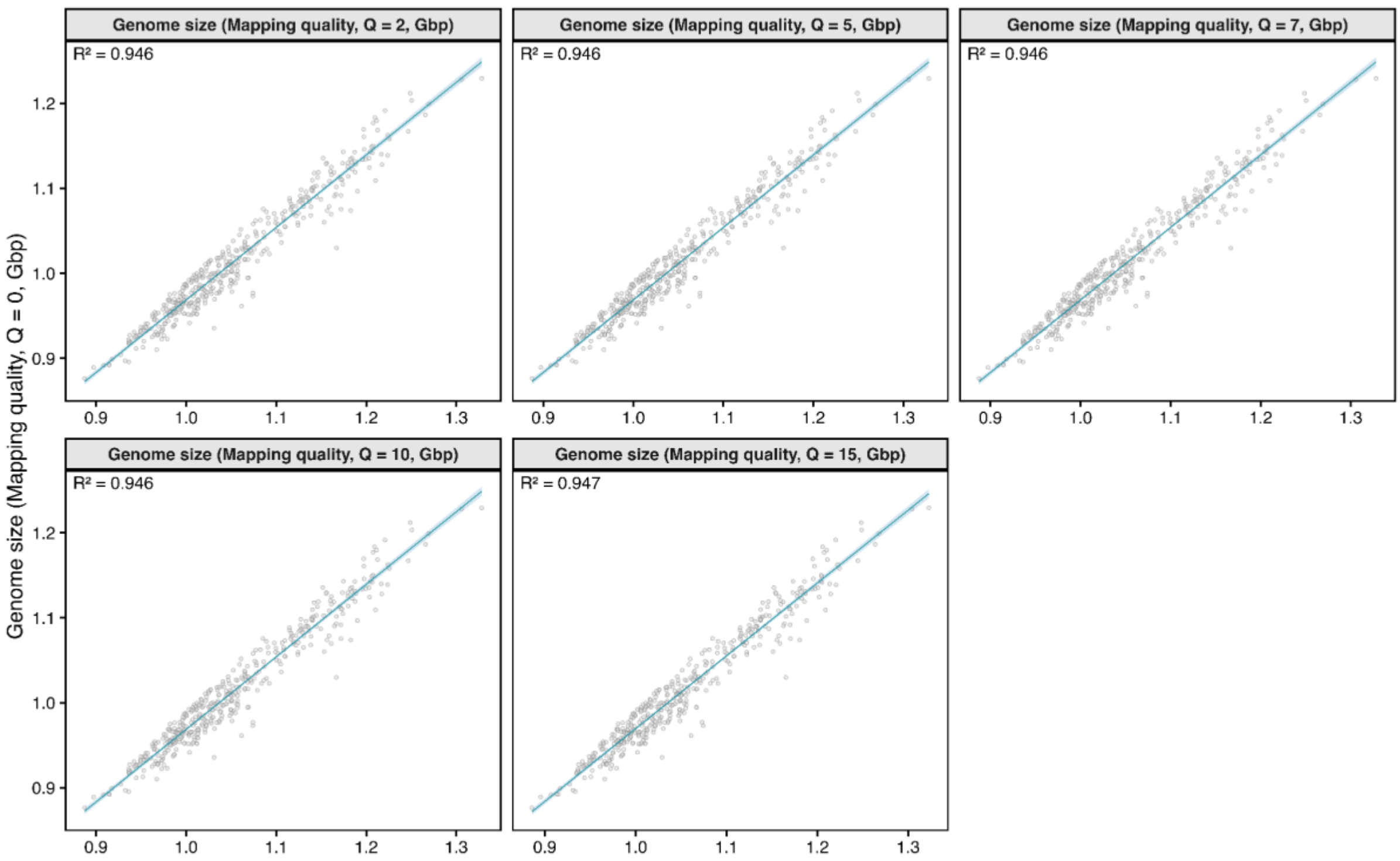

Figures S4: Histogram showing the frequency distribution of genome size in common ragweed (*Ambrosia artemisiifolia* L.), estimated by the sequence-based (genomic read-depth) method, across all sequenced individuals (n = 443). The mean and standard deviation (SD; the darker to light pink shade indicates from SD 1st to 3rd SD) of the genome size distribution (Gbp) are indicated on the plot.

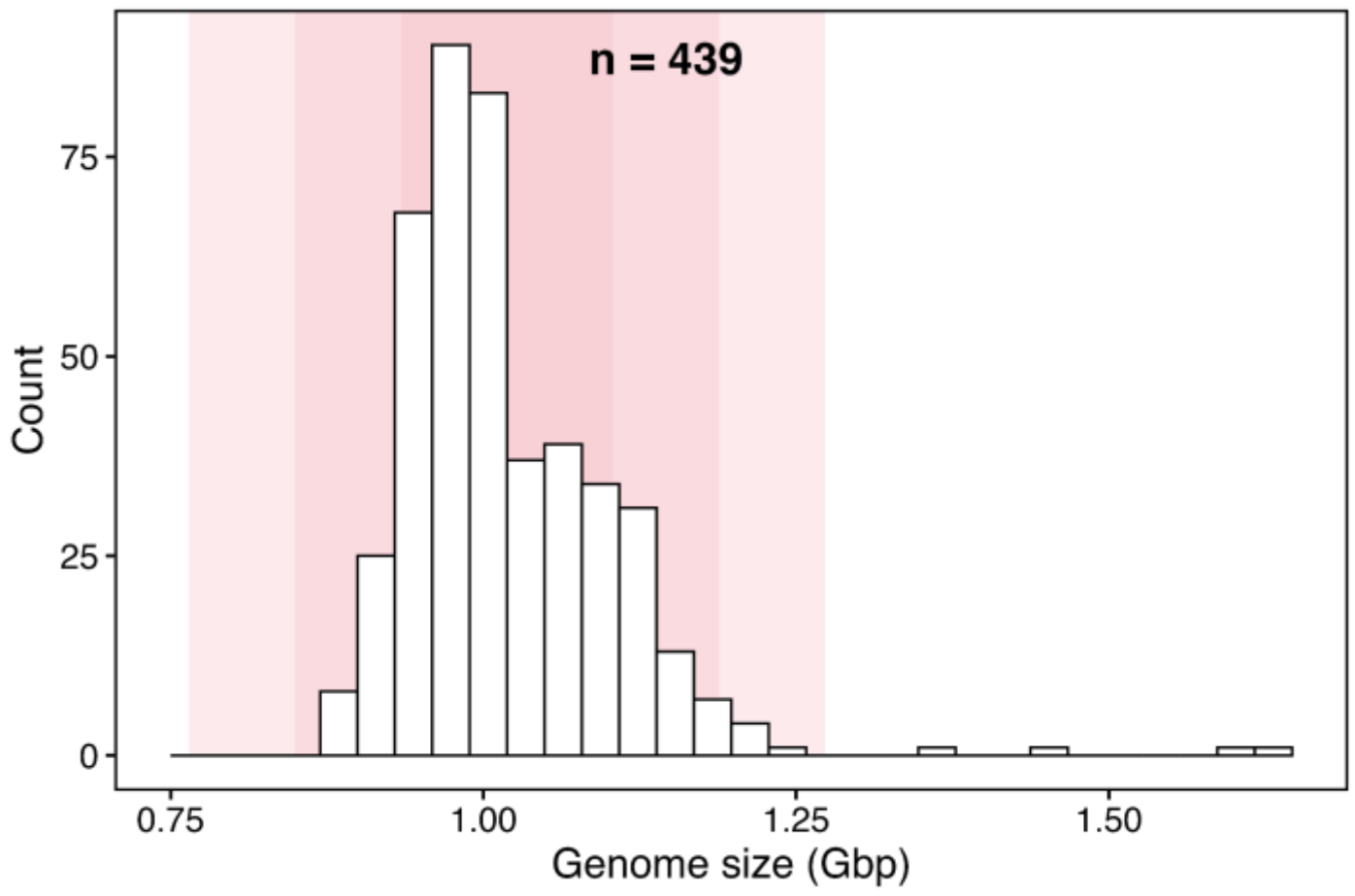

Figures S5: Assessment of sequencing coverage bias on TE abundance estimates in common ragweed (*Ambrosia artemisiifolia* L.). Scatter plots showing the relationships between sequencing coverage (mean depth over TE regions, ×) and abundance of different TE classes (Mbp) estimated from sequence data, across all sampled individuals.

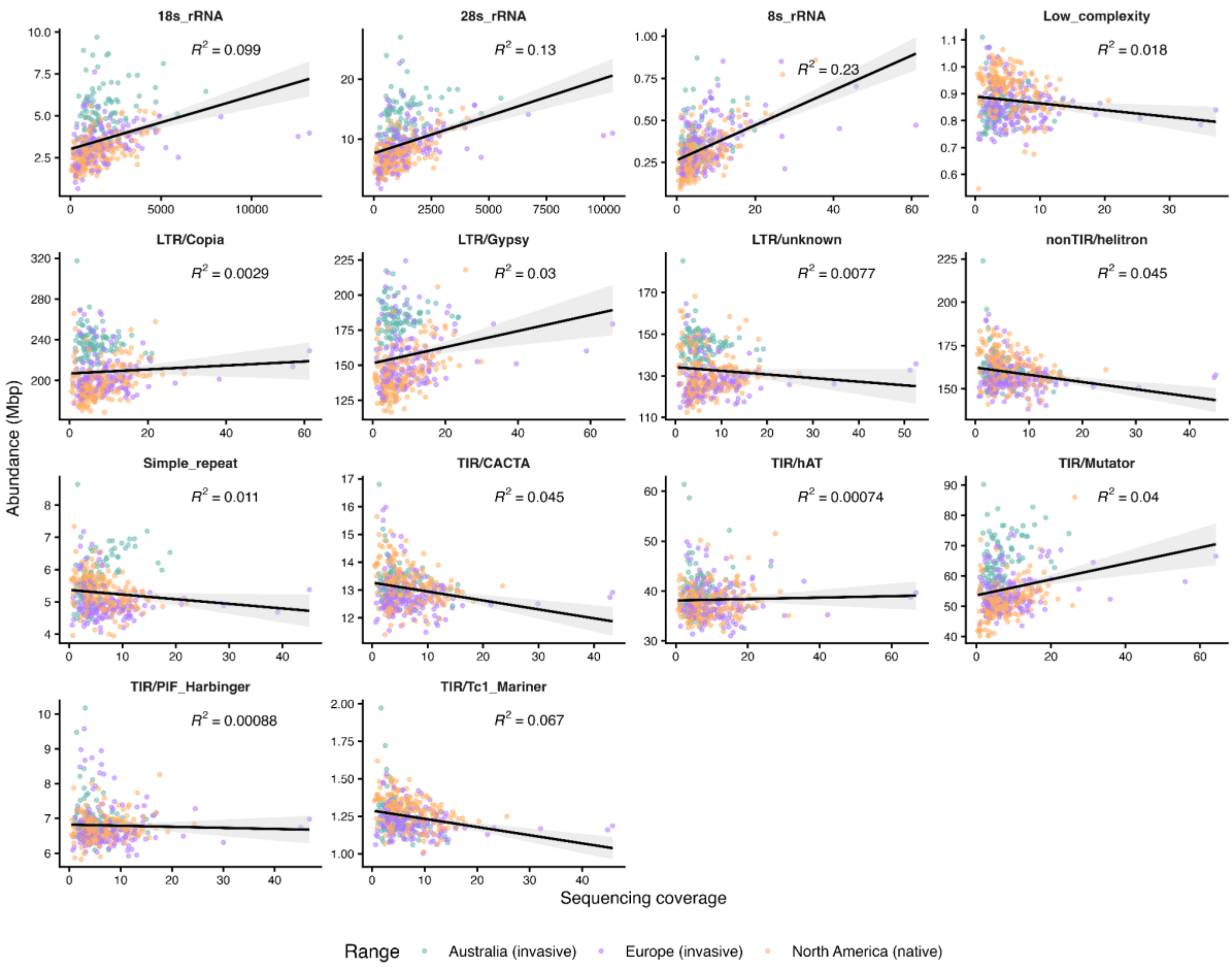

Figures S6: Identification of primary climatic predictors associated with common ragweed (*Ambrosia artemisiifolia* L.) populations. (a) Pearson correlation matrix among 19 bioclimatic variables (BIO1–BIO19; described in Tables S3) showing strong collinearity among temperature- and precipitation-related variables. (b) Principal component analysis (PCA) based on the subset of bioclimatic variables retained after collinearity filtering (Pearson's R < 0.70) together with mean annual temperature (MAT), illustrating the relative contribution of each variable to the first two principal components (PC1, PC2). (c) Pearson correlation between MAT and PC1, showing a strong association (r = 0.84) between MAT and the dominant climatic gradient across populations.

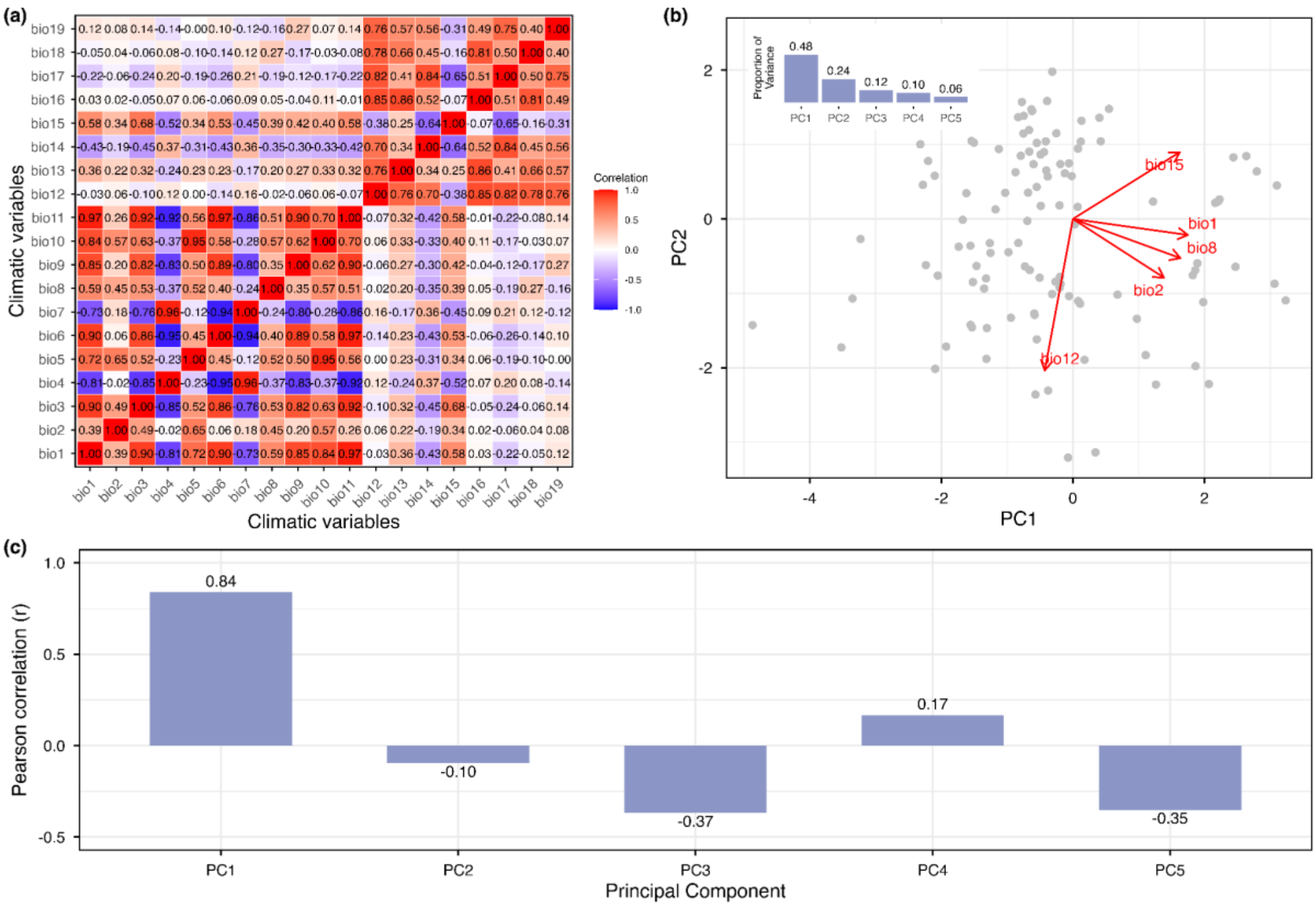

Figures S7: Estimation and comparison of genome size in cassava (*Manihot esculenta* Crantz) and common ragweed (*Ambrosia artemisiifolia* L.) by flow cytometry. Genome size was estimated for (a) a cassava sample, using tomato (*Solanum lycopersicum* var. *Stupické polní rané*) as the internal reference standard, and (b) a common ragweed sample, using cassava as the internal reference standard. Main plots show the flow cytometry fluorescence-intensity histograms used for genome size calculation; inset plots show the zoomed-out histograms of the corresponding gated nuclei populations used to identify the sample and standard peaks.

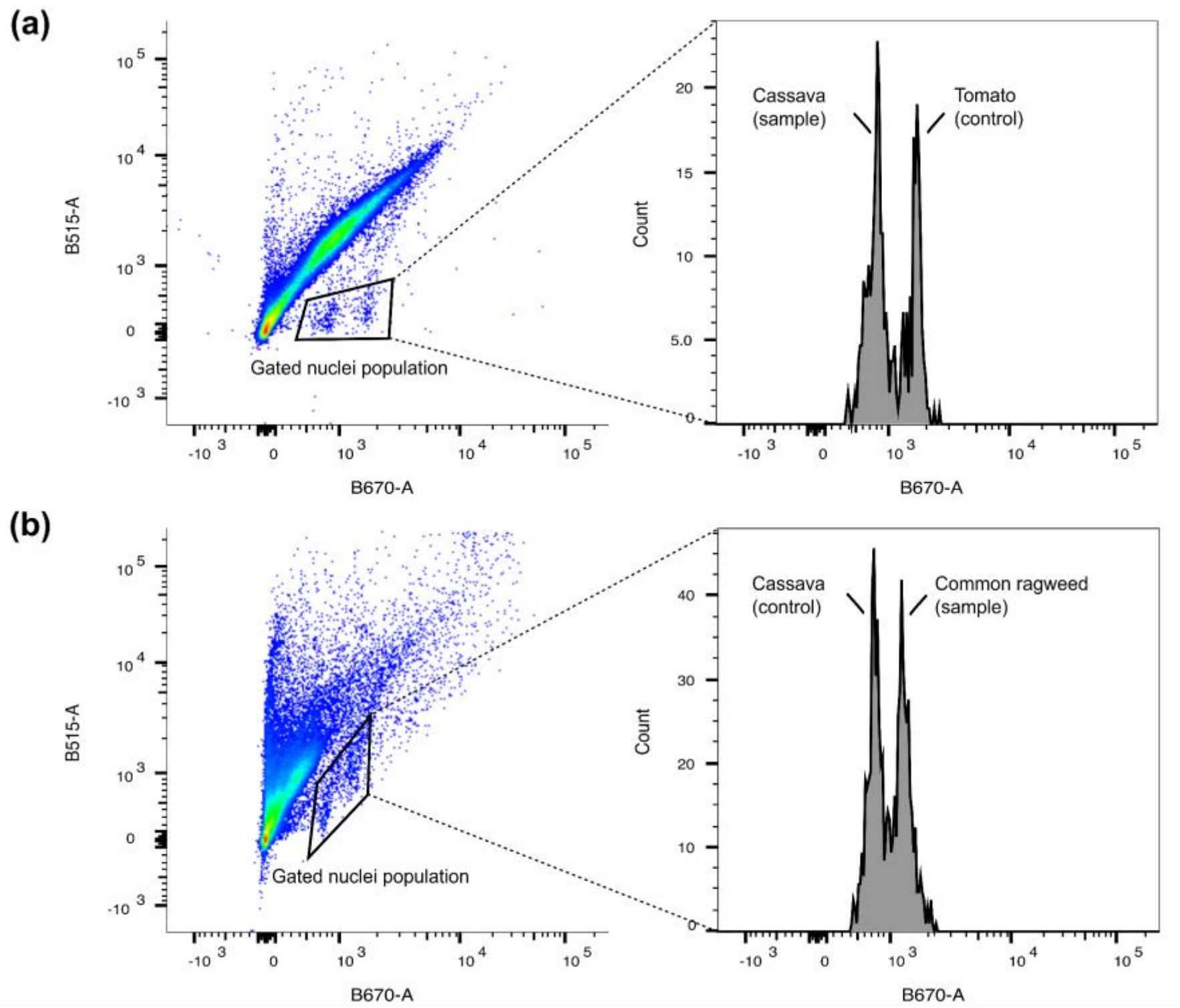

Figures S8: Distribution of genome size (Gbp) in common ragweed (*Ambrosia artemisiifolia* L.) estimated by flow-cytometry, across all measured individuals (n = 40).

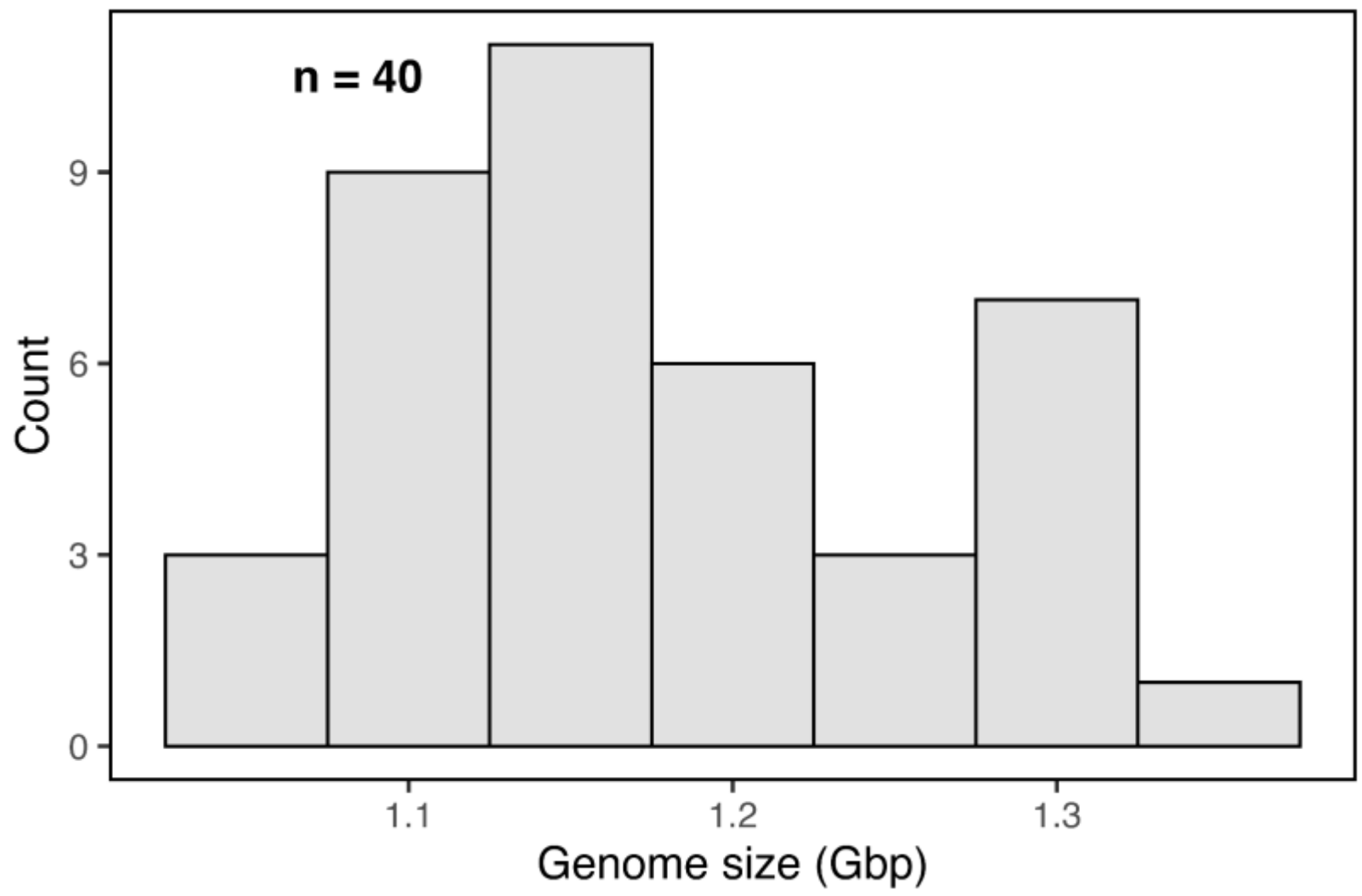

Figures S9: Comparison between the sequence-based and flow-cytometry-based genome size estimates (Gbp) in common ragweed (*Ambrosia artemisiifolia* L.). Scatter plot comparing genome size estimates obtained by the two methods for the same sampling locations, using individuals collected in different years and from different plants at each site (Tables S4). Absolute Pearson corelation analysis shows strong correlation (r = 0.94), showing the high consistency of estimation between the methods.

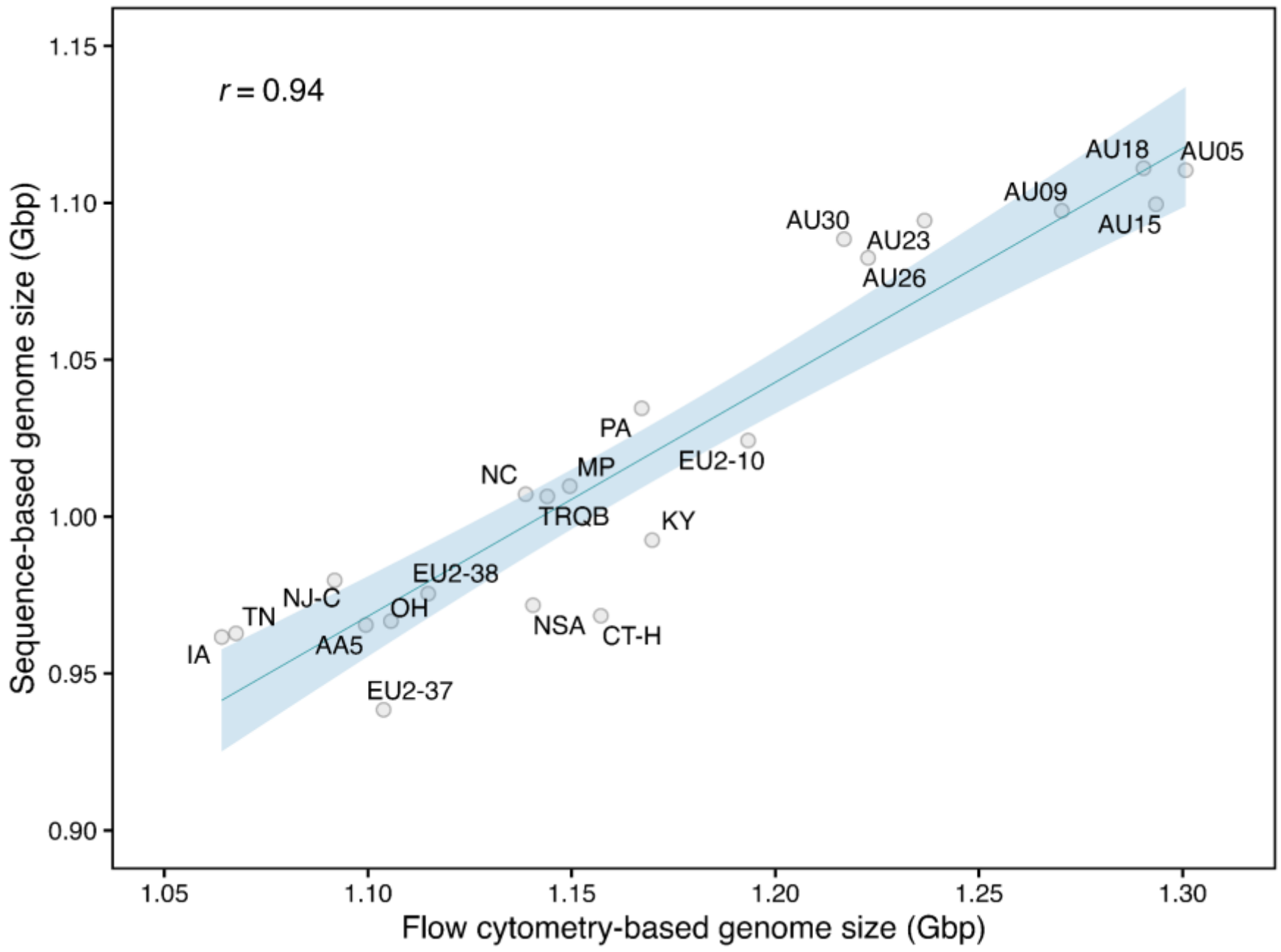

Figures S10: Comparison of TEs and rRNAs abundances between invasive ranges and their native populations of common ragweed (*Ambrosia artemisiifolia* L.). Box plots show TE and rRNAs abundance (Mbp) for Australia (AUS), Europe (EUR) and four spatio-genetic clusters within native North America: mid-east (NAM.ME), south (NAM.S), west (NAM.W) and east (NAM.E). Boxes show the interquartile range (IQR, 25th-75th percentile) of the raw data, with whiskers extending to 1.5 × IQR and individual values overlaid as points. Asterisks denote statistical significance from Tukey's honestly significant difference (HSD) post-hoc pairwise comparisons (***P < 0.001, **P < 0.01, *P < 0.05, ns = not significant).

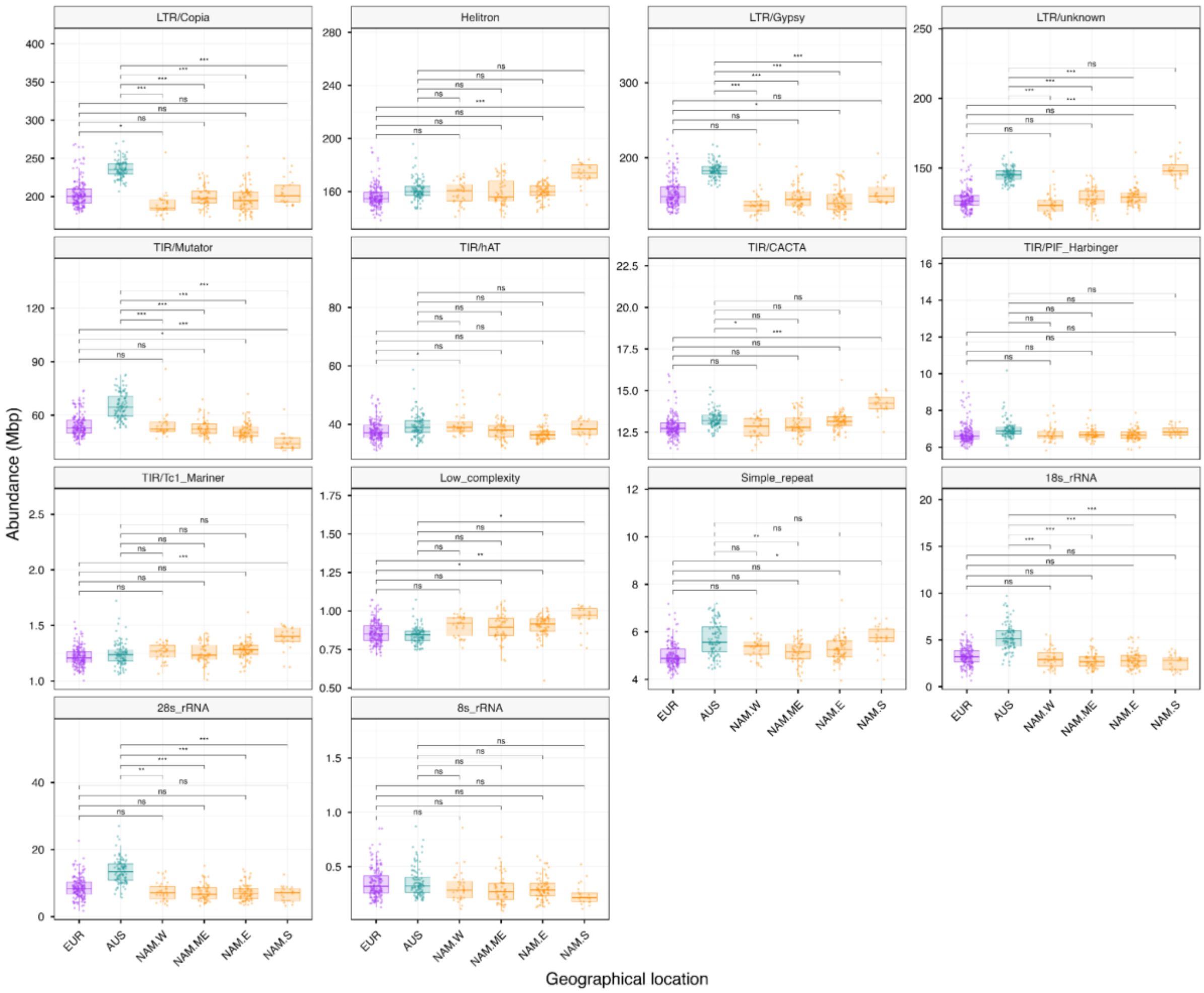

Figures S11: Proportion of TE and rRNA families in the genomes of individual common ragweed (*Ambrosia artemisiifolia* L.) samples from the native and invasive ranges. Stacked bar plot showing, for each individual, the relative proportion of each TE/rRNA family (family definitions in Tables S2) within its total annotated repeat content, grouped by range (native North America, NAM; invasive Europe, EUR; invasive Australia, AUS).

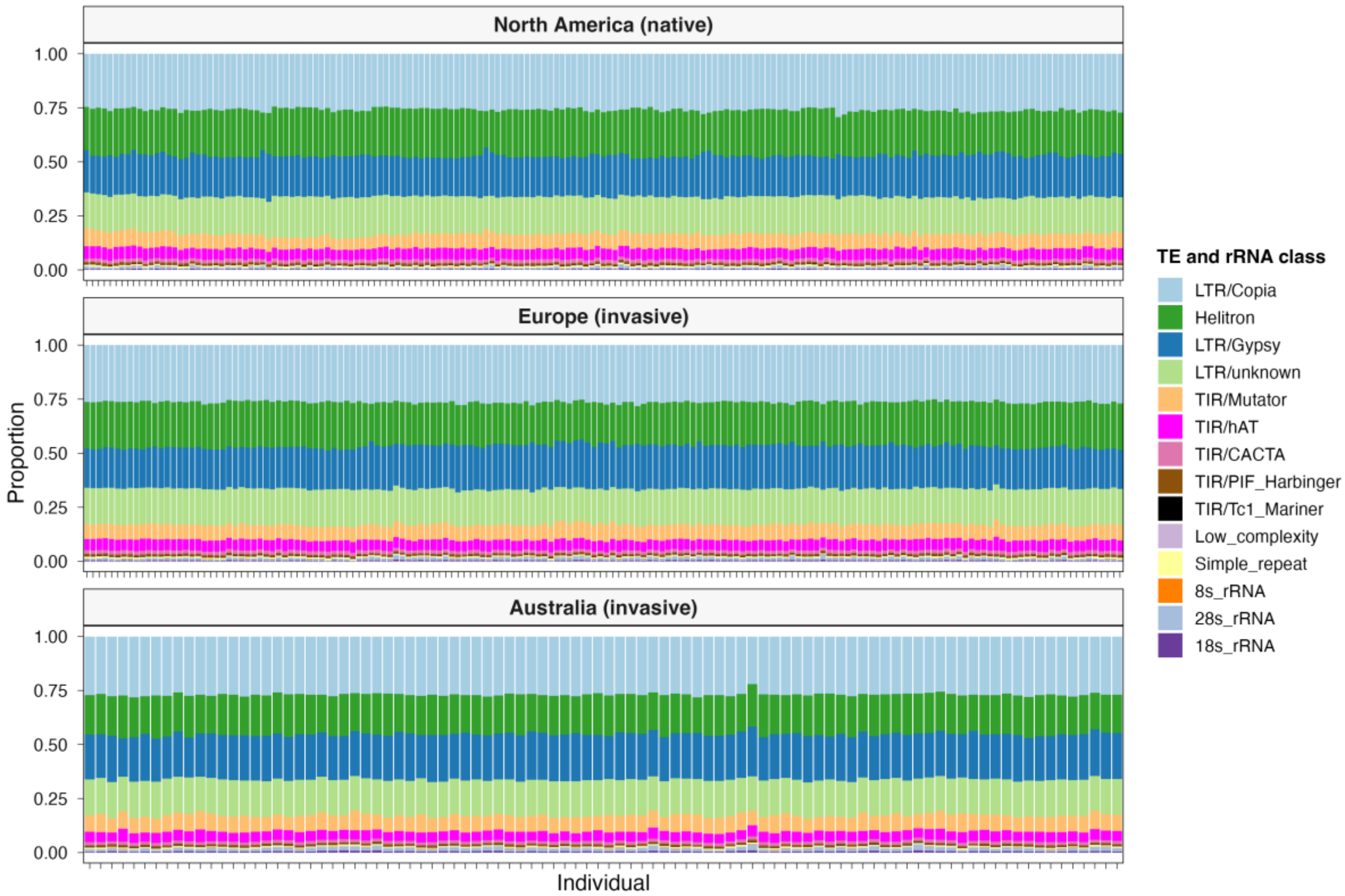

Figures S12: Abundance of different TE families across geographic ranges of common ragweed (*Ambrosia artemisiifolia* L.). Box plots show the abundance (Mbp) of each TE family for native North America (NAM) and the invasive ranges Europe (EUR) and Australia (AUS). Boxes show the interquartile range (IQR, 25th-75th percentile) of the raw data, with whiskers extending to 1.5 × IQR and individual values overlaid as points. Below each box, sample size (n) and the model-estimated mean ($\overline{X}$) ± standard error are reported. Asterisks denote statistical significance from Tukey's honestly significant difference (HSD) post-hoc pairwise comparisons (***P < 0.001, **P < 0.01, *P < 0.05, ns = not significant).

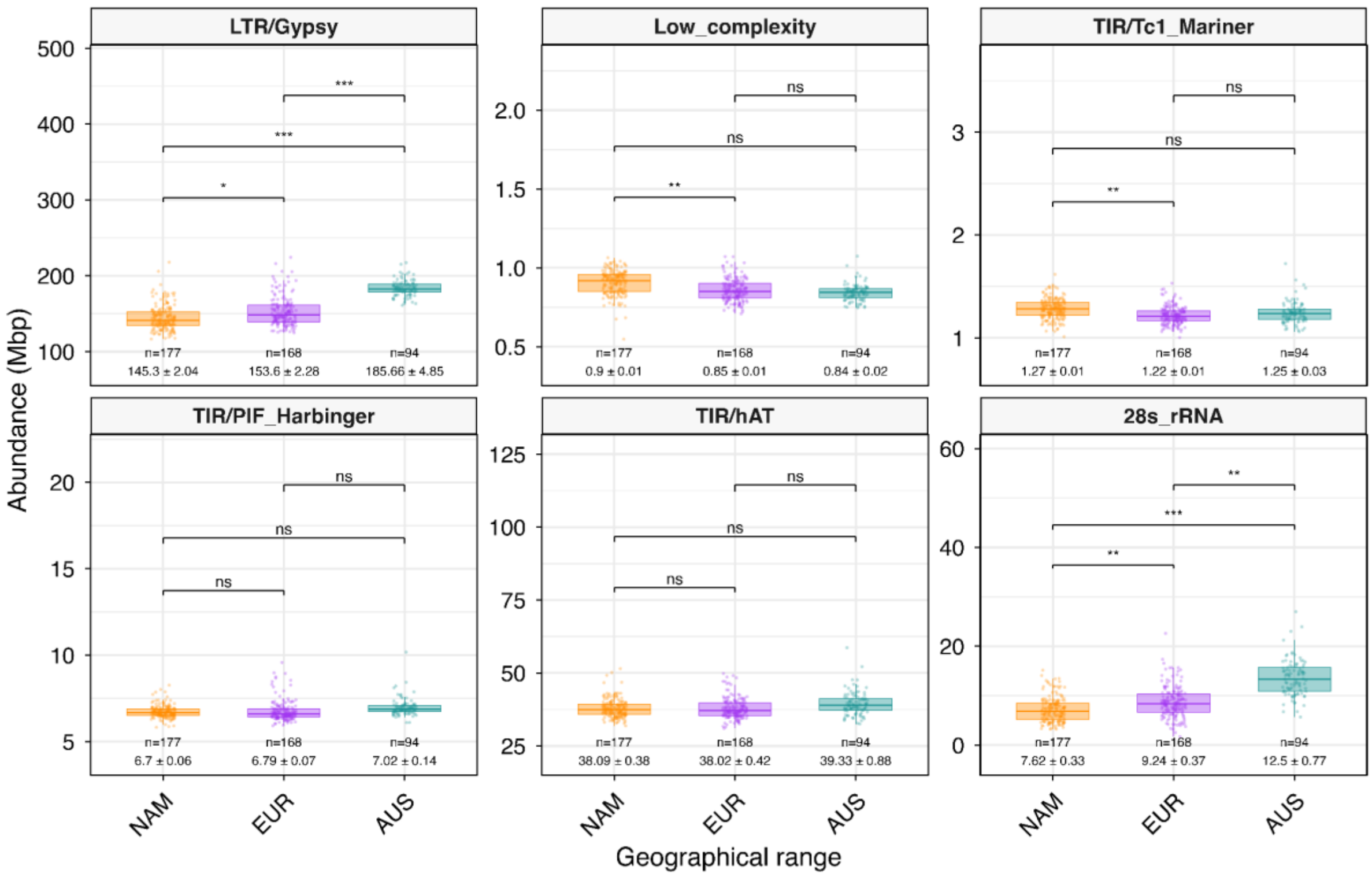

Figures S13: Comparison of genome size between two Australian clusters (AU01 and rest) and their native populations in North America of common ragweed (*Ambrosia artemisiifolia* L.). Box plots show genome size (Gbp) for two Australian clusters (AU01 and AUrest), and North America spatio-genetic clusters [mid-east (NAM.ME), south (NAM.S), west (NAM.W) and east (NAM.E)]. Boxes show the interquartile range (IQR, 25th-75th percentile) of the raw data, with whiskers extending to 1.5 × IQR and individual values overlaid as points. Sample size (n) is reported below each box. Asterisks denote statistical significance from Tukey's honestly significant difference (HSD) post-hoc pairwise comparisons (***P < 0.001, **P < 0.01, *P < 0.05, ns = not significant).

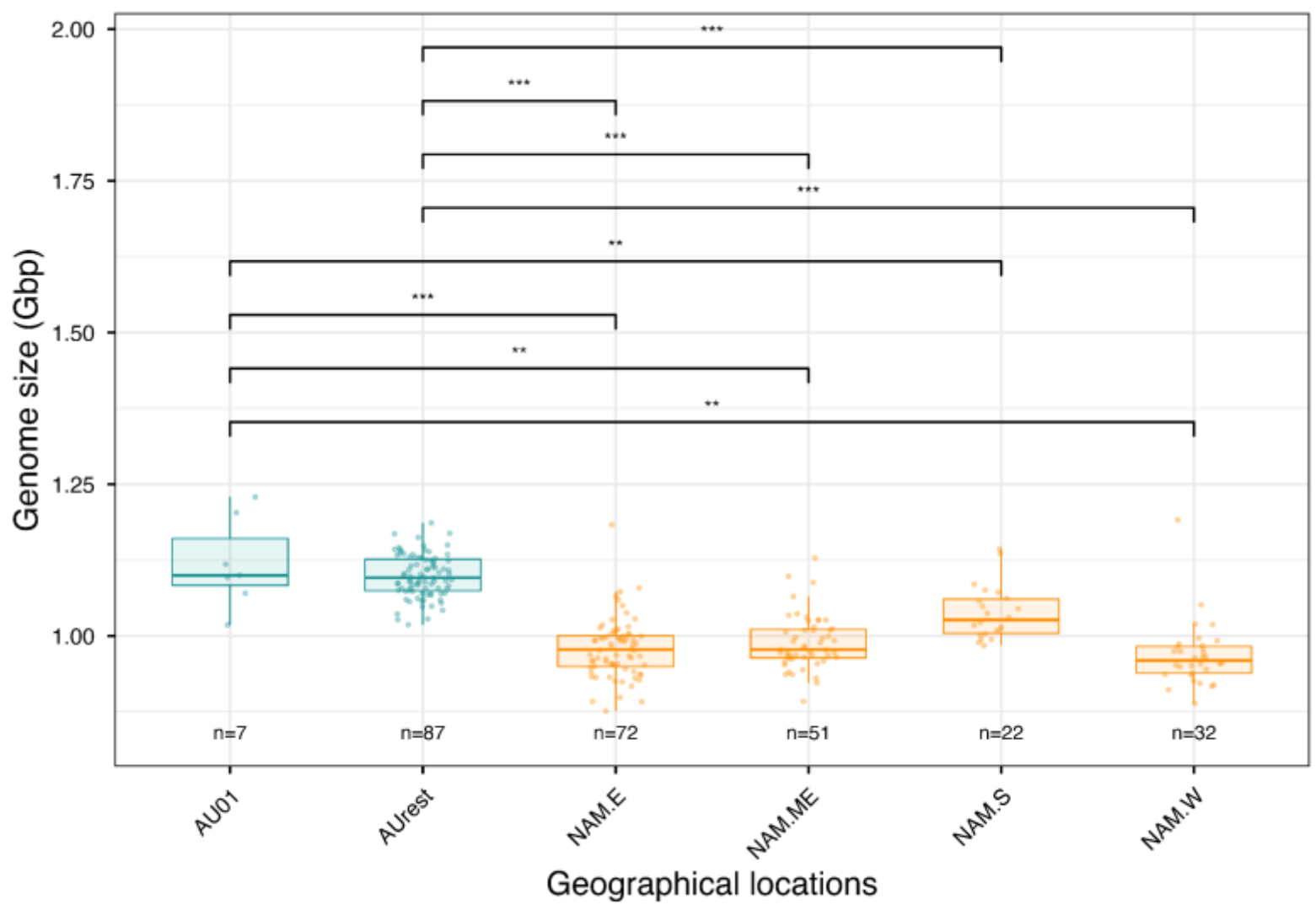

C) Supplementary Tables:

Tables S1. Sampling information for 443 individuals of common ragweed (*Ambrosia artemisiifolia* L.) analysed by sequence-based genome size estimation, spanning the native North American range (NAM) and the two invasive ranges of Europe (EUR) and Australia (AUS). For each individual, the table reports the sample ID, source population, spatio-genetic cluster assignment, geographic range, sampling latitude and longitude, sequencing coverage (whole genome mean depth, ×), sequence-based genome size estimate (Gbp), collection year, data source reference, and sequence read archive accession number(s). Spatio-genetic clusters are abbreviated as: North America (NAM.ME, mid-east; NAM.S, south; NAM.W, west; NAM.E, east) and Europe (EUR.ME, mid-east; EUR.W, west; EUR.E, east; EUR, other).

Sample ID	Population	Cluster	Range	Latitude	Longitude	Seq. coverage	Genome size (Gbp)	Year	Reference	Accession
280808-1-10	280808-1	EUR.ME	EUR	44.7249	22.4206	2.8000	0.9202	2013	Bieker et al. 2022	ERR7445133
280808-1-11	280808-1	EUR.ME	EUR	44.7249	22.4206	3.5736	0.9348	2013	Bieker et al. 2022	ERR7445144
280808-1-12	280808-1	EUR.ME	EUR	44.7249	22.4206	2.6493	0.9272	2013	Bieker et al. 2022	ERR7445115; ERR7445036
280808-1-16	280808-1	EUR.ME	EUR	44.7249	22.4206	11.4939	0.9678	2013	Bieker et al. 2022	ERR7445482
280808-1-17	280808-1	EUR.ME	EUR	44.7249	22.4206	10.5544	0.9912	2013	Bieker et al. 2022	ERR7445380
280808-1-24	280808-1	EUR.ME	EUR	44.7249	22.4206	2.5170	0.9041	2013	Bieker et al. 2022	ERR7445129
280808-1-3	280808-1	EUR.ME	EUR	44.7249	22.4206	3.2393	0.9360	2013	Bieker et al. 2022	ERR7445140
280808-1-5	280808-1	EUR.ME	EUR	44.7249	22.4206	2.5645	0.9646	2013	Bieker et al. 2022	ERR7445117
280808-1-8	280808-1	EUR.ME	EUR	44.7249	22.4206	2.8111	0.9324	2013	Bieker et al. 2022	ERR7445134
A-2010-T1-1	A-2010-T1	EUR	EUR	47.29309	11.0475	6.3297	0.9310	2010	Bieker et al. 2022	ERR7445199
A-2010-T1-10	A-2010-T1	EUR	EUR	47.29309	11.0475	6.7935	0.9815	2010	Bieker et al. 2022	ERR7445272
A-2010-T1-2	A-2010-T1	EUR	EUR	47.29309	11.0475	14.1224	1.0228	2010	Bieker et al. 2022	ERR7445574
A-2010-T1-3	A-2010-T1	EUR	EUR	47.29309	11.0475	8.6968	0.9512	2010	Bieker et al. 2022	ERR7445330

A-2010-T1-4	A-2010-T1	EUR	EUR	47.29309	11.0475	16.1132	0.9374	2010	Bieker et al. 2022	ERR7445580
A-2010-T1-5	A-2010-T1	EUR	EUR	47.29309	11.0475	18.3702	0.9532	2010	Bieker et al. 2022	ERR7445601
A-2010-T1-6	A-2010-T1	EUR	EUR	47.29309	11.0475	9.9380	1.0018	2010	Bieker et al. 2022	ERR7445349
A-2010-T1-7	A-2010-T1	EUR	EUR	47.29309	11.0475	3.5510	0.9912	2010	Bieker et al. 2022	ERR7445121
A-2010-T1-8	A-2010-T1	EUR	EUR	47.29309	11.0475	9.0957	0.9592	2010	Bieker et al. 2022	ERR7445311
A-2010-T1-9	A-2010-T1	EUR	EUR	47.29309	11.0475	7.7156	0.9203	2010	Bieker et al. 2022	ERR7445274
AA16-19	AA16	NAM.W	NAM	44.09456	-102.87	12.9417	1.0198	2013	Bieker et al. 2022	ERR7445552
AA16-2	AA16	NAM.W	NAM	44.09456	-102.87	5.0122	0.9357	2013	Bieker et al. 2022	ERR7445215
AA2-1	AA2	NAM.W	NAM	49.83778	-97.329	3.4365	0.9262	2013	Bieker et al. 2022	ERR7445189
AA2-4	AA2	NAM.W	NAM	49.83778	-97.329	2.4861	0.9173	2013	Bieker et al. 2022	ERR7445154
AA20-19	AA20	NAM.W	NAM	48.14689	-103.57	9.1155	1.4384	2013	Bieker et al. 2022	ERR7445475
AA20-26	AA20	NAM.W	NAM	48.14689	-103.57	9.0128	0.9372	2013	Bieker et al. 2022	ERR7445430
AA20-3	AA20	NAM.W	NAM	48.14689	-103.57	5.1965	0.8891	2013	Bieker et al. 2022	ERR7445214
AA3-15	AA3	NAM.W	NAM	48.19453	-97.329	4.6436	0.9493	2013	Bieker et al. 2022	ERR7445217
AA3-17	AA3	NAM.W	NAM	48.19453	-97.329	5.7912	0.9512	2013	Bieker et al. 2022	ERR7445249
AA3-23	AA3	NAM.W	NAM	48.19453	-97.329	9.5638	0.9869	2013	Bieker et al. 2022	ERR7445439
AA5-1	AA5	NAM.W	NAM	46.21708	-96.05	7.8922	0.9392	2013	Bieker et al. 2022	ERR7445491
AA5-16	AA5	NAM.W	NAM	46.21708	-96.05	5.1967	0.9747	2013	Bieker et al. 2022	ERR7445288
AA5-23	AA5	NAM.W	NAM	46.21708	-96.05	7.3603	0.9844	2013	Bieker et al. 2022	ERR7445467
AA5-9A	AA5	NAM.W	NAM	46.21708	-96.05	4.2347	0.9636	2013	Bieker et al. 2022	ERR7445306

AA7-12	AA7	NAM.W	NAM	44.738	-95.412	5.5950	1.0516	2013	Bieker et al. 2022	ERR7445368
AA7-24	AA7	NAM.W	NAM	44.738	-95.412	4.3831	0.9749	2013	Bieker et al. 2022	ERR7445353
AA7-27	AA7	NAM.W	NAM	44.738	-95.412	5.8029	0.9556	2013	Bieker et al. 2022	ERR7445443
AR-2019-1	AR-2019	NAM.ME	NAM	33.84188	-93.776	2.8629	0.9307	2009/ 2010	Bieker et al. 2022	ERR7445383
AR-2019-2	AR-2019	NAM.ME	NAM	33.84188	-93.776	2.5417	0.8923	2009/ 2010	Bieker et al. 2022	ERR7445379
AR-6	AR	NAM.ME	NAM	33.97552	-91.413	2.7851	0.9683	2013	Bieker et al. 2022	ERR7445351
AR-7	AR	NAM.ME	NAM	33.97552	-91.413	2.7351	0.9588	2013	Bieker et al. 2022	ERR7445361
AU01-1A	AU01	AUS	AUS	-35.6411	150.127	7.8092	1.1185	2014	Battlay et al. 2025	SRR30012807
AU01-2A	AU01	AUS	AUS	-35.6411	150.127	9.4214	1.1001	2014	Battlay et al. 2025	SRR30012806
AU01-3A	AU01	AUS	AUS	-35.6411	150.127	6.0965	1.0184	2014	Battlay et al. 2025	SRR30012795
AU01-6A	AU01	AUS	AUS	-35.6411	150.127	2.2997	1.2293	2014	Battlay et al. 2025	SRR30012760
AU01-6D	AU01	AUS	AUS	-35.6411	150.127	5.0966	1.0969	2014	Battlay et al. 2025	SRR30012749
AU01-8A	AU01	AUS	AUS	-35.6411	150.127	4.1771	1.2033	2014	Battlay et al. 2025	SRR30012786
AU01-8C	AU01	AUS	AUS	-35.6411	150.127	3.9732	1.0704	2014	Battlay et al. 2025	SRR30012735
AU05-11A	AU05	AUS	AUS	-28.7668	153.397	4.6142	1.1250	2014	Battlay et al. 2025	SRR30012780
AU05-14A	AU05	AUS	AUS	-28.7668	153.397	3.2988	1.1131	2014	Battlay et al. 2025	SRR30012769
AU05-16A	AU05	AUS	AUS	-28.7668	153.397	3.5193	1.1025	2014	Battlay et al. 2025	SRR30012726
AU05-19A	AU05	AUS	AUS	-28.7668	153.397	9.3811	1.1179	2014	Battlay et al. 2025	SRR30012805
AU05-19B	AU05	AUS	AUS	-28.7668	153.397	2.6870	1.1489	2014	Battlay et al. 2025	SRR30012804
AU05-1A	AU05	AUS	AUS	-28.7668	153.397	3.4504	1.0775	2014	Battlay et al. 2025	SRR30012803

AU05-20A	AU05	AUS	AUS	-28.7668	153.397	3.1082	1.0902	2014	Battlay et al. 2025	SRR30012802
AU05-3B	AU05	AUS	AUS	-28.7668	153.397	6.9851	1.1500	2014	Battlay et al. 2025	SRR30012801
AU05-4A	AU05	AUS	AUS	-28.7668	153.397	7.2379	1.1005	2014	Battlay et al. 2025	SRR30012800
AU05-5A	AU05	AUS	AUS	-28.7668	153.397	4.4765	1.0787	2014	Battlay et al. 2025	SRR30012799
AU09-1A	AU09	AUS	AUS	-28.8688	151.167	6.6782	1.0581	2014	Battlay et al. 2025	SRR30012798
AU09-21A	AU09	AUS	AUS	-28.8688	151.167	3.1120	1.1400	2014	Battlay et al. 2025	SRR30012797
AU09-22B	AU09	AUS	AUS	-28.8688	151.167	9.7181	1.0872	2014	Battlay et al. 2025	SRR30012796
AU09-29A	AU09	AUS	AUS	-28.8688	151.167	4.6370	1.1047	2014	Battlay et al. 2025	SRR30012794
AU11-11A	AU11	AUS	AUS	-25.3655	152.916	8.2515	1.1140	2014	Battlay et al. 2025	SRR30012793
AU11-15D	AU11	AUS	AUS	-25.3655	152.916	6.0123	1.0739	2014	Battlay et al. 2025	SRR30012792
AU11-1C	AU11	AUS	AUS	-25.3655	152.916	3.8444	1.1096	2014	Battlay et al. 2025	SRR30012767
AU11-24B	AU11	AUS	AUS	-25.3655	152.916	10.5748	1.1254	2014	Battlay et al. 2025	SRR30012766
AU11-5A	AU11	AUS	AUS	-25.3655	152.916	5.4231	1.1297	2014	Battlay et al. 2025	SRR30012765
AU11-5B	AU11	AUS	AUS	-25.3655	152.916	4.0555	1.1283	2014	Battlay et al. 2025	SRR30012764
AU15-10A	AU15	AUS	AUS	-27.3792	152.802	4.8975	1.0281	2014	Battlay et al. 2025	SRR30012763
AU15-14B	AU15	AUS	AUS	-27.3792	152.802	2.6132	1.0483	2014	Battlay et al. 2025	SRR30012762
AU15-16A	AU15	AUS	AUS	-27.3792	152.802	2.1405	1.0912	2014	Battlay et al. 2025	SRR30012761
AU15-17C	AU15	AUS	AUS	-27.3792	152.802	5.6532	1.1388	2014	Battlay et al. 2025	SRR30012759
AU15-21C	AU15	AUS	AUS	-27.3792	152.802	4.7134	1.1427	2014	Battlay et al. 2025	SRR30012758
AU15-23C	AU15	AUS	AUS	-27.3792	152.802	3.0344	1.1016	2014	Battlay et al. 2025	SRR30012757

AU15-26A	AU15	AUS	AUS	-27.3792	152.802	6.0016	1.1336	2014	Battlay et al. 2025	SRR30012756
AU15-2A	AU15	AUS	AUS	-27.3792	152.802	5.2124	1.1294	2014	Battlay et al. 2025	SRR30012755
AU15-9A	AU15	AUS	AUS	-27.3792	152.802	6.1289	1.0826	2014	Battlay et al. 2025	SRR30012754
AU18-13A	AU18	AUS	AUS	-27.785	153.275	1.8823	1.1417	2014	Battlay et al. 2025	SRR30012753
AU18-14B	AU18	AUS	AUS	-27.785	153.275	2.4700	1.1693	2014	Battlay et al. 2025	SRR30012752
AU18-19A	AU18	AUS	AUS	-27.785	153.275	17.0203	1.0703	2014	Battlay et al. 2025	SRR30012751
AU18-21A	AU18	AUS	AUS	-27.785	153.275	6.9691	1.0959	2014	Battlay et al. 2025	SRR30012750
AU18-25A	AU18	AUS	AUS	-27.785	153.275	4.1698	1.0681	2014	Battlay et al. 2025	SRR30012748
AU18-29A	AU18	AUS	AUS	-27.785	153.275	1.8191	1.1158	2014	Battlay et al. 2025	SRR30012747
AU18-3A	AU18	AUS	AUS	-27.785	153.275	3.9962	1.1191	2014	Battlay et al. 2025	SRR30012746
AU18-4A	AU18	AUS	AUS	-27.785	153.275	4.5392	1.0843	2014	Battlay et al. 2025	SRR30012745
AU18-5A	AU18	AUS	AUS	-27.785	153.275	2.8429	1.1358	2014	Battlay et al. 2025	SRR30012744
AU18-8B	AU18	AUS	AUS	-27.785	153.275	2.4166	1.1102	2014	Battlay et al. 2025	SRR30012791
AU19-10A	AU19	AUS	AUS	-28.0122	153.168	2.9225	1.1625	2014	Battlay et al. 2025	SRR30012790
AU19-11A	AU19	AUS	AUS	-28.0122	153.168	4.5244	1.0975	2014	Battlay et al. 2025	SRR30012789
AU19-14C	AU19	AUS	AUS	-28.0122	153.168	17.9820	1.0491	2014	Battlay et al. 2025	SRR30012788
AU19-21A	AU19	AUS	AUS	-28.0122	153.168	12.0686	1.1083	2014	Battlay et al. 2025	SRR30012787
AU19-22C	AU19	AUS	AUS	-28.0122	153.168	8.0477	1.1244	2014	Battlay et al. 2025	SRR30012785
AU19-23A	AU19	AUS	AUS	-28.0122	153.168	3.2424	1.1458	2014	Battlay et al. 2025	SRR30012784
AU19-25A	AU19	AUS	AUS	-28.0122	153.168	2.0696	1.0270	2014	Battlay et al. 2025	SRR30012743

AU19-29C	AU19	AUS	AUS	-28.0122	153.168	9.2488	1.0862	2014	Battlay et al. 2025	SRR30012742
AU19-30A	AU19	AUS	AUS	-28.0122	153.168	7.4467	1.0752	2014	Battlay et al. 2025	SRR30012741
AU19-3A	AU19	AUS	AUS	-28.0122	153.168	10.1767	1.0898	2014	Battlay et al. 2025	SRR30012740
AU23-10A	AU23	AUS	AUS	-28.9263	152.374	4.1243	1.0187	2014	Battlay et al. 2025	SRR30012739
AU23-14A	AU23	AUS	AUS	-28.9263	152.374	6.9166	1.0623	2014	Battlay et al. 2025	SRR30012738
AU23-21A	AU23	AUS	AUS	-28.9263	152.374	7.2025	1.0930	2014	Battlay et al. 2025	SRR30012737
AU23-24A	AU23	AUS	AUS	-28.9263	152.374	7.3190	1.1343	2014	Battlay et al. 2025	SRR30012736
AU23-28A	AU23	AUS	AUS	-28.9263	152.374	2.6303	1.1685	2014	Battlay et al. 2025	SRR30012734
AU23-30A	AU23	AUS	AUS	-28.9263	152.374	6.8927	1.0362	2014	Battlay et al. 2025	SRR30012733
AU23-5A	AU23	AUS	AUS	-28.9263	152.374	1.3554	1.1104	2014	Battlay et al. 2025	SRR30012732
AU23-6A	AU23	AUS	AUS	-28.9263	152.374	10.8769	1.1280	2014	Battlay et al. 2025	SRR30012731
AU23-8A	AU23	AUS	AUS	-28.9263	152.374	4.9003	1.0982	2014	Battlay et al. 2025	SRR30012730
AU26-11A	AU26	AUS	AUS	-30.0514	152.985	4.5953	1.0793	2014	Battlay et al. 2025	SRR30012729
AU26-13A	AU26	AUS	AUS	-30.0514	152.985	2.9149	1.0423	2014	Battlay et al. 2025	SRR30012728
AU26-16B	AU26	AUS	AUS	-30.0514	152.985	1.6488	1.0593	2014	Battlay et al. 2025	SRR30012783
AU26-1A	AU26	AUS	AUS	-30.0514	152.985	7.2188	1.0784	2014	Battlay et al. 2025	SRR30012782
AU26-20A	AU26	AUS	AUS	-30.0514	152.985	1.2543	1.0697	2014	Battlay et al. 2025	SRR30012781
AU26-22A	AU26	AUS	AUS	-30.0514	152.985	1.5148	1.3631	2014	Battlay et al. 2025	SRR30012779
AU26-28A	AU26	AUS	AUS	-30.0514	152.985	13.6542	1.1343	2014	Battlay et al. 2025	SRR30012778
AU26-29A	AU26	AUS	AUS	-30.0514	152.985	3.7611	1.0545	2014	Battlay et al. 2025	SRR30012777

AU26-30B	AU26	AUS	AUS	-30.0514	152.985	5.2885	1.0889	2014	Battlay et al. 2025	SRR30012776
AU26-8A	AU26	AUS	AUS	-30.0514	152.985	5.2690	1.1354	2014	Battlay et al. 2025	SRR30012775
AU27-10A	AU27	AUS	AUS	-30.2413	152.585	5.7891	1.1092	2014	Battlay et al. 2025	SRR30012774
AU27-16D	AU27	AUS	AUS	-30.2413	152.585	1.3188	1.0917	2014	Battlay et al. 2025	SRR30012773
AU27-21A	AU27	AUS	AUS	-30.2413	152.585	4.1800	1.0696	2014	Battlay et al. 2025	SRR30012772
AU27-22C	AU27	AUS	AUS	-30.2413	152.585	18.9797	1.1028	2014	Battlay et al. 2025	SRR30012771
AU27-27A	AU27	AUS	AUS	-30.2413	152.585	10.4961	1.0843	2014	Battlay et al. 2025	SRR30012770
AU27-2A	AU27	AUS	AUS	-30.2413	152.585	4.0966	1.1429	2014	Battlay et al. 2025	SRR30012768
AU27-2B	AU27	AUS	AUS	-30.2413	152.585	2.4224	1.1304	2014	Battlay et al. 2025	SRR30012815
AU27-30A	AU27	AUS	AUS	-30.2413	152.585	5.4431	1.0754	2014	Battlay et al. 2025	SRR30012814
AU27-4A	AU27	AUS	AUS	-30.2413	152.585	5.6376	1.0856	2014	Battlay et al. 2025	SRR30012813
AU27-9A	AU27	AUS	AUS	-30.2413	152.585	6.2148	1.0675	2014	Battlay et al. 2025	SRR30012812
AU30-12A	AU30	AUS	AUS	-30.7406	152.915	3.8024	1.1864	2014	Battlay et al. 2025	SRR30012811
AU30-13A	AU30	AUS	AUS	-30.7406	152.915	5.0562	1.0601	2014	Battlay et al. 2025	SRR30012810
AU30-16A	AU30	AUS	AUS	-30.7406	152.915	4.7620	1.0738	2014	Battlay et al. 2025	SRR30012809
AU30-17B	AU30	AUS	AUS	-30.7406	152.915	3.1219	1.0566	2014	Battlay et al. 2025	SRR30012808
AU30-18A	AU30	AUS	AUS	-30.7406	152.915	2.9875	1.0782	2014	Battlay et al. 2025	SRR30012727
AU30-1A	AU30	AUS	AUS	-30.7406	152.915	2.4033	1.0871	2014	Battlay et al. 2025	SRR30012725
AU30-21B	AU30	AUS	AUS	-30.7406	152.915	5.2359	1.0708	2014	Battlay et al. 2025	SRR30012724
AU30-24A	AU30	AUS	AUS	-30.7406	152.915	11.7052	1.1226	2014	Battlay et al. 2025	SRR30012723

AU30-28A	AU30	AUS	AUS	-30.7406	152.915	9.9673	1.0905	2014	Battlay et al. 2025	SRR30012722
AU30-29A	AU30	AUS	AUS	-30.7406	152.915	8.0096	1.0586	2014	Battlay et al. 2025	SRR30012721
B-1	B	EUR.ME	EUR	44.90308	-0.5352	3.5525	0.9731	2019	Bieker et al. 2022	ERR7445587
B-2	B	EUR.W	EUR	44.90308	-0.5352	2.6415	0.9516	2019	Bieker et al. 2022	ERR7445449
B-3	B	EUR.ME	EUR	44.90308	-0.5352	3.2873	0.9770	2019	Bieker et al. 2022	ERR7445568
B-4	B	EUR.W	EUR	44.90308	-0.5352	4.8650	0.9614	2019	Bieker et al. 2022	ERR7445618
B1-1	B1	EUR	EUR	47.87951	18.1561	2.5077	1.0298	2014	Bieker et al. 2022	ERR7445405
B2-1	B1	EUR	EUR	47.87951	18.1561	5.7722	0.9184	2014	Bieker et al. 2022	ERR7445510
B3-1	B1	EUR	EUR	47.87951	18.1561	3.8043	1.0001	2014	Bieker et al. 2022	ERR7445424
BN-ON-6	BN-ON	NAM.ME	NAM	45	-77.7	15.5509	0.9773	2013	Bieker et al. 2022	ERR7445918
Caluire-26	Caluire	EUR.ME	EUR	45.99	4.84	3.3096	1.0024	2017	Bieker et al. 2022	ERR7445586
Caluire-27	Caluire	EUR.ME	EUR	45.99	4.84	3.7465	0.9695	2017	Bieker et al. 2022	ERR7445595
Caluire-31	Caluire	EUR.ME	EUR	45.99	4.84	4.7851	1.0189	2017	Bieker et al. 2022	ERR7445609
Caluire-32	Caluire	EUR.ME	EUR	45.99	4.84	4.5922	0.9500	2017	Bieker et al. 2022	ERR7445641
Caluire-36	Caluire	EUR.ME	EUR	45.99	4.84	5.2947	1.0228	2017	Bieker et al. 2022	ERR7445702
Caluire-37	Caluire	EUR.ME	EUR	45.99	4.84	2.2943	0.9269	2017	Bieker et al. 2022	ERR7445612
Caluire-41	Caluire	EUR.ME	EUR	45.99	4.84	4.3034	1.0238	2017	Bieker et al. 2022	ERR7445690
Caluire-42	Caluire	EUR.ME	EUR	45.99	4.84	3.3282	0.9984	2017	Bieker et al. 2022	ERR7445643
Caluire-46	Caluire	EUR.ME	EUR	45.99	4.84	4.8987	1.0116	2017	Bieker et al. 2022	ERR7445685
Caluire-47	Caluire	EUR.ME	EUR	45.99	4.84	3.1805	1.0626	2017	Bieker et al. 2022	ERR7445649

CT-H-1	CT-H	NAM.E	NAM	41.2877	-73.766	12.4788	0.9406	2009	Bieker et al. 2022	ERR7445851
CT-H-10	CT-H	NAM.E	NAM	41.2877	-73.766	3.9864	0.9875	2009	Bieker et al. 2022	ERR7445507
CT-H-2	CT-H	NAM.E	NAM	41.2877	-73.766	4.8670	1.0041	2009	Bieker et al. 2022	ERR7445527
CT-H-3	CT-H	NAM.E	NAM	41.2877	-73.766	6.0880	0.9690	2009	Bieker et al. 2022	ERR7445571
CT-H-4	CT-H	NAM.E	NAM	41.2877	-73.766	8.5146	0.9764	2009	Bieker et al. 2022	ERR7445599
CT-H-6	CT-H	NAM.E	NAM	41.2877	-73.766	4.4905	0.9173	2009	Bieker et al. 2022	ERR7445588
CT-H-7	CT-H	NAM.E	NAM	41.2877	-73.766	2.7977	0.9687	2009	Bieker et al. 2022	ERR7445539
CT-H-8	CT-H	NAM.E	NAM	41.2877	-73.766	3.9002	0.9979	2009	Bieker et al. 2022	ERR7445563
CT-H-9	CT-H	NAM.E	NAM	41.2877	-73.766	5.3828	0.9542	2009	Bieker et al. 2022	ERR7445598
D-2017-4-10	D-2017-4	EUR.E	EUR	52.8243	13.7691	12.1407	0.9759	2017	Bieker et al. 2022	ERR7446018
D-2017-4-2	D-2017-4	EUR.E	EUR	52.8243	13.7691	12.3416	0.9948	2017	Bieker et al. 2022	ERR7446048
D-2017-4-3	D-2017-4	EUR.E	EUR	52.8243	13.7691	12.5795	0.9564	2017	Bieker et al. 2022	ERR7446052
D-2017-4-4	D-2017-4	EUR.E	EUR	52.8243	13.7691	3.7315	0.9415	2017	Bieker et al. 2022	ERR7445696
D-2017-4-5	D-2017-4	EUR.E	EUR	52.8243	13.7691	7.5742	0.9965	2017	Bieker et al. 2022	ERR7445883
D-2017-4-6	D-2017-4	EUR.E	EUR	52.8243	13.7691	6.7693	0.9563	2017	Bieker et al. 2022	ERR7445759
D-2017-4-7	D-2017-4	EUR.E	EUR	52.8243	13.7691	8.3414	0.9770	2017	Bieker et al. 2022	ERR7445900
D-2017-4-9	D-2017-4	EUR.E	EUR	52.8243	13.7691	9.3081	0.9946	2017	Bieker et al. 2022	ERR7445944
EU2-01-12	EU2-01	EUR.ME	EUR	46.03608	15.2961	1.0341	0.8956	2014	Bieker et al. 2022	ERR7445630
EU2-02-1	EU2-02	EUR	EUR	47.13087	16.9031	4.8009	1.0026	2014	Bieker et al. 2022	ERR7445722
EU2-02-10	EU2-02	EUR	EUR	47.13087	16.9031	9.7312	1.1189	2014	Bieker et al. 2022	ERR7451270; ERR7445700

EU2-03-9C	EU2-03	EUR.ME	EUR	47.3278	19.7307	12.3793	0.9902	2014	Bieker et al. 2022	ERR7446032
EU2-04-11	EU2-04	EUR	EUR	47.87992	18.1545	3.1462	0.9829	2014	Bieker et al. 2022	ERR7445676
EU2-04-12	EU2-04	EUR	EUR	47.87992	18.1545	5.2942	0.9978	2014	Bieker et al. 2022	ERR7445760
EU2-05-18	EU2-05	EUR.ME	EUR	45.46996	12.1092	2.3676	0.9650	2014	Bieker et al. 2022	ERR7445672
EU2-05-23	EU2-05	EUR.ME	EUR	45.46996	12.1092	6.6578	0.9990	2014	Bieker et al. 2022	ERR7445823
EU2-05-25	EU2-05	EUR.ME	EUR	45.46996	12.1092	3.9722	1.5958	2014	Bieker et al. 2022	ERR7448827; ERR7445768
EU2-06-15D	EU2-06	EUR.ME	EUR	45.57073	8.78546	14.8513	0.9102	2014	Bieker et al. 2022	ERR7446171
EU2-06-18	EU2-06	EUR.ME	EUR	45.57073	8.78546	11.8556	0.9900	2014	Bieker et al. 2022	ERR7445725
EU2-08-13	EU2-08	EUR.ME	EUR	45.93087	8.98377	13.3247	0.9725	2014	Bieker et al. 2022	ERR7446122
EU2-08-21	EU2-08	EUR.ME	EUR	45.93087	8.98377	8.6689	0.9977	2014	Bieker et al. 2022	ERR7445996
EU2-08-4	EU2-08	EUR.ME	EUR	45.93087	8.98377	50.8313	1.0283	2014	Bieker et al. 2022	ERR7446626
Eu2-09-1	EU2-09	EUR.W	EUR	45.06541	7.59229	8.9294	0.9787	2014	Bieker et al. 2022	ERR7446514
EU2-09-10	EU2-09	EUR.W	EUR	45.06541	7.59229	5.4996	1.0298	2014	Bieker et al. 2022	ERR7445820
EU2-09-13	EU2-09	EUR.W	EUR	45.06541	7.59229	0.8566	0.9847	2014	Bieker et al. 2022	ERR7445744
EU2-10-10	EU2-10	EUR.ME	EUR	43.93235	4.32049	8.9118	1.0286	2014	Bieker et al. 2022	ERR7451305
EU2-10-11	EU2-10	EUR.ME	EUR	43.93235	4.32049	8.9988	1.0819	2014	Bieker et al. 2022	ERR7451254; ERR7445819
EU2-10-12	EU2-10	EUR.ME	EUR	43.93235	4.32049	13.7131	0.9278	2014	Bieker et al. 2022	ERR7451241; ERR7445891
EU2-10-18	EU2-10	EUR.ME	EUR	43.93235	4.32049	8.2960	1.0439	2014	Bieker et al. 2022	ERR7448828; ERR7445989
EU2-10-2	EU2-10	EUR.ME	EUR	43.93235	4.32049	10.7402	1.0359	2014	Bieker et al. 2022	ERR7448846; ERR7446012
EU2-10-3	EU2-10	EUR.ME	EUR	43.93235	4.32049	4.8717	1.0325	2014	Bieker et al. 2022	ERR7448849

EU2-10-4	EU2-10	EUR.ME	EUR	43.93235	4.32049	15.2390	1.0144	2014	Bieker et al. 2022	ERR7451306; ERR7459478
EU2-10-5	EU2-10	EUR.ME	EUR	43.93235	4.32049	18.4520	1.0550	2014	Bieker et al. 2022	ERR7451323; ERR7446128
EU2-10-7	EU2-10	EUR.ME	EUR	43.93235	4.32049	8.3755	1.0092	2014	Bieker et al. 2022	ERR7451219; ERR7445878
EU2-10-9	EU2-10	EUR.ME	EUR	43.93235	4.32049	15.5886	1.0134	2014	Bieker et al. 2022	ERR7451257; ERR7446103
EU2-12-13	EU2-12	EUR.ME	EUR	46.66434	4.3278	6.8227	0.9650	2014	Bieker et al. 2022	ERR7445959
EU2-14-12	EU2-14	EUR.ME	EUR	47.4556	5.21213	11.1351	0.9982	2014	Bieker et al. 2022	ERR7446267
EU2-14-13	EU2-14	EUR.ME	EUR	47.4556	5.21213	6.3861	1.1796	2014	Bieker et al. 2022	ERR7448877; ERR7445939
EU2-16-1	EU2-16	EUR.ME	EUR	46.1622	6.0094	8.4323	1.0306	2014	Bieker et al. 2022	ERR7446143
EU2-16-12	EU2-16	EUR.ME	EUR	46.1622	6.0094	11.4039	1.0138	2014	Bieker et al. 2022	ERR7446240
EU2-16-13	EU2-16	EUR.ME	EUR	46.1622	6.0094	7.9713	0.9523	2014	Bieker et al. 2022	ERR7446153
EU2-17-1	EU2-17	EUR	EUR	51.12003	5.8403	13.8939	1.0430	2014	Bieker et al. 2022	ERR7448842; ERR7446254
EU2-17-10	EU2-17	EUR	EUR	51.12003	5.8403	18.0630	1.0650	2014	Bieker et al. 2022	ERR7448838; ERR7446424
EU2-17-11	EU2-17	EUR	EUR	51.12003	5.8403	52.7390	1.0643	2014	Bieker et al. 2022	ERR7448883; ERR7448735
EU2-17-2	EU2-17	EUR	EUR	51.12003	5.8403	26.0648	1.0454	2014	Bieker et al. 2022	ERR7448845; ERR7446484
EU2-17-3	EU2-17	EUR	EUR	51.12003	5.8403	15.7182	1.0717	2014	Bieker et al. 2022	ERR7448841; ERR7446403
EU2-17-5	EU2-17	EUR	EUR	51.12003	5.8403	11.8416	0.9560	2014	Bieker et al. 2022	ERR7448851; ERR7446234
EU2-17-6	EU2-17	EUR	EUR	51.12003	5.8403	13.6380	0.9268	2014	Bieker et al. 2022	ERR7446431
EU2-17-7	EU2-17	EUR	EUR	51.12003	5.8403	13.6515	1.0263	2014	Bieker et al. 2022	ERR7448850; ERR7446300
EU2-17-8	EU2-17	EUR	EUR	51.12003	5.8403	7.3054	1.1315	2014	Bieker et al. 2022	ERR7448858; ERR7446151
EU2-17-9	EU2-17	EUR	EUR	51.12003	5.8403	7.6998	1.0897	2014	Bieker et al. 2022	ERR7448859; ERR7446146

EU2-19-1	EU2-19	EUR	EUR	56.16811	15.8904	2.1869	1.2119	2014	Bieker et al. 2022	ERR7446125
EU2-19-2	EU2-19	EUR.ME	EUR	56.16811	15.8904	1.6688	1.0159	2014	Bieker et al. 2022	ERR7446159
EU2-20-1	EU2-20	EUR.E	EUR	51.63281	14.1844	4.4941	1.0168	2014	Bieker et al. 2022	ERR7446204
EU2-20-10	EU2-20	EUR.E	EUR	51.63281	14.1844	6.5836	1.1608	2014	Bieker et al. 2022	ERR7448869; ERR7446195
EU2-20-11	EU2-20	EUR.E	EUR	51.63281	14.1844	7.8623	1.0114	2014	Bieker et al. 2022	ERR7446281
EU2-21-13	EU2-21	EUR	EUR	50.19005	15.0604	10.6246	0.9953	2014	Bieker et al. 2022	ERR7446433
EU2-21-14	EU2-21	EUR	EUR	50.19005	15.0604	10.5727	0.9792	2014	Bieker et al. 2022	ERR7446435
EU2-21-16	EU2-21	EUR	EUR	50.19005	15.0604	34.4461	0.9786	2014	Bieker et al. 2022	ERR7446589
EU2-21-18	EU2-21	EUR	EUR	50.19005	15.0604	10.0232	0.9698	2014	Bieker et al. 2022	ERR7446430
EU2-21-20	EU2-21	EUR	EUR	50.19005	15.0604	11.1300	0.9915	2014	Bieker et al. 2022	ERR7446439
EU2-21-22	EU2-21	EUR	EUR	50.19005	15.0604	4.6874	0.9640	2014	Bieker et al. 2022	ERR7446288
EU2-21-24	EU2-21	EUR	EUR	50.19005	15.0604	9.9167	1.0089	2014	Bieker et al. 2022	ERR7446438
EU2-21-27	EU2-21	EUR	EUR	50.19005	15.0604	5.8702	0.9853	2014	Bieker et al. 2022	ERR7446349
EU2-21-28	EU2-21	EUR	EUR	50.19005	15.0604	16.3208	1.1128	2014	Bieker et al. 2022	ERR7451426; ERR7446451
EU2-21-29	EU2-21	EUR	EUR	50.19005	15.0604	7.0101	0.9874	2014	Bieker et al. 2022	ERR7446411
EU2-22-1	EU2-22	EUR	EUR	49.41803	17.9615	8.7722	0.9522	2014	Bieker et al. 2022	ERR7446450
EU2-22-10	EU2-22	EUR	EUR	49.41803	17.9615	24.7317	0.9767	2014	Bieker et al. 2022	ERR7446528
EU2-22-13	EU2-22	EUR	EUR	49.41803	17.9615	9.4278	0.9763	2014	Bieker et al. 2022	ERR7446454
EU2-22-15	EU2-22	EUR	EUR	49.41803	17.9615	4.2722	0.9318	2014	Bieker et al. 2022	ERR7446402
EU2-22-18	EU2-22	EUR	EUR	49.41803	17.9615	6.4411	1.0050	2014	Bieker et al. 2022	ERR7446440

EU2-22-19	EU2-22	EUR	EUR	49.41803	17.9615	8.9962	0.9476	2014	Bieker et al. 2022	ERR7446475
EU2-22-22	EU2-22	EUR	EUR	49.41803	17.9615	4.1665	0.9615	2014	Bieker et al. 2022	ERR7446434
EU2-22-24	EU2-22	EUR	EUR	49.41803	17.9615	4.6104	1.0241	2014	Bieker et al. 2022	ERR7446437
EU2-22-25	EU2-22	EUR	EUR	49.41803	17.9615	6.0168	0.9129	2014	Bieker et al. 2022	ERR7446446
EU2-22-26	EU2-22	EUR	EUR	49.41803	17.9615	7.8802	0.9739	2014	Bieker et al. 2022	ERR7446481
EU2-23-10	EU2-23	EUR.W	EUR	50.44297	18.8634	4.6617	1.0162	2014	Bieker et al. 2022	ERR7446445
EU2-23-12	EU2-23	EUR.W	EUR	50.44297	18.8634	0.7851	0.9851	2014	Bieker et al. 2022	ERR7446436
EU2-23-17	EU2-23	EUR.W	EUR	50.44297	18.8634	7.9307	1.0145	2014	Bieker et al. 2022	ERR7446492
EU2-24-1	EU2-24	EUR.ME	EUR	48.48919	21.8062	4.4369	1.1295	2014	Bieker et al. 2022	ERR7448892
EU2-24-10	EU2-24	EUR.ME	EUR	48.48919	21.8062	7.0198	0.9585	2014	Bieker et al. 2022	ERR7446458
EU2-25-14	EU2-25	EUR.ME	EUR	47.9773	23.0443	4.5275	0.9649	2014	Bieker et al. 2022	ERR7446447
EU2-25-15	EU2-25	EUR.W	EUR	47.9773	23.0443	2.2453	0.9677	2014	Bieker et al. 2022	ERR7446441
EU2-26-1	EU2-26	EUR.ME	EUR	46.23696	24.854	3.4625	0.9341	2014	Bieker et al. 2022	ERR7446443
EU2-26-12	EU2-26	EUR.ME	EUR	46.23696	24.854	3.0609	1.1769	2014	Bieker et al. 2022	ERR7448874; ERR7446442
EU2-30-1	EU2-30	EUR.W	EUR	49.86851	23.0118	3.3174	0.9526	2014	Bieker et al. 2022	ERR7446444
EU2-30-12	EU2-30	EUR.W	EUR	49.86851	23.0118	5.6438	0.9525	2014	Bieker et al. 2022	ERR7446457
EU2-31-1	EU2-31	EUR.ME	EUR	45.37402	27.072	6.2095	1.0012	2014	Bieker et al. 2022	ERR7446483
EU2-31-11	EU2-31	EUR.ME	EUR	45.37402	27.072	3.3948	0.9287	2014	Bieker et al. 2022	ERR7446449
EU2-32-12	EU2-32	EUR.ME	EUR	44.16268	28.5098	5.0553	0.9452	2014	Bieker et al. 2022	ERR7446464
EU2-32-13	EU2-32	EUR.ME	EUR	44.16268	28.5098	4.8814	1.1201	2014	Bieker et al. 2022	ERR7448875; ERR7446452

EU2-33-1	EU2-33	EUR.ME	EUR	44.40457	26.1348	4.1748	0.9816	2014	Bieker et al. 2022	ERR7446455
EU2-33-10	EU2-33	EUR.ME	EUR	44.40457	26.1348	4.9777	0.9643	2014	Bieker et al. 2022	ERR7446468
EU2-35-1	EU2-35	EUR.ME	EUR	43.09184	21.938	4.2050	0.9587	2014	Bieker et al. 2022	ERR7446471
EU2-35-14	EU2-35	EUR.ME	EUR	43.09184	21.938	3.5622	0.9866	2014	Bieker et al. 2022	ERR7446469
EU2-36-1	EU2-36	EUR.ME	EUR	43.31519	24.2598	1.2617	1.0470	2014	Bieker et al. 2022	ERR7446456
EU2-36-11	EU2-36	EUR.ME	EUR	43.31519	24.2598	3.9894	0.9995	2014	Bieker et al. 2022	ERR7446470
EU2-37-1	EU2-37	EUR.W	EUR	43.91788	20.7331	3.6060	0.9249	2014	Bieker et al. 2022	ERR7446472
EU2-37-11	EU2-37	EUR	EUR	43.91788	20.7331	3.0739	0.9519	2014	Bieker et al. 2022	ERR7446473
EU2-38-12D	EU2-38	EUR.ME	EUR	45.71531	15.6538	8.1826	0.9676	2014	Bieker et al. 2022	ERR7446511
EU2-38-13D	EU2-38	EUR.ME	EUR	45.71531	15.6538	12.1958	0.9833	2014	Bieker et al. 2022	ERR7446526
FL-26	FL	NAM.S	NAM	30.4063	-83.14	13.3367	1.1432	2013	Bieker et al. 2022	ERR7451287; ERR7446520
FL-9	FL	NAM.S	NAM	30.4063	-83.14	11.2490	1.0084	2013	Bieker et al. 2022	ERR7451432; ERR7446477
FL-A-1	FLA	NAM.S	NAM	25.54179	-80.413	5.9216	1.0107	2009	Bieker et al. 2022	ERR7446494
FL-B-1	FLB	NAM.S	NAM	27.76424	-81.595	0.8935	1.0021	2009	Bieker et al. 2022	ERR7446474
FL-B-2	FLB	NAM.S	NAM	27.76424	-81.595	2.8742	0.9945	2009	Bieker et al. 2022	ERR7446478
FL-D-2018-1	FLD	NAM.S	NAM	27.37437	-80.416	4.1269	0.9944	2009	Bieker et al. 2022	ERR7446489
FL-D-2018-2	FLD	NAM.S	NAM	27.37437	-80.416	4.4383	1.0179	2009	Bieker et al. 2022	ERR7446488
FL-D-2018-3	FLD	NAM.S	NAM	27.37437	-80.416	4.0206	1.0147	2009	Bieker et al. 2022	ERR7446490
FL-E-1	FLE	NAM.S	NAM	29.35716	-81.16	0.9599	0.9893	2009	Bieker et al. 2022	ERR7446479
FL-E-2	FLE	NAM.S	NAM	29.35716	-81.16	0.4387	0.9846	2009	Bieker et al. 2022	ERR7446476

FL-F-1	FL-F	NAM.ME	NAM	30.69589	-86.751	3.7919	1.0308	2009	Bieker et al. 2022	ERR7446486
FL-F-2	FL-F	NAM.ME	NAM	30.69589	-86.751	3.4098	0.9996	2009	Bieker et al. 2022	ERR7446491
FLD-1	FLD	NAM.S	NAM	27.37437	-80.416	13.5614	1.0756	2009	Bieker et al. 2022	ERR3597488
FLD-4	FLD	NAM.S	NAM	27.37437	-80.416	1.2682	1.0723	2009	Bieker et al. 2022	ERR3602084
FLD-5	FLD	NAM.S	NAM	27.37437	-80.416	1.2529	1.0306	2009	Bieker et al. 2022	ERR3597473
FLD-6	FLD	NAM.S	NAM	27.37437	-80.416	1.4214	1.0491	2009	Bieker et al. 2022	ERR3597475
FLD-7	FLD	NAM.S	NAM	27.37437	-80.416	3.6621	1.1355	2009	Bieker et al. 2022	ERR3597480
FLG-7	FLG	NAM.S	NAM	27.08522	-81.795	9.9631	1.0374	2009	Bieker et al. 2022	ERR3597486
FLG-8	FLG	NAM.S	NAM	27.08522	-81.795	10.3597	1.0853	2009	Bieker et al. 2022	ERR3597487
FLH-2018-1	FLH-2018	NAM.S	NAM	29.95931	-82.712	3.1566	1.0225	2009	Bieker et al. 2022	ERR7446487
FLH-2018-2	FLH-2018	NAM.S	NAM	29.95931	-82.712	2.4126	1.0619	2009	Bieker et al. 2022	ERR7446485
FLH-2018-3	FLH-2018	NAM.S	NAM	29.95931	-82.712	3.6050	1.0035	2009	Bieker et al. 2022	ERR7446493
FLH-2018-5	FLH-2018	NAM.S	NAM	29.95931	-82.712	3.4336	1.0452	2009	Bieker et al. 2022	ERR7446496
FLH-2018-6	FLH-2018	NAM.S	NAM	29.95931	-82.712	6.4303	1.0580	2009	Bieker et al. 2022	ERR7446504
FR-5	FR	EUR.ME	EUR	45.67878	4.97993	4.3333	1.1580	2008	Bieker et al. 2022	ERR7446501
FR1-26	FR	EUR.ME	EUR	45.08023	4.75744	3.9832	0.9699	2008	Bieker et al. 2022	ERR7446500
FR2-1	FR	EUR.ME	EUR	45.67878	4.97993	6.0073	1.1671	2008	Bieker et al. 2022	ERR7446508
FR2-7	FR	EUR.ME	EUR	45.67878	4.97993	2.7561	1.2280	2008	Bieker et al. 2022	ERR7446499
FR7-3	FR7	EUR.ME	EUR	47.17582	3.01463	5.8207	1.1015	2008	Bieker et al. 2022	ERR7446507
FR7-6	FR7	EUR.ME	EUR	47.17582	3.01463	2.2410	1.1991	2008	Bieker et al. 2022	ERR7446497

FR8-19	FR8	EUR.ME	EUR	44.21666	4.26401	12.0241	0.9589	2008	Bieker et al. 2022	ERR7446540
FR9-5	FR9	EUR.ME	EUR	44.75302	4.87052	6.5927	1.0966	2008	Bieker et al. 2022	ERR7446513
GA-1	GA	NAM.ME	NAM	31.67867	-83.196	0.8414	0.9963	2009	Bieker et al. 2022	ERR7446495
GA-2	GA	NAM.ME	NAM	31.67867	-83.196	4.2582	0.9978	2009	Bieker et al. 2022	ERR7446502
IA-1	IA	NAM.W	NAM	41.87498	-93.483	5.5944	0.9539	2009	Bieker et al. 2022	ERR7446512
IA-10	IA	NAM.W	NAM	41.87498	-93.483	3.2975	0.9452	2009	Bieker et al. 2022	ERR7446503
IA-2	IA	NAM.W	NAM	41.87498	-93.483	3.7093	0.9659	2009	Bieker et al. 2022	ERR7446506
IA-3	IA	NAM.W	NAM	41.87498	-93.483	3.2523	0.9545	2009	Bieker et al. 2022	ERR7446505
IA-4	IA	NAM.W	NAM	41.87498	-93.483	5.0982	0.9922	2009	Bieker et al. 2022	ERR7446516
IA-5	IA	NAM.W	NAM	41.87498	-93.483	2.9989	0.9653	2009	Bieker et al. 2022	ERR7446509
IA-6	IA	NAM.W	NAM	41.87498	-93.483	9.9222	0.9963	2009	Bieker et al. 2022	ERR7446544
IA-7	IA	NAM.W	NAM	41.87498	-93.483	4.6000	0.9115	2009	Bieker et al. 2022	ERR7446522
IA-8	IA	NAM.W	NAM	41.87498	-93.483	3.6117	0.9794	2009	Bieker et al. 2022	ERR7446517
IA-9	IA	NAM.W	NAM	41.87498	-93.483	4.0651	0.9519	2009	Bieker et al. 2022	ERR7446518
IL-1	IL	NAM.ME	NAM	39.23775	-88.151	3.5282	0.9368	2009	Bieker et al. 2022	ERR7446515
IL-2	IL	NAM.ME	NAM	39.23775	-88.151	13.6389	0.9802	2009	Bieker et al. 2022	ERR7446653
IW-10	IW	NAM.W	NAM	42.67797	-96.502	18.7807	1.1914	2013	Bieker et al. 2022	ERR7451325; ERR7446648
IW-11	IW	NAM.W	NAM	42.67797	-96.502	4.1507	0.9730	2013	Bieker et al. 2022	ERR7446524
K12-1	K	EUR.ME	EUR	45.25472	19.9123	7.6658	0.9882	2017	Bieker et al. 2022	ERR7446537
K13-1	K	EUR	EUR	45.25472	19.9123	13.9745	0.9809	2017	Bieker et al. 2022	ERR7446702

K16-1	K	EUR	EUR	45.25472	19.9123	12.9292	0.8971	2017	Bieker et al. 2022	ERR7446633
K2-1	K	EUR	EUR	45.25472	19.9123	8.4464	0.9540	2017	Bieker et al. 2022	ERR7446538
KY-14	KY	NAM.ME	NAM	38.62588	-85.007	12.3392	1.0120	2013	Bieker et al. 2022	ERR7446641
KY-29	KY	NAM.ME	NAM	38.62588	-85.007	9.8306	0.9731	2013	Bieker et al. 2022	ERR7446590
MA-A-1	MA-A	NAM.E	NAM	42.31109	-71.161	4.8235	0.9672	2009	Bieker et al. 2022	ERR7446529
MA-A-10	MA-A	NAM.E	NAM	42.31109	-71.161	6.0982	0.9245	2009	Bieker et al. 2022	ERR7446531
MA-A-2	MA-A	NAM.E	NAM	42.31109	-71.161	4.0472	0.9578	2009	Bieker et al. 2022	ERR7446527
MA-A-3	MA-A	NAM.E	NAM	42.31109	-71.161	7.7527	1.0113	2009	Bieker et al. 2022	ERR7446588
MA-A-4	MA-A	NAM.E	NAM	42.31109	-71.161	6.4091	0.9485	2009	Bieker et al. 2022	ERR7446543
MA-A-5	MA-A	NAM.E	NAM	42.31109	-71.161	6.2008	0.9328	2009	Bieker et al. 2022	ERR7446546
MA-A-6	MA-A	NAM.E	NAM	42.31109	-71.161	5.2797	0.9307	2009	Bieker et al. 2022	ERR7446533
MA-A-7	MA-A	NAM.E	NAM	42.31109	-71.161	5.2108	1.0136	2009	Bieker et al. 2022	ERR7446534
MA-A-8	MA-A	NAM.E	NAM	42.31109	-71.161	7.6604	0.9536	2009	Bieker et al. 2022	ERR7446623
MA-A-9	MA-A	NAM.E	NAM	42.31109	-71.161	5.1472	0.9501	2009	Bieker et al. 2022	ERR7446536
ME-C-1	ME-C	NAM.E	NAM	44.01332	-70.069	8.7372	0.8921	2009	Bieker et al. 2022	ERR7446639
ME-C-2	ME-C	NAM.E	NAM	44.01332	-70.069	10.4276	0.9416	2009	Bieker et al. 2022	ERR7447157
ME-E-1	ME-E	NAM.E	NAM	45.15784	-67.289	2.5485	0.9457	2009	Bieker et al. 2022	ERR7446539
ME-E-2	ME-E	NAM.E	NAM	45.15784	-67.289	14.5752	0.9583	2009	Bieker et al. 2022	ERR7448117
MI-2	MI	NAM.ME	NAM	46.35814	-84.881	0.9342	1.0652	2013	Bieker et al. 2022	ERR7446542
MI-3	MI	NAM.ME	NAM	46.35814	-84.881	3.6440	1.6317	2013	Bieker et al. 2022	ERR7450296; ERR7446545

MI-B-2019-1	MI-B-2019	NAM.ME	NAM	43.15405	-84.504	3.2586	0.9443	2009	Bieker et al. 2022	ERR7446611
MI-B-2019-2	MI-B-2019	NAM.ME	NAM	43.15405	-84.504	2.9574	0.9627	2009	Bieker et al. 2022	ERR7446610
MN2-1	MN2	NAM.ME	NAM	44.44716	-79.804	7.7749	0.9917	2013	Bieker et al. 2022	ERR7447078
MN2-3	MN2	NAM.ME	NAM	44.44716	-79.804	13.4924	0.9777	2013	Bieker et al. 2022	ERR7448167
MO-A-2019-1	MO-A-2019	NAM.W	NAM	38.96843	-93.879	2.7041	0.9194	2009	Bieker et al. 2022	ERR7446608
MO-A-2019-2	MO-A-2019	NAM.W	NAM	38.96843	-93.879	3.8987	0.9219	2009	Bieker et al. 2022	ERR7446654
MO-B-2018-3	MO-B-2018	NAM.ME	NAM	38.38635	-90.259	6.4414	0.9627	2009	Bieker et al. 2022	ERR7446703
MO-B-2018-4	MO-B-2018	NAM.ME	NAM	38.38635	-90.259	4.5954	1.0263	2009	Bieker et al. 2022	ERR7446665
MO-B-2018-5	MO-B-2018	NAM.ME	NAM	38.38635	-90.259	7.9265	1.0148	2009	Bieker et al. 2022	ERR7447534
MO-B-2018-6	MO-B-2018	NAM.ME	NAM	38.38635	-90.259	4.0190	0.9691	2009	Bieker et al. 2022	ERR7446679
MO-B-2018-7	MO-B-2018	NAM.ME	NAM	38.38635	-90.259	3.3254	0.9741	2009	Bieker et al. 2022	ERR7446666
MO-E-2019-1	MO-E-2019	NAM.ME	NAM	36.51766	-90.249	7.0992	0.9525	2009	Bieker et al. 2022	ERR7448103
MO-E-2019-2	MO-E-2019	NAM.ME	NAM	36.51766	-90.249	3.6192	0.9228	2009	Bieker et al. 2022	ERR7446748
MO-G-1	MO-G	NAM.W	NAM	36.66569	-94.347	0.6099	1.0188	2009	Bieker et al. 2022	ERR7446640
MO-G-2	MO-G	NAM.W	NAM	36.66569	-94.347	4.1773	0.9825	2009	Bieker et al. 2022	ERR7446722
MOB-4	MO-B-2018	NAM.ME	NAM	38.38635	-90.259	1.6081	1.0983	2009	Bieker et al. 2022	ERR3602086
MOB-7	MO-B-2018	NAM.ME	NAM	38.38635	-90.259	1.9132	1.0265	2009	Bieker et al. 2022	ERR3602089
MOB-8	MO-B-2018	NAM.ME	NAM	38.38635	-90.259	7.2387	1.0883	2009	Bieker et al. 2022	ERR3597485
MP-16	MP	NAM.ME	NAM	31.20781	-89.066	12.6345	1.0097	2013	Bieker et al. 2022	ERR7451248; ERR7447519
MP-18	MP	NAM.ME	NAM	31.20781	-89.066	15.1605	1.0098	2013	Bieker et al. 2022	ERR7451347; ERR7448094

MS-1	MS	NAM.E	NAM	42.0883	-72.096	12.8406	1.0085	2013	Bieker et al. 2022	ERR7448186
MS-3	MS	NAM.E	NAM	42.0883	-72.096	8.2205	0.9596	2013	Bieker et al. 2022	ERR7447941
NB-A-1	NB-A	NAM.ME	NAM	46.03306	-65.041	9.3975	0.9366	2009	Bieker et al. 2022	ERR7448100
NB-A-2	NB-A	NAM.ME	NAM	46.03306	-65.041	13.1043	0.9726	2009	Bieker et al. 2022	ERR7448220
NC-10-F	NC	NAM.ME	NAM	35.60699	-83.022	3.5650	0.9892	2013	Bieker et al. 2022	ERR7446716
NC-9D	NC	NAM.ME	NAM	35.60699	-83.022	9.5657	1.0252	2013	Bieker et al. 2022	ERR7448102
NH-A-1	NH-A	NAM.E	NAM	42.89069	-72.528	6.5310	1.0184	2009	Bieker et al. 2022	ERR7447211
NH-E-1	NH-E	NAM.E	NAM	43.1863	-71.14	7.7417	0.9858	2009	Bieker et al. 2022	ERR7447499
NH-E-2	NH-E	NAM.E	NAM	43.1863	-71.14	6.2738	0.9523	2009	Bieker et al. 2022	ERR7447176
NJ-C-1	NJ-C	NAM.E	NAM	39.62068	-75.506	7.2094	0.9867	2009	Bieker et al. 2022	ERR7448053
NJ-C-2	NJ-C	NAM.E	NAM	39.62068	-75.506	7.6935	0.9548	2009	Bieker et al. 2022	ERR7447502
NJ-C-3	NJ-C	NAM.E	NAM	39.62068	-75.506	5.2191	1.0000	2009	Bieker et al. 2022	ERR7447174
NJ-C-4	NJ-C	NAM.E	NAM	39.62068	-75.506	5.3718	0.9768	2009	Bieker et al. 2022	ERR7447760
NJ-C-6	NJ-C	NAM.E	NAM	39.62068	-75.506	11.3707	0.9923	2009	Bieker et al. 2022	ERR7448262
NJ-C-7	NJ-C	NAM.E	NAM	39.62068	-75.506	7.7610	1.0154	2009	Bieker et al. 2022	ERR7448182
NJ-C-8	NJ-C	NAM.E	NAM	39.62068	-75.506	3.2436	0.9320	2009	Bieker et al. 2022	ERR7447517
NJ-G-1	NJ-G	NAM.E	NAM	40.77276	-74.504	2.1328	0.9274	2009	Bieker et al. 2022	ERR7447527
NJ-G-2	NJ-G	NAM.E	NAM	40.77276	-74.504	6.3335	0.9525	2009	Bieker et al. 2022	ERR7448239
NS-A-2018-1	NSA	NAM.E	NAM	44.9088	-65.177	3.1109	0.8764	2009	Bieker et al. 2022	ERR7447865
NS-A-2018-2	NSA	NAM.E	NAM	44.9088	-65.177	2.9942	0.9323	2009	Bieker et al. 2022	ERR7447940

NS-A-2018-3	NSA	NAM.E	NAM	44.9088	-65.177	4.0830	0.9257	2009	Bieker et al. 2022	ERR7448012
NSA-1	NSA	NAM.E	NAM	44.9088	-65.177	1.1376	1.0655	2009	Bieker et al. 2022	ERR3597474
NSA-3	NSA	NAM.E	NAM	44.9088	-65.177	0.9488	1.1834	2009	Bieker et al. 2022	ERR3597476
NSA-6	NSA	NAM.E	NAM	44.9088	-65.177	6.8288	0.8991	2009	Bieker et al. 2022	ERR3597483
NSA-7	NSA	NAM.E	NAM	44.9088	-65.177	0.9618	1.0000	2009	Bieker et al. 2022	ERR3602088
NSA-8	NSA	NAM.E	NAM	44.9088	-65.177	1.2159	0.8917	2009	Bieker et al. 2022	ERR3602090
NSB-6	NSB	NAM.E	NAM	44.75472	-63.672	5.7601	0.9622	2009	Bieker et al. 2022	ERR3597482
NSB-7	NSB	NAM.E	NAM	44.75472	-63.672	0.8587	0.9958	2009	Bieker et al. 2022	ERR3597472
OH-1	OH	NAM.ME	NAM	40.48763	-82.727	3.8546	0.9681	2013	Bieker et al. 2022	ERR7447938
OH-6	OH	NAM.ME	NAM	40.48763	-82.727	13.4975	0.9653	2013	Bieker et al. 2022	ERR7448731
OH-B-1	OH-B	NAM.ME	NAM	38.90957	-82.728	3.4613	0.9543	2009	Bieker et al. 2022	ERR7447947
OH-B-2	OH-B	NAM.ME	NAM	38.90957	-82.728	11.4776	0.9653	2009	Bieker et al. 2022	ERR7448288
ON3-7	ON3	NAM.ME	NAM	45.32971	-74.893	2.3187	1.0067	2013	Bieker et al. 2022	ERR7447993
ON3-8	ON3	NAM.E	NAM	45.32971	-74.893	8.5030	0.9787	2013	Bieker et al. 2022	ERR7448263
PA-1	PA	NAM.ME	NAM	40.96589	-78.175	4.5162	1.1286	2013	Bieker et al. 2022	ERR7448022
PA-5	PA	NAM.ME	NAM	40.96589	-78.175	11.4825	0.9404	2013	Bieker et al. 2022	ERR7448510
PA-D-1	PA-D	NAM.E	NAM	41.31407	-75.54	7.8595	0.9669	2009	Bieker et al. 2022	ERR7448214
PA-D-2	PA-D	NAM.E	NAM	41.31407	-75.54	11.6978	0.9909	2009	Bieker et al. 2022	ERR7448733
PA-E-1	PA-E	NAM.E	NAM	40.20361	-75.906	12.8023	0.9946	2009	Bieker et al. 2022	ERR7448742
PA-E-2	PA-E	NAM.E	NAM	40.20361	-75.906	14.1769	1.0022	2009	Bieker et al. 2022	ERR7448755

PA-F-1	PA-F	NAM.ME	NAM	41.8322	-80.116	21.0436	0.9833	2009	Bieker et al. 2022	ERR7448829
PA-F-2	PA-F	NAM.ME	NAM	41.8322	-80.116	10.7888	0.9728	2009	Bieker et al. 2022	ERR7448750
pop2-1	pop2	EUR.ME	EUR	45.40917	-0.5294	1.7380	0.9277	2019	Bieker et al. 2022	ERR7451245
pop2-2	pop2	EUR.ME	EUR	45.40917	-0.5294	2.9742	1.0134	2019	Bieker et al. 2022	ERR7451289
pop2-3	pop2	EUR.ME	EUR	45.40917	-0.5294	3.4648	0.9573	2019	Bieker et al. 2022	ERR7451315
pop3-1	pop3	EUR.ME	EUR	45.47806	-0.3731	2.8549	0.9634	2019	Bieker et al. 2022	ERR7451336
pop3-2	pop3	EUR.ME	EUR	45.47806	-0.3731	6.7457	0.9609	2019	Bieker et al. 2022	ERR7451586
pop3-3	pop3	EUR.ME	EUR	45.47806	-0.3731	4.0197	0.9600	2019	Bieker et al. 2022	ERR7451499
QC-2-1	QC-2	NAM.E	NAM	46.88667	-70.868	15.6584	1.0271	2013	Bieker et al. 2022	ERR7451329; ERR7448740
QC-2-17	QC-2	NAM.E	NAM	46.88667	-70.868	17.6918	0.9922	2013	Bieker et al. 2022	ERR7448790
QC-2-19	QC-2	NAM.E	NAM	46.88667	-70.868	11.3993	0.9803	2013	Bieker et al. 2022	ERR7451307; ERR7448215
QC-2-20	QC-2	NAM.E	NAM	46.88667	-70.868	11.3528	1.0596	2013	Bieker et al. 2022	ERR7451423; ERR7448253
QC-2-22	QC-2	NAM.E	NAM	46.88667	-70.868	10.3134	1.0286	2013	Bieker et al. 2022	ERR7451252; ERR7448286
QC-2-25	QC-2	NAM.E	NAM	46.88667	-70.868	13.9760	0.9929	2013	Bieker et al. 2022	ERR7451297; ERR7448744
QC-2-26	QC-2	NAM.E	NAM	46.88667	-70.868	12.5079	1.0792	2013	Bieker et al. 2022	ERR7451285; ERR7448754
QC-2-28	QC-2	NAM.ME	NAM	46.88667	-70.868	13.7966	1.0342	2013	Bieker et al. 2022	ERR7451417; ERR7448737
QC-2-29	QC-2	NAM.E	NAM	46.88667	-70.868	18.2830	1.0502	2013	Bieker et al. 2022	ERR7451446; ERR7448803
QC-2-30	QC-2	NAM.E	NAM	46.88667	-70.868	26.2481	0.9974	2013	Bieker et al. 2022	ERR7451480; ERR7451456
QC3-20	QC-3	NAM.E	NAM	47.67876	-69.022	15.8518	0.9958	2013	Bieker et al. 2022	ERR7448889
RI-B-1	RI-B	NAM.E	NAM	41.48481	-71.558	6.0052	0.9360	2009	Bieker et al. 2022	ERR7448734

RI-B-2	RI-B	NAM.E	NAM	41.48481	-71.558	14.3824	0.9371	2009	Bieker et al. 2022	ERR7448847
SC-1	SC	NAM.E	NAM	34.79252	-80.415	17.0874	0.9987	2009	Bieker et al. 2022	ERR7451327
SC-2	SC	NAM.ME	NAM	34.79252	-80.415	18.4031	0.9924	2009	Bieker et al. 2022	ERR7451414
SC-K-1	SC-K	NAM.ME	NAM	34.22524	-81.343	4.3607	1.0264	2013	Bieker et al. 2022	ERR7448736
SC-K-2	SC-K	NAM.ME	NAM	34.22524	-81.343	7.6215	1.0367	2013	Bieker et al. 2022	ERR7448761
StGalmier-1	StGalmier	EUR.ME	EUR	45.59	4.4	14.1354	0.9911	2017	Bieker et al. 2022	ERR7451338
StGalmier-11	StGalmier	EUR.ME	EUR	45.59	4.4	9.6901	0.9406	2017	Bieker et al. 2022	ERR7448872
StGalmier-12	StGalmier	EUR.ME	EUR	45.59	4.4	7.1991	0.9848	2017	Bieker et al. 2022	ERR7448826
StGalmier-16	StGalmier	EUR.ME	EUR	45.59	4.4	10.0433	0.9659	2017	Bieker et al. 2022	ERR7448886
StGalmier-17	StGalmier	EUR.ME	EUR	45.59	4.4	12.9315	1.0225	2017	Bieker et al. 2022	ERR7448881
StGalmier-2	StGalmier	EUR.ME	EUR	45.59	4.4	12.9776	0.9387	2017	Bieker et al. 2022	ERR7451425
StGalmier-21	StGalmier	EUR.ME	EUR	45.59	4.4	6.3308	0.9525	2017	Bieker et al. 2022	ERR7448785
StGalmier-22	StGalmier	EUR.ME	EUR	45.59	4.4	10.0684	0.9722	2017	Bieker et al. 2022	ERR7448878
StGalmier-6	StGalmier	EUR.ME	EUR	45.59	4.4	13.6943	0.9648	2017	Bieker et al. 2022	ERR7451437
TN-1	TN	NAM.ME	NAM	36.11943	-85.059	4.4479	0.9572	2009	Bieker et al. 2022	ERR7448786
TN-2019-1	TN	NAM.ME	NAM	36.11943	-85.059	2.6949	0.9684	2009	Bieker et al. 2022	ERR7448793
TRH-5	TRH	EUR.ME	EUR	58.08278	6.75278	2.2637	0.9819	2007	Bieker et al. 2022	ERR7460255
TRH-6	TRH	EUR	EUR	58.08278	6.75278	1.7922	0.9442	2007	Bieker et al. 2022	ERR7460286
TRQB-1	TRQB	NAM.E	NAM	45.5	-72.5	9.0505	1.0089	2013	Bieker et al. 2022	ERR7451343
TRQB-10	TRQB	NAM.E	NAM	45.5	-72.5	10.9787	1.0731	2013	Bieker et al. 2022	ERR7451326

TRQB-2	TRQB	NAM.E	NAM	45.5	-72.5	12.1401	1.0382	2013	Bieker et al. 2022	ERR7451441
TRQB-4	TRQB	NAM.E	NAM	45.5	-72.5	8.1634	0.9938	2013	Bieker et al. 2022	ERR7451278
TRQB-7	TRQB	NAM.E	NAM	45.5	-72.5	7.8946	0.9639	2013	Bieker et al. 2022	ERR7450221
TRQB-8	TRQB	NAM.E	NAM	45.5	-72.5	11.7018	1.0032	2013	Bieker et al. 2022	ERR7451447
TRQB-9	TRQB	NAM.E	NAM	45.5	-72.5	10.0110	0.9640	2013	Bieker et al. 2022	ERR7451419
WI-1	WI	NAM.ME	NAM	44.87934	-89.424	15.3697	1.0095	2013	Bieker et al. 2022	ERR7451468
WI-3	WI	NAM.ME	NAM	44.87934	-89.424	6.7306	0.9665	2013	Bieker et al. 2022	ERR7451282
WU-15	WU	EUR	EUR	47.98833	16.2831	6.2109	0.9683	2010	Bieker et al. 2022	ERR7451424
WU-16	WU	EUR.ME	EUR	47.99444	16.3294	2.9881	0.9224	2016	Bieker et al. 2022	ERR7451249
WU-17	WU	EUR.ME	EUR	48.20028	16.455	2.9390	0.9274	2013	Bieker et al. 2022	ERR7451269

Rows shaded green indicate samples excluded from downstream analyses after applying a genome size standard deviation (SD)-based outlier filter.

Tables S2: Summary statistics of rRNA and TE annotations in the common ragweed (*Ambrosia artemisiifolia* L.) genome, by family. For each rRNA/TE family, the table reports the number of annotated sites, total annotated length (Mbp), mean element length (mean ± standard error [SE], base pairs), and mean distance to the nearest gene (mean ± SE, Mbp).

TE and rRNA	Number of sites	Total length (Mbp)	Mean_length ± SE (bp)	Mean distance to nearest gene ± SE (Mbp)
TIR/PIF_Harbinger	22782	7.3997	325.49 ± 2.63	0.0489 ± 0.001
Helitron	473277	173.8820	371.29 ± 0.83	0.0234 ± 0.0001
Low_complexity	22686	1.1171	49.31 ± 0.19	0.0173 ± 0.0004
LTR/unknown	201031	128.1834	637.84 ± 2.33	0.0404 ± 0.0001
LTR/Gypsy	105311	134.4367	1276.75 ± 6.17	0.0447 ± 0.0003
TIR/hAT	87681	29.3837	335.31 ± 2.09	0.0169 ± 0.0002
LTR/Copia	154880	185.3814	1197.04 ± 4.73	0.053 ± 0.0003

TIR/Mutator	133168	51.3472	385.85 ± 2.57	0.1923 ± 0.0009
TIR/CACTA	44239	14.7256	333.98 ± 2.15	0.0254 ± 0.0004
TIR/Tc1_Mariner	4859	1.2810	265.07 ± 3.84	0.0156 ± 0.0005
Simple_repeat	131708	5.9165	44.99 ± 0.41	0.0176 ± 0.0002
8s_rRNA	3324	0.3823	115 ± 0.01	0.2911 ± 0.0026
18s_rRNA	9	0.0149	1652.22 ± 47.04	0.03 ± 0.0078
28s_rRNA	10	0.0523	5232.5 ± 285.5	0.0263 ± 0.0065

Tables S3: Descriptions of the 19 bioclimatic variables (BIO1–BIO19) from the WorldClim database used to characterise the climatic niche of common ragweed (*Ambrosia artemisiifolia* L.) sampling sites in this study, with the unit of measurement for each variable.

**Bioclimatic Variable**	**Description**	**Unit**
BIO1	Annual Mean Temperature	°C
BIO2	Mean Diurnal Range (Mean of monthly (max temp - min temp))	°C
BIO3	Isothermality (BIO2/BIO7) (*100)	Dimensionless (%)
BIO4	Temperature Seasonality (Standard deviation *100)	°C
BIO5	Max Temperature of Warmest Month	°C
BIO6	Min Temperature of Coldest Month	°C
BIO7	Temperature Annual Range (BIO5-BIO6)	°C
BIO8	Mean Temperature of Wettest Quarter	°C
BIO9	Mean Temperature of Driest Quarter	°C
BIO10	Mean Temperature of Warmest Quarter	°C
BIO11	Mean Temperature of Coldest Quarter	°C
BIO12	Annual Precipitation	mm
BIO13	Precipitation of Wettest Month	mm

BIO14	Precipitation of Driest Month	mm
BIO15	Precipitation Seasonality (Coefficient of Variation)	Dimensionless (%)
BIO16	Precipitation of Wettest Quarter	mm
BIO17	Precipitation of Driest Quarter	mm
BIO18	Precipitation of Warmest Quarter	mm
BIO19	Precipitation of Coldest Quarter	mm

Tables S4: Flow cytometry-based genome size measurements for 40 common ragweed (*Ambrosia artemisiifolia* L.) samples from the native North American range and the invasive ranges of Europe and Australia. For each sample, the table reports the source population, geographic range, diploid (2C) DNA content in picograms (pg) and gigabase pairs (Gbp), and the derived haploid (1C) genome size (Gbp). Picogram values were converted to Gbp using a conversion factor of 1 pg DNA = 0.978 Gbp.

Sample	Population	Range	2C (pg)	2C (Gbp)	1C (Gbp)
NSA-1-4	NSA	North America	2.2207	2.1718	1.0859
NC-10-2	NC	North America	2.3287	2.2774	1.1387
OH-6-6	OH	North America	2.2631	2.2133	1.1067
NSA-13-2	NSA	North America	2.444	2.3903	1.1951
OH-2-4	OH	North America	2.2802	2.23	1.115
AA5-2-1	AA5	North America	2.2484	2.199	1.0995
KY-1-1	KY	North America	2.3922	2.3396	1.1698
TRQB-3-1	TRQB	North America	2.3395	2.288	1.144
CTH-2-1	CT-H	North America	2.3664	2.3144	1.1572
IA-7-1	IA	North America	2.1761	2.1282	1.0641
MI-3-4	MI	North America	2.198	2.1497	1.0748
MP-4-2	MP	North America	2.3508	2.2991	1.1495
OH-10-1	OH	North America	2.2398	2.1905	1.0953

PA-5-2	PA	North America	2.3869	2.3344	1.1672
TN-7-3	TN	North America	2.1832	2.1352	1.0676
NJC-11-1	NJC	North America	2.2328	2.1836	1.0918
EU38-13-1	EU2-38	Europe	2.2797	2.2296	1.1148
EU10-6-4	EU2-10	Europe	2.4011	2.3483	1.1741
EU37-4-4	EU2-37	Europe	2.2467	2.1973	1.0986
EU37-8-2	EU2-37	Europe	2.2679	2.2181	1.109
EU10-10-4	EU2-10	Europe	2.3978	2.345	1.1725
EU10-3-1	EU2-10	Europe	2.4796	2.425	1.2125
AU15-4-1	AU15	Australia	2.7173	2.6575	1.3288
AU26-2-3	AU26	Australia	2.5034	2.4484	1.2242
AU23-3-1	AU23	Australia	2.6446	2.5864	1.2932
AU35-1-2	AU35	Australia	2.3213	2.2702	1.1351
QLD4-8-2	QLD4	Australia	2.3407	2.2892	1.1446
AU9-6-6	AU9	Australia	2.6075	2.5501	1.275
AU1-1-2	AU15	Australia	2.6651	2.6064	1.3032
AU5-4-2	AU5	Australia	2.66	2.6015	1.3008
AU9-2-5	AU9	Australia	2.5703	2.5137	1.2569
AU9-9-3	AU9	Australia	2.6157	2.5582	1.2791
AU15-2-3	AU15	Australia	2.6003	2.5431	1.2716
AU15-6-4	AU15	Australia	2.5979	2.5408	1.2704
AU18-2-5	AU18	Australia	2.6085	2.5511	1.2755
AU18-3-1	AU18	Australia	2.6693	2.6106	1.3053
AU23-7-5	AU23	Australia	2.4133	2.3602	1.1801
AU26-5-6	AU26	Australia	2.4978	2.4428	1.2214
AU30-3-4	AU30	Australia	2.4885	2.4337	1.2169

QLD5-5-5	QLD5	Australia	2.3424	2.2909	1.1454

Tables S5: Pairwise comparisons of genome size between the invasive ranges (Australia, AUS; Europe, EUR) and native North American populations of common ragweed (*Ambrosia artemisiifolia* L.), from a linear mixed-effects model with geographic locations (under comparisons), genetic principal components (PC1, PC2) as a fixed effect and population as a random intercept. North American populations are grouped into four spatio-genetic clusters: mid-east (NAM.ME), south (NAM.S), west (NAM.W), and east (NAM.E). For each pairwise comparison the table reports the model estimate, standard error (SE), degrees of freedom (df), t-ratio, and P-value. Significance codes: ***P < 0.001, **P < 0.01, *P < 0.05, ns = not significant.

Comparison	Estimate	SE	df	t.ratio	p.value	Sig.
EUR vs NAM.W	0.0315	0.0194	52.48	1.63	1	ns
EUR vs NAM.ME	0.0124	0.013	83.88	0.95	0.343	ns
EUR vs NAM.E	0.028	0.0146	80.53	1.91	0.06	ns
EUR vs NAM.S	–0.0342	0.0221	70.18	–1.54	0.127	ns
AUS vs NAM.W	0.1437	0.0149	31.72	9.67	5.68 x 10^{-11}	***
AUS vs NAM.ME	0.1171	0.0125	37.88	9.41	1.87 x 10^{-11}	***
AUS vs NAM.E	0.1402	0.0139	31.1	10.12	2.33 x 10^{-11}	***
AUS vs NAM.S	0.0829	0.0128	34.35	6.47	2.02 x 10^{-7}	***

Tables S6: Analysis of variance (ANOVA) results from linear mixed-effects models testing the effect of geographic range, and post-hoc pairwise comparisons, on TE and rRNA family abundance in common ragweed (*Ambrosia artemisiifolia* L.). Models included geographic range and genetic principal components (PC1, PC2) as fixed effects, with population as a random effect. The upper section reports, for each TE/rRNA family, the F-statistic, numerator degrees of freedom (Df), denominator degrees of freedom (Df.res), raw P-value, and false discovery rate (FDR)-adjusted P-value for the effect of range, with significance codes for both. The lower section reports post-hoc pairwise contrasts between ranges - Australia (AUS), Europe (EUR), and native North America (NAM) - including the estimate, SE, degrees of freedom (df), t-ratio, and P-value for each contrast. Significance codes: ***P < 0.001, **P < 0.01, *P < 0.05, ns = not significant.

TEs and rRNAs	Effect	F	Df	Df.res	p_value	FDR	Sig	FDR_sig

LTR/Copia	range	38.18	2	134.28	7.47×10^{-14}	1.05×10^{-12}	***	***
Helitron	range	4.03	2	155.03	0.0197	0.0229	*	*
LTR/Gypsy	range	27.03	2	150.99	9.25×10^{-11}	4.32×10^{-10}	***	***
LTR/unknown	range	19.24	2	155.3	3.42×10^{-8}	1.2×10^{-7}	***	***
TIR/Mutator	range	32.01	2	130.43	4.91×10^{-12}	3.43×10^{-11}	***	***
TIR/hAT	range	0.76	2	120.52	0.4696	0.4696	ns	ns
TIR/CACTA	range	4.25	2	155.66	0.016	0.0204	*	*
TIR/PIF_Harbinger	range	2.1	2	139.25	0.1265	0.1362	ns	ns
TIR/Tc1_Mariner	range	4.96	2	148.26	0.0082	0.0128	**	*
Low_complexity	range	7.08	2	157.38	0.0011	0.002	**	**
Simple_repeat	range	8.42	2	141.4	0.0004	0.0007	***	***
8s_rRNA	range	4.28	2	126.58	0.0159	0.0204	*	*
18s_rRNA	range	19.28	2	124.78	5.04×10^{-8}	1.41×10^{-7}	***	***
28s_rRNA	range	17.72	2	128.54	1.60×10^{-7}	3.73×10^{-7}	***	***

TEs and rRNAs	contrast	estimate	SE	df	t.ratio	p_value	Sig
LTR/Copia	AUS - EUR	36.9322	5.317	144.0035	6.9461	3.59×10^{-10}	***
	AUS - NAM	44.5185	5.163	141.0133	8.6226	7.24×10^{-14}	***
	EUR - NAM	7.5863	2.5423	126.2924	2.984	0.0095	**
Helitron	AUS - EUR	5.9432	3.1559	161.8721	1.8832	0.1468	ns
	AUS - NAM	1.9478	3.076	157.3124	0.6332	0.8021	ns
	EUR - NAM	-3.9955	1.5448	150.6326	-2.5865	0.0285	*
LTR/Gypsy	AUS - EUR	32.0547	5.7712	161.1381	5.5542	3.38×10^{-7}	***
	AUS - NAM	40.3526	5.6192	156.9374	7.1812	7.82×10^{-11}	***
	EUR - NAM	8.2979	2.8121	143.1265	2.9508	0.0103	*
LTR/unknown	AUS - EUR	18.847	3.0497	161.8466	6.1799	1.50×10^{-8}	***

	AUS - NAM	16.8926	2.9727	157.2636	5.6825	1.88×10^{-7}	***
	EUR - NAM	-1.9544	1.4931	151.2151	-1.3089	0.3924	ns
TIR/Mutator	AUS - EUR	12.6649	1.9899	138.6159	6.3646	8.00×10^{-9}	***
	AUS - NAM	15.248	1.9314	135.8364	7.8946	2.65×10^{-12}	***
	EUR - NAM	2.5832	0.9487	123.7798	2.7228	0.0201	*
TIR/hAT	AUS - EUR	1.3098	1.0823	124.35	1.2101	0.4494	ns
	AUS - NAM	1.2399	1.0496	122.0617	1.1813	0.4666	ns
	EUR - NAM	-0.0699	0.5136	117.9299	-0.1361	0.9898	ns
TIR/CACTA	AUS - EUR	0.6456	0.2426	161.7965	2.6614	0.0232	*
	AUS - NAM	0.4173	0.2365	157.1822	1.7647	0.1849	ns
	EUR - NAM	-0.2283	0.1188	152.0252	-1.9215	0.1361	ns
TIR/PIF_Harbinger	AUS - EUR	0.2292	0.1699	150.5173	1.3495	0.3702	ns
	AUS - NAM	0.3164	0.165	147.2246	1.9172	0.1374	ns
	EUR - NAM	0.0872	0.0816	129.9767	1.0686	0.5353	ns
TIR/Tc1_Mariner	AUS - EUR	0.0331	0.033	159.598	1.0034	0.5758	ns
	AUS - NAM	-0.0173	0.0321	155.6329	-0.54	0.8517	ns
	EUR - NAM	-0.0505	0.016	139.1901	-3.1487	0.0057	**
Low_complexity	AUS - EUR	-0.0134	0.0255	161.3113	-0.5247	0.8594	ns
	AUS - NAM	-0.056	0.0249	156.5534	-2.2502	0.0661	ns
	EUR - NAM	-0.0426	0.0125	156.2153	-3.4059	0.0024	**
Simple_repeat	AUS - EUR	0.7387	0.1877	153.0841	3.9354	0.0004	***
	AUS - NAM	0.5321	0.1824	149.647	2.917	0.0113	*
	EUR - NAM	-0.2065	0.0904	131.7991	-2.2851	0.0614	ns
8s_rRNA	AUS - EUR	0.003	0.0408	133.0891	0.0733	0.997	ns
	AUS - NAM	0.0567	0.0396	130.5065	1.4325	0.3273	ns

	EUR - NAM	0.0537	0.0194	121.4426	2.7676	0.0178	*
18s_rRNA	AUS - EUR	1.4617	0.3324	130.4871	4.3974	6.66×10^{-5}	***
	AUS - NAM	1.9145	0.3225	127.9933	5.9371	7.66×10^{-8}	***
	EUR - NAM	0.4528	0.158	120.3861	2.866	0.0135	*
28s_rRNA	AUS - EUR	3.2618	0.9466	135.9223	3.4459	0.0022	**
	AUS - NAM	4.8848	0.9186	133.2406	5.3177	1.29×10^{-6}	***
	EUR - NAM	1.6231	0.4508	122.6201	3.6007	0.0013	**

Tables S7: Linear mixed-effects model results testing the effect of each TE and rRNA family's abundance on genome size in common ragweed (*Ambrosia artemisiifolia* L.), with genetic principal components (PC1, PC2) included as covariates and population as a random effect. For each TE/rRNA family, the table reports the F-value, raw P-value, false discovery rate (FDR)-adjusted P-value, and significance codes for both. Significance codes: ***P < 0.001, **P < 0.01, *P < 0.05, ns = not significant.

TEs and rRNAs	F_value	p_value	Sig.	FDR	FDR_sig.
LTR/Copia	861.1793	2.25×10^{-102}	***	3.15×10^{-101}	***
Helitron	130.3308	2.13×10^{-24}	***	7.44×10^{-24}	***
LTR/Gypsy	252.3147	1.78×10^{-44}	***	1.25×10^{-43}	***
LTR/unknown	0.91862	0.339	ns	0.365	ns
TIR/Mutator	124.3392	4.57×10^{-25}	***	2.13×10^{-24}	***
TIR/hAT	64.3942	1.07×10^{-14}	***	2.51×10^{-14}	***
TIR/CACTA	99.3991	3.87×10^{-21}	***	1.08×10^{-20}	***
TIR/PIF_Harbinger	17.5135	3.52×10^{-5}	***	5.47×10^{-5}	***
TIR/Tc1_Mariner	7.4279	0.0068	**	0.0087	**
Low_complexity	22.2882	3.22×10^{-6}	***	6.43×10^{-6}	***
Simple_repeat	0.4649	0.496	ns	0.496	ns
8s_rRNA	15.964	7.62×10^{-5}	***	1.07×10^{-4}	***

18s_rRNA	1.81564	0.179	ns	0.208	ns
28s_rRNA	21.4757	4.84×10^{-6}	***	8.46×10^{-6}	***

Tables S8: Analysis of variance (ANOVA) results for linear mixed-effects models of genome size in common ragweed (*Ambrosia artemisiifolia* L.), with geographic range, mean annual temperature (MAT), and genetic principal components (PC1, PC2) as fixed effects and population as a random intercept. The upper table shows the base model (main effects only); the lower table shows the model including the range × MAT interaction term. For each term, the table reports the F-statistic, numerator degrees of freedom (Df), denominator degrees of freedom (Df.res), P-value (Pr(>F)), and significance (Sig.). Significance codes: ***P < 0.001, **P < 0.01, *P < 0.05, ns = not significant.

	**F**	**Df**	**Df.res**	**Pr(>F)**	**Sig.**
range	18.7924	2	129.73	6.81×10^{-8}	***
MAT	8.2482	1	144.86	0.0047	**
PC1	0.5362	1	208.88	0.4648	ns
PC2	2.3229	1	214.9	0.1289	ns

	**F**	**Df**	**Df.res**	**Pr(>F)**	**Sig.**
range	0.7576	2	83.229	0.47199	ns
MAT	0.7225	1	68.005	0.39831	ns
PC1	0.1265	1	254.046	0.72234	ns
PC2	3.0169	1	247.501	0.08364	ns
range*MAT	0.3827	2	83.089	0.68325	ns

Tables S9: Linear quantile mixed model (LQMM) results for genome size across all geographic ranges of common ragweed (*Ambrosia artemisiifolia* L.), with geographic range, the flowering-time polygenic score (PGV_{FT}) and genetic principal components (PC1, PC2) included as fixed effects and population as a random effect. Models were fitted at five quantiles (tau = 0.1, 0.25, 0.5, 0.75, 0.9) of the genome size distribution. For each quantile, the table reports the model coefficient, standard error (SE), P-value, and whether the effect was statistically significant at P < 0.05 (marked with an asterisk in the 'Significant?' column).

Tau	Coefficient	SE	P-value	Significant?
0.1	-1.585	1.841	0.393	No
0.25	1.353	1.769	0.448	No
0.5	2.307	1.142	0.049	Yes*
0.75	2.376	1.645	0.155	No
0.9	3.499	1.881	0.069	No

Tables S10: Linear quantile mixed model (LQMM) results for genome size within each geographic range of common ragweed (*Ambrosia artemisiifolia* L.): native North America, invasive Europe, and invasive Australia, with the flowering-time polygenic score (PGV_{FT}) and genetic principal components (PC1, PC2) included as fixed effects and population as a random effect. Models were fitted at five quantiles (tau = 0.1, 0.25, 0.5, 0.75, 0.9) of the genome size distribution within each range. For each range and quantile, the table reports the model coefficient, standard error (SE), P-value, and whether the effect was statistically significant at P < 0.05 (marked with an asterisk in the 'Significant?' column).

Range	Tau	Coefficient	SE	P-value	Significant?
North America	0.1	-1.031	6.708	0.785	No
North America	0.25	0.9638	6.208	0.877	No
North America	0.5	0.9212	6.009	0.878	No
North America	0.75	2.6765	5.751	0.643	No
North America	0.9	-2.1231	6.113	0.729	No
Europe	0.1	2.59	2.714	0.345	No
Europe	0.25	1.405	1.573	0.376	No
Europe	0.5	1.77	1.37	0.203	No
Europe	0.75	-0.358	1.431	0.803	No
Europe	0.9	2.311	1.796	0.204	No
Australia	0.1	1.274	2.265	0.576	No
Australia	0.25	-0.012	1.927	0.995	No
Australia	0.5	0.684	1.916	0.723	No
Australia	0.75	2.153	1.757	0.226	No
Australia	0.9	3.613	1.59	0.027	Yes*

References:

1. Poptsova, M. S. et al. Non-random DNA fragmentation in next-generation sequencing. Scientific reports 4, 4532 (2014). https://doi.org/10.1038/srep04532
2. van Heesch, S. et al. Improving mammalian genome scaffolding using large insert mate-pair next-generation sequencing. BMC genomics 14, 257 (2013). https://doi.org/10.1186/1471-2164-14-257

3. Parks, M. M. et al. Variant ribosomal RNA alleles are conserved and exhibit tissue-specific expression. Science Advances 4, eaao0665 (2018). https://doi.org/doi:10.1126/sciadv.aao0665
4. Sims, J., Sestini, G., Elgert, C., von Haeseler, A. & Schlögelhofer, P. Sequencing of the Arabidopsis NOR2 reveals its distinct organization and tissue-specific rRNA ribosomal variants. Nature communications 12, 387 (2021). https://doi.org/10.1038/s41467-020-20728-6
5. Sultanov, D. & Hochwagen, A. Varying strength of selection contributes to the intragenomic diversity of rRNA genes. Nature Communications 13, 7245 (2022). https://doi.org/10.1038/s41467-022-34989-w
6. Li, H., Ruan, J. & Durbin, R. Mapping short DNA sequencing reads and calling variants using mapping quality scores. Genome Res 18, 1851-1858 (2008). https://doi.org/10.1101/gr.078212.108
7. Li, H. et al. The Sequence Alignment/Map format and SAMtools. Bioinformatics 25, 2078-2079 (2009). https://doi.org/10.1093/bioinformatics/btp352
8. Ajay, S. S., Parker, S. C., Abaan, H. O., Fajardo, K. V. & Margulies, E. H. Accurate and comprehensive sequencing of personal genomes. Genome Res 21, 1498-1505 (2011). https://doi.org/10.1101/gr.123638.111
9. Marshall, C. R. et al. Best practices for the analytical validation of clinical whole-genome sequencing intended for the diagnosis of germline disease. NPJ Genom Med 5, 47 (2020). https://doi.org/10.1038/s41525-020-00154-9
10. Bieker, V. C. et al. Uncovering the genomic basis of an extraordinary plant invasion. Science Advances 8, eabo5115 (2022). https://doi.org/doi:10.1126/sciadv.abo5115
11. Gilbert, D. G. Measuring DNA contents of animal and plant genomes with Gnodes, the long and short of it. bioRxiv, 2024.2010.2006.616888 (2024). https://doi.org/10.1101/2024.10.06.616888
12. Sliwinska, E. et al. Application-based guidelines for best practices in plant flow cytometry. Cytometry Part A 101, 749-781 (2022). https://doi.org/https://doi.org/10.1002/cyto.a.24499
13. Hrabovský, M., Kubalová, S., Mičieta, K. & Ščevková, J. Environmental impacts on intraspecific variation in Ambrosia artemisiifolia genome size in Slovakia, Central Europe. Environmental Science and Pollution Research 31, 33960-33974 (2024). https://doi.org/10.1007/s11356-024-33410-x
14. Bredeson, J. V. et al. Sequencing wild and cultivated cassava and related species reveals extensive interspecific hybridization and genetic diversity. Nature biotechnology 34, 562-570 (2016). https://doi.org/10.1038/nbt.3535
15. Awoleye, F., Van Duren, M., Dolezel, J. & Novak, F. Nuclear DNA content and in vitro induced somatic polyploidization cassava (Manihot esculenta Crantz) breeding. Euphytica 76, 195-202 (1994). https://doi.org/10.1007/BF00022164
16. Kubešová, M., Moravcova, L., Suda, J., Jarošík, V. & Pyšek, P. Naturalized plants have smaller genomes than their non-invading relatives: a flow cytometric analysis of the Czech alien flora. Preslia 82, 81-96 (2010). https://www.preslia.cz/article/225

17. Bai, C., Alverson, W. S., Follansbee, A. & Waller, D. M. New reports of nuclear DNA content for 407 vascular plant taxa from the United States. Annals of botany 110, 1623-1629 (2012). https://doi.org/10.1093/aob/mcs222
18. Battlay, P. et al. Large haploblocks underlie rapid adaptation in the invasive weed Ambrosia artemisiifolia. Nat Commun 14, 1717 (2023). https://doi.org/10.1038/s41467-023-37303-4
19. Doležel, J., Sgorbati, S. & Lucretti, S. Comparison of three DNA fluorochromes for flow cytometric estimation of nuclear DNA content in plants. Physiologia plantarum 85, 625-631 (1992). https://doi.org/10.1111/j.1399-3054.1992.tb04764.x
20. Greilhuber, J., Temsch, E. M. & Loureiro, J. C. Nuclear DNA content measurement. Flow cytometry with plant cells: analysis of genes, chromosomes and genomes, 67-101 (2007). https://doi.org/10.1002/9783527610921.ch4
21. Purcell, S. et al. PLINK: a tool set for whole-genome association and population-based linkage analyses. Am J Hum Genet 81, 559-575 (2007). https://doi.org/10.1086/519795
22. VanRaden, P. M. Efficient Methods to Compute Genomic Predictions. Journal of Dairy Science 91, 4414-4423 (2008). https://doi.org/https://doi.org/10.3168/jds.2007-0980
23. Amadeu, R. R., Garcia, A. A. F., Munoz, P. R. & Ferrão, L. F. V. AGHmatrix: genetic relationship matrices in R. Bioinformatics 39 (2023). https://doi.org/10.1093/bioinformatics/btad445
24. Nilforooshan, M. A. mbend: an R package for bending non-positive-definite symmetric matrices to positive-definite. BMC genetics 21, 97 (2020). https://doi.org/10.1186/s12863-020-00881-z
25. Hadfield, J. D. MCMC Methods for Multi-Response Generalized Linear Mixed Models: The MCMCglmm R Package. Journal of Statistical Software 33, 1 - 22 (2010). https://doi.org/10.18637/jss.v033.i02
26. Falconer, D. S. Introduction to quantitative genetics. (Pearson Education India, 1996). https://doi.org/10.1093/genetics/167.4.1529
27. Lynch, M. & Walsh, B. Genetics and analysis of quantitative traits. Vol. 1 (Sinauer Sunderland, MA, 1998). https://www.invemar.org.co/redcostera1/invemar/docs/RinconLiterario/2011/febrero/AG_8.pdf
28. de Villemereuil, P. Quantitative genetic methods depending on the nature of the phenotypic trait. Annals of the New York Academy of Sciences 1422, 29-47 (2018). https://doi.org/10.1111/nyas.13571
29. Perrier, C., Delahaie, B. & Charmantier, A. Heritability estimates from genomewide relatedness matrices in wild populations: Application to a passerine, using a small sample size. Molecular ecology resources 18, 838-853 (2018). https://doi.org/10.1111/1755-0998.12886
30. Wilson, A. J. et al. An ecologist's guide to the animal model. Journal of animal ecology 79, 13-26 (2010). https://doi.org/10.1111/j.1365-2656.2009.01639.x
31. Spitze, K. Population structure in Daphnia obtusa: quantitative genetic and allozymic variation. Genetics 135, 367-374 (1993). https://doi.org/10.1093/genetics/135.2.367
32. Lande, R. Neutral theory of quantitative genetic variance in an island model with local extinction and colonization. Evolution 46, 381-389 (1992). https://doi.org/10.1111/j.1558-5646.1992.tb02046.x

33. Whitlock, M. C. Evolutionary inference from QST. Molecular ecology 17, 1885-1896 (2008). https://doi.org/10.1111/j.1365-294x.2008.03712.x
34. Whitlock, M. C. & Guillaume, F. Testing for spatially divergent selection: comparing QST to FST. Genetics 183, 1055-1063 (2009). https://doi.org/10.1534/genetics.108.099812
35. Leinonen, T., McCairns, R. S., O'hara, R. B. & Merilä, J. Q ST–F ST comparisons: evolutionary and ecological insights from genomic heterogeneity. Nature Reviews Genetics 14, 179-190 (2013). https://doi.org/10.1038/nrg3395
36. Prout, T. & Barker, J. S. F statistics in Drosophila buzzatii: selection, population size and inbreeding. Genetics 134, 369-375 (1993). https://doi.org/10.1093/genetics/134.1.369
37. Sæther, S. A. *et al.* Inferring local adaptation from QST–FST comparisons: neutral genetic and quantitative trait variation in European populations of great snipe. Journal of Evolutionary Biology 20, 1563-1576 (2007). https://doi.org/10.1111/j.1420-9101.2007.01328.x
38. Bates, D., Mächler, M., Bolker, B. & Walker, S. Fitting Linear Mixed-Effects Models Using lme4. Journal of Statistical Software 67, 1 - 48 (2015). https://doi.org/10.18637/jss.v067.i01
39. BROMMER, J. E. Whither Pst? The approximation of Qst by Pst in evolutionary and conservation biology. Journal of Evolutionary Biology 24, 1160-1168 (2011). https://doi.org/https://doi.org/10.1111/j.1420-9101.2011.02268.x
40. Sun, Y., Bossdorf, O., Grados, R. D., Liao, Z. & Müller-Schärer, H. Rapid genomic and phenotypic change in response to climate warming in a widespread plant invader. Global Change Biology 26, 6511-6522 (2020). https://doi.org/10.1111/gcb.15291
41. Josephs, E. B., Berg, J. J., Ross-Ibarra, J. & Coop, G. Detecting adaptive differentiation in structured populations with genomic data and common gardens. Genetics 211, 989-1004 (2019). https://doi.org/10.1534/genetics.118.301786
42. Korneliussen, T. S., Albrechtsen, A. & Nielsen, R. ANGSD: Analysis of Next Generation Sequencing Data. BMC Bioinformatics 15, 356 (2014). https://doi.org/10.1186/s12859-014-0356-4
43. Battlay, P. et al. Rapid Parallel Adaptation in Distinct Invasions of Ambrosia Artemisiifolia Is Driven by Large-Effect Structural Variants. Molecular Biology and Evolution 42 (2025a). https://doi.org/10.1093/molbev/msae270
44. Meisner, J. & Albrechtsen, A. Inferring population structure and admixture proportions in low-depth NGS data. Genetics 210, 719-731 (2018). https://doi.org/10.1534/genetics.118.301336
45. Kreiner, J. M., Hnatovska, S., Stinchcombe, J. R. & Wright, S. I. Quantifying the role of genome size and repeat content in adaptive variation and the architecture of flowering time in Amaranthus tuberculatus. PLoS genetics 19, e1010865 (2023). https://doi.org/10.1371/journal.pgen.1010865
46. Fick, S. E. & Hijmans, R. J. WorldClim 2: new 1-km spatial resolution climate surfaces for global land areas. International Journal of Climatology 37, 4302-4315 (2017). https://doi.org/https://doi.org/10.1002/joc.5086
47. Geraci, M. Linear Quantile Mixed Models: The lqmm Package for Laplace Quantile Regression. Journal of Statistical Software 57, 1 - 29 (2014). https://doi.org/10.18637/jss.v057.i13

48. van Boheemen, L. A., Atwater, D. Z. & Hodgins, K. A. Rapid and repeated local adaptation to climate in an invasive plant. New Phytologist 222, 614-627 (2019). https://doi.org/https://doi.org/10.1111/nph.15564
